# The Limits to Parapatric Speciation II: Strengthening a Preexisting Genetic Barrier to Gene Flow in Parapatry

**DOI:** 10.1534/genetics.117.300652

**Published:** 2018-02-28

**Authors:** Alexandre Blanckaert, Joachim Hermisson

**Affiliations:** * Department of Mathematics, University of Vienna, 1090, Austria; † Instituto Gulbenkian de Ciência, 2780-156 Oeiras, Portugal; ‡ Mathematics and Biosciences Group, Max F. Perutz Laboratories, 1030 Vienna, Austria

**Keywords:** allopatric, Dobzhansky–Muller, gene flow, genetic incompatibilities, model, parapatric, speciation

## Abstract

By encompassing the whole continuum between allopatric and sympatric scenarios, parapatric speciation includes many potential scenarios for the evolution of new species. Here, we investigate how a genetic barrier to gene flow, that relies on a single postzygotic genetic incompatibility, may further evolve under ongoing migration. We consider a continent island model with three loci involved in pairwise Dobzhansky–Muller incompatibilities (DMIs). Using an analytic approach, we derive the conditions for invasion of a new mutation and its consequences for the strength and stability of the initial genetic barrier. Our results show that the accumulation of genetic incompatibilities in the presence of gene flow is under strong selective constraints. In particular, preexisting incompatibilities do not always facilitate the invasion of further barrier genes. If new mutations do invade, they will often weaken or destroy the barrier rather than strengthening it. We conclude that migration is highly effective at disrupting the so-called “snowball effect”, the accelerated accumulation of DMIs that has been described for allopatric populations en route to reproductive isolation.

UNDER what conditions can geographically separated populations that are connected by migration build up a genetic barrier to gene flow? When and how can this barrier be strengthened and eventually lead to speciation? Following the increasing awareness that gene flow and hybridization between related (incipient) species is ubiquitous in both plants and animals (Mallet 2005; Butlin *et al.* 2008), these long-standing questions of parapatric speciation research are receiving renewed interest (Bank *et al.* 2012; Marie Curie Speciation Network *et al.* 2012; Flaxman *et al.* 2013, 2014; Paixão *et al.* 2014; Seehausen *et al.* 2014; Barnard-Kubow *et al.* 2016; Kulmuni and Westram 2017; Nosil *et al.* 2017; Yang *et al.* 2017). Answers to these questions strongly depend on the speciation mechanism that is considered. On the one hand, there are scenarios of “adaptive speciation” (Dieckmann *et al.* 2004; Weissing *et al.* 2011), where speciation (or the build-up of a genetic barrier) is a direct target of selection. The genetic barrier in this case is usually prezygotic and can result from the evolution of assortative mating. If speciation is driven by local competition [as in the classical scenario of sympatric speciation, Dieckmann and Doebeli (1999)], the probability or speed of speciation is unaffected by migration. Alternatively, if assortative mating evolves as a response against mating with maladaptive immigrants, migration is driving speciation in the first place (Servedio and Noor 2003; Rettelbach *et al.* 2013). On the other hand, other scenarios consider speciation as a nonselected by-product of neutral or adaptive divergence. In particular, this is how reproductive isolation evolves in classical models of allopatric speciation (Orr 1995; Orr and Turelli 2001; Coyne and Orr 2004). In contrast to the scenarios of adaptive speciation, in that case, migration acts against the build-up of a genetic barrier. Given that models of adaptive speciation require specific assumptions about the selection scheme and given the ubiquitous nature of gene flow, the question arises whether and when speciation as a by-product can occur in a parapatric model.

Following previous work (Bank *et al.* 2012; Flaxman *et al.* 2013; Aeschbacher and Bürger 2014; Akerman and Bürger 2014; Fraïsse *et al.* 2014; Paixão *et al.* 2014; Höllinger and Hermisson 2017), we study the conditions for the emergence of a postzygotic barrier to gene flow between parapatric populations. Two mechanisms can contribute to the build-up of such a barrier: local adaptation and genetic incompatibilities (Schluter 2009; Bank *et al.* 2012; Kulmuni and Westram 2017). Local adaptation and divergence driven by ecological differences among the populations is arguably the easiest mechanism to create a barrier in the presence of gene flow (Flaxman *et al.* 2013; Akerman and Bürger 2014). Any new mutation with a local fitness advantage larger than the migration rate can establish in the population. If this same mutation is detrimental in the other environment, the adaptation remains local and contributes to a fitness deficit of migrants. An increasing number of local adaptation genes along the chromosome can strengthen the barrier and reduce the effective rates of gene flow among populations. Speciation in the sense of full reproductive isolation corresponds to the limit where immigrants are “dead on arrival.” However, hybridization remains possible whenever populations can overlap at all, in any environment (or laboratory) where both are viable. This problem is avoided if the genetic barrier is due to genetic incompatibilities and selection acts primarily on hybrids rather than on (first-generation) migrants. This is the insight of the Bateson–Dobzhanszy–Muller model (Bateson 1909; Dobzhansky 1936; Muller 1942) that has since become the standard model to explain speciation in an allopatric setting (Orr and Turelli 2001; Coyne and Orr 2004).

The two mechanisms, selection against migrants (*i.e.*, local adaptation) and selection against hybrids (Dobzhansky–Muller incompatibilities, DMIs) are nonexclusive (Kulmuni and Westram 2017). In particular, whereas neutral DMIs cannot evolve in a parapatric setting (Gavrilets 1997; Bank *et al.* 2012), DMIs can still evolve and be maintained if at least one of the incompatible alleles is also locally adaptive. Considering a continent–island scenario, Bank *et al.* (2012) characterized the conditions under which a simple two-locus DMI can originate and be maintained in the face of gene flow, a very first step on the route to (potential) speciation. Here, we ask how this process can continue. Under which conditions will further substitutions in either population strengthen or weaken (or even destroy) an existing genetic barrier? It turns out that the answer to this question is surprisingly complex, depending on patterns of epistasis and on the genetic architecture and linkage pattern of the barrier genes involved. We discuss the potential of a new mutation to strengthen a barrier and whether it is a step toward reproductive isolation. Lastly, we characterize the genetic architecture that produced the strongest genetic barrier under gene flow and relate these results to the recent discussion of so-called “islands of divergence” (Via and West 2008; Feder *et al.* 2012a).

## Methods

To study the accumulation of incompatibilities in the presence of gene flow, we use a migration–selection model in continuous time with three loci. We consider two panmictic populations, one on a continent and the other on an island, each of sufficient size such that we can ignore the effects of genetic drift. There is unidirectional migration from the continental population to the island population at rate *m*. Selection acts on three loci, 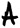, 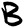, and 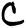, with two alleles each (**A/a**, **B/b**, and **C/c**). Lower case letters indicate the ancestral state, upper case letters are derived alleles. We study both haploid and diploid populations. We always assume that the continent is fixed for a unique genotype; substitutions on the continent can occur, but they are instantaneous and do not lead to a persistent polymorphism. We focus on the migration–selection dynamics on the island, where all three loci can be polymorphic.

### Haploid model

There are $2^{3}=8$ different haplotypes with frequencies $x_{1},x_{2},\ldots x_{8}.$ In particular, $x_{1}$ is the frequency of the ancestral genotype **abc**, with Malthusian (or log-) fitness normalized to 0. We have three parameters for single-locus fitness effects, α, β, and γ. Three parameters, $\epsilon_{AB},$ $\epsilon_{BC},$ and $\epsilon_{AC},$ parametrize potential pairwise epistasis between derived alleles (see Table 1). Restrictions on epistasis values are detailed below.

**Table 1:** Frequencies $x_{i}$ and fitness values $w_{i}$ of the different haplotypes for haploid populations

Hap.	abc	Abc	aBc	abC	ABc	AbC	aBC	ABC
$x_{i}$	$x_{1}$	$x_{2}$	$x_{3}$	$x_{4}$	$x_{5}$	$x_{6}$	$x_{7}$	$x_{8}$
$w_{i}$	0	α	β	γ	α + β	α + γ	β + γ	α + β + γ
					+ $\epsilon_{AB}$	+ $\epsilon_{AC}$	+ $\epsilon_{BC}$	+ $\epsilon_{AB}$ + $\epsilon_{BC}$ + $\epsilon_{AC}$

We always assume α>0 and $\epsilon_{AB}<0$.

In the following, we assume that each locus has a specific role. In particular, we assume that allele **A** is always an island adaptation (allele **A** appears on the island). As a consequence, α is always strictly positive. In contrast, allele **B** is always a continental adaptation (allele **B** appears on the continent). There is no constraint on its selective advantage, β, on the island: both negative and positive values are investigated. We always assume that **A** and **B** are incompatible, *i.e.*, $\epsilon_{AB}<0.$ While loci 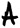 and 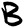 form the nucleus of a genetic barrier that exists initially, any further extension of this barrier occurs on the 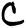 locus. At this locus, the new allele **C** can appear either on the island or on the continent. There is no constraint on its selective advantage, γ. **C** can interact positively or negatively with the other derived alleles. To keep our model tractable, we only allow for epistasis between island and continental adaptations. In other words, if **C** appears on the island, it only interacts with the continental adaptation **B** ($\epsilon_{AC}=0$). Similarly, if **C** appears on the continent, epistasis only occurs between **A** and **C** ($\epsilon_{BC}$ = 0). This excludes schemes of complex epistasis with interactions among all three locus pairs, or higher-order interactions.

Note that our choice for the role of loci 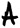, 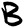, and 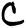 is made to reduce the parameter space. Alternative scenarios can be easily deduced through reparametrization of the system, given in Supplemental Material, Table A3 in File S1. Since the model is defined in continuous time, all parameters for selection or migration are rates. For the derivation of equilibria, only relative rates matter. In particular, we can scale all parameters by the selection coefficient α of the **A** allele (which is always >0).

The three loci 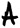, 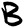, and 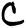 can be located in any order along the genome. The full system with arbitrary linkage, given in Equation (A2) in File S1, is not tractable analytically. In our analysis, we therefore focus on limiting cases with pairs of loci either in tight linkage (recombination rate r→0) or in loose linkage. In our model, we implement loose linkage as the limit r→∞, which implies that the corresponding loci are always in linkage equilibrium. We relax this assumption in Figures C24 and C25 in File S3, where we discuss numerical results for the dynamics with intermediate recombination. The linkage equilibrium approximation holds as soon as recombination is stronger than the other evolutionary forces (selection and migration). This gives rise to five different linkage architectures: 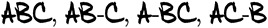, and 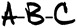, where “-” denotes loose linkage and its absence tight linkage. We investigate these architectures both for **C** appearing on the island or on the continent respectively, leading to 10 different cases.

The dynamic equations for the allele frequencies on the island ($p_{A},p_{B},p_{C}$ for alleles **A**, **B**, and **C**, respectively) for all cases are derived in the Equations (A6)–(A8) in File S1. For example, we obtain for loose linkage (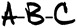),

$$\begin{array}{l} \dot{p_{A}}=p_{A}\left(\left(1-p_{A}\right)\left(\alpha +p_{B}\epsilon_{AB}+p_{C}\epsilon_{AC}\right)-m\right)\\ \dot{p_{B}}=p_{B}\left(\left(1-p_{B}\right)\left(\beta +p_{A}\epsilon_{AB}+p_{C}\epsilon_{BC}\right)-m\right)+m\\ \dot{p_{C}}=p_{C}\left(\left(1-p_{C}\right)\left(\gamma +p_{A}\epsilon_{AC}+p_{B}\epsilon_{BC}\right)-m\right)+m_{C} \end{array}$$ (1)

where $m_{C}=m$ or $m_{C}$ = 0, depending on whether **C** appears on the continent or on the island.

### Diploid model

We define the fitness scheme for diploids as follows: single-locus effects (*i.e.*, α,β,γ) are purely additive. There is thus no dominance at this level. However, dominance is included for epistasis. Following previous work (Turelli and Orr 2000; Bank *et al.* 2012), we assume that the strength of epistasis depends only on the number of incompatible pairs in a genotype, *e.g.*, **AB/Ab** generates the same epistasis as **AB/aB**.

We investigate two cases of dominance of the epistatic interaction: recessive and codominant epistasis (see Table 2). Assuming Hardy–Weinberg equilibrium on the island, the dynamic system for diploids coincides with the haploid equation [given in Equation (A2) in File S1] if we replace the fitness of all haplotypes by the corresponding marginal fitness (${\bar{w}}_{i}=\sum_{j=1}^{8}x_{ij}w_{ij}$). In the case of the codominant model, the diploid dynamics reduce to the dynamics of the haploid model if all interacting loci are in loose linkage (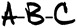 as well as 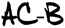 if **C** appears on the island, and 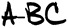 if **C** appears on the continent). The different systems of equations are available in the Equations (A11)–(A15) in File S1.

**Table 2:** Section of the fitness table specifying the interactions between the A and B alleles in the background of allele c for codominant (top) and recessive (bottom) epistasis

	Abc	aBc	ABc
Abc	2α	$\alpha +\beta +\epsilon_{AB}/2$	$2\alpha +\beta +\epsilon_{AB}$
aBc		2β	$\alpha +2\beta +\epsilon_{AB}$
ABc			$2\alpha +2\beta +2\epsilon_{AB}$
			
	Abc	aBc	ABc
Abc	2α	α+β	$2\alpha +\beta +\epsilon_{AB}$
aBc		2β	$\alpha +2\beta +\epsilon_{AB}$
ABc			$2\alpha +2\beta +2\epsilon_{AB}$

Interactions between A and C as well as B and C are analogous (the complete table is available in Table A2 in File S1).

### Strength of the genetic barrier

There are multiple measures for the strength of a genetic barrier between two divergent populations that are connected by gene flow. For example, the gene-flow factor (or the effective migration rate) due to Barton and Bengtsson (1986) measures the reduced probability of neutral alleles that are linked to barrier genes to cross this barrier and establish in the recipient population. Here, we consider the fate of barrier genes themselves. In particular, we are interested in the maximum rate of gene flow under which a barrier (with given selection parameters) can be built and also in the maximum rate of gene flow under which such a barrier can persist if it exists initially.

Specifically, we define the barrier strength $m_{\max}^{X}$ for a given set of barrier loci as the maximal migration rate under which a set X of alleles at these loci can still be maintained on the island. Here, X denotes the barrier alleles that are not present on the continent, but are maintained on the island as long as migration is below the threshold (m< $m_{\max}^{X}$). For example, for a single-locus barrier with the **A** allele on the island, we have X = **A** and the strength of the genetic barrier is given by $m_{\max}^{A}.$ For m< $m_{\max}^{A},$ the 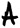 locus is polymorphic on the island, for m> $m_{\max}^{A},$ the **A** allele is swamped and the locus is fixed for the continental **a** allele. Analogously, the strength of a genetic barrier with three polymorphic loci and island alleles **A**, **b**, and **C** (say) is denoted as $m_{\max}^{AbC}.$ The two-locus barrier $m_{\max}^{+}$ from Bank *et al.* (2012) corresponds to $m_{\max}^{Ab}$ with this notation.

Below, we consider how the strength of an existing genetic barrier changes under further evolution. We then denote the original barrier strength, which serves as the reference point, as $m_{\max,0}^{X}$ (*e.g.*, $m_{\max,0}^{Ab}$ is the initial strength of an 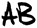 barrier with the third locus 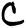 fixed for its ancestral allele **c**). While m< $m_{\max}^{X}$ guarantees that an existing DMI is not swamped, the origin of the DMI may require a favorable evolutionary history (mutation order) or an initial allopatric phase, (Bank *et al.* 2012).

### Data availability

The authors state that all data necessary for confirming the conclusions presented in the article are represented fully within the article.

## Results

### Adaptation at existing barrier loci

In the first part of the results section, we study the case where further adaptation happens directly at an already existing barrier locus (*i.e.*, at a locus 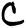 in tight linkage to such a locus). In particular, we compare the simple case of further adaptation at a single barrier locus with the more complex scenario where adaptation happens at a barrier locus that is involved in a two-locus incompatibility.

#### Further adaptation at a single-locus barrier:

Assume that, initially, 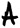 is the only polymorphic locus. The initial barrier strength is α and results entirely from local adaptation ($m_{\max,0}^{A}$ =α). A new mutation occurs at a tightly linked locus, 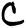. This scenario is equivalent to adaptation at a single compound locus 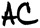 with alleles **Ac** and **ac**, and mutation generating new alleles **AC** with fitness α+γ and **aC** with fitness γ. At most, two alleles can be maintained on the island (Nagylaki and Lou 2001): the continental allele and the allele with the highest fitness on the island. Thus, any new adaptation on the island that produces a better allele than **Ac** will replace this allele (*e.g.*, the **AC** allele for γ>0). While any successful adaptation on the island increases the barrier strength ($m_{\max}^{AC}$ =α+γ), adaptation on the continent can lead to a stronger or weaker barrier ($m_{\max}^{A}$ =α−γ), depending on whether γ is positive or negative. In particular, $m_{\max}^{A}$ ≤0 means that no polymorphism can be maintained. However, if there is a small but nonzero recombination probability among 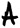 and 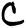, any adaptation on the continent will eventually also enter the island background. We then have two new alleles **AC** and **aC** replacing the old ones (**Ac** and **ac**) and the barrier strength $m_{\max}^{A}$ =α remains unchanged as long as there is no epistasis among **A** and **C**.

We thus see that further adaptation at a single polymorphic locus will usually strengthen the genetic barrier, rather than weaken it. In particular, this holds for any further adaptation on the island. A three-locus architecture, 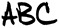, with tight linkage among all three loci leads to an analogous single-locus problem (after appropriate relabeling of parameters).

Note that the genetic barrier formed by a single locus relies exclusively on local adaptation: any isolation observed is due to the impossibility of coexisting in a common environment and not due to a genetic mechanism. This is different for barriers with multiple interacting loci, which is our focus in the remainder of the manuscript.

#### Further adaptation at a two-locus barrier:

Assume now that we start with a two-locus polymorphism at two incompatible loci 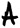 and 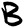 (a two-locus DMI) in loose linkage. The continental haplotype is **aB**, and **Ab** is the fittest haplotype on the island. A new mutation appears on the island at locus 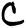 in tight linkage with 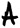. As discussed in the previous section, this generates a compound locus 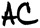. The new mutation generates a third allele at this compound locus (*e.g.*, **AC**), which we will call the **A’** allele in the following. We denote the fitness advantage of the new allele **A’** as α’ and its epistatic interaction with the **B** allele at the 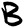 locus as $\epsilon_{A^{\prime }B}.$ This leads to the dynamics of a triallellic locus (with alleles **a**, **A**, and **A’**) that interacts with a loosely linked biallelic locus in the genomic background (alleles **b** and **B**):

$$\begin{array}{c} \dot{p_{A}}=p_{A}\left(\left(1-p_{A}\right)\left(\alpha +p_{B}\epsilon_{AB}\right)-p_{A\prime }\left(\alpha^{\prime }+p_{B}\epsilon_{A\prime B}\right)-m\right)\\ \dot{p_{A^{\prime }}}=p_{A^{\prime }}\left(\left(1-p_{A^{\prime }}\right)\left(\alpha^{\prime }+p_{B}\epsilon_{A^{\prime }B}\right)-p_{A}\left(\alpha +p_{B}\epsilon_{AB}\right)-m\right)\\ \dot{p_{B}}=\left(1-p_{B}\right)\left(p_{B}\left(\beta +p_{A}\epsilon_{AB}+p_{A\prime }\epsilon_{A^{\prime }B}\right)+m\right) \end{array}$$ (2)

For tight linkage, we can assume that a fourth allele **A”** (*e.g.*, **A”** = **aC**) will only originate by mutation or rare recombination after one of the alleles **a**, **A**, or **A’** is lost. This leads again to the three-allele dynamics described by Equation (2). Results for the four-allele dynamics are given in section C2.4 in File S3. Further scenarios with adaptation at the 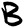 locus or continental adaptation at the 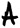 locus are discussed in section B7 in File S2.

The dynamic system given in Equation (2) allows up to nine equilibria, up to three of which can be simultaneously stable. In the section B2 in File S2, we show that alleles **A** and **A’** can never coexist at a stable equilibrium [extending the single-locus result of Nagylaki and Lou (2001)]. Nevertheless, interaction of 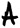 with an unlinked locus 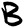 considerably adds to the complexity and can lead to qualitatively different results.

**Figure 1:**
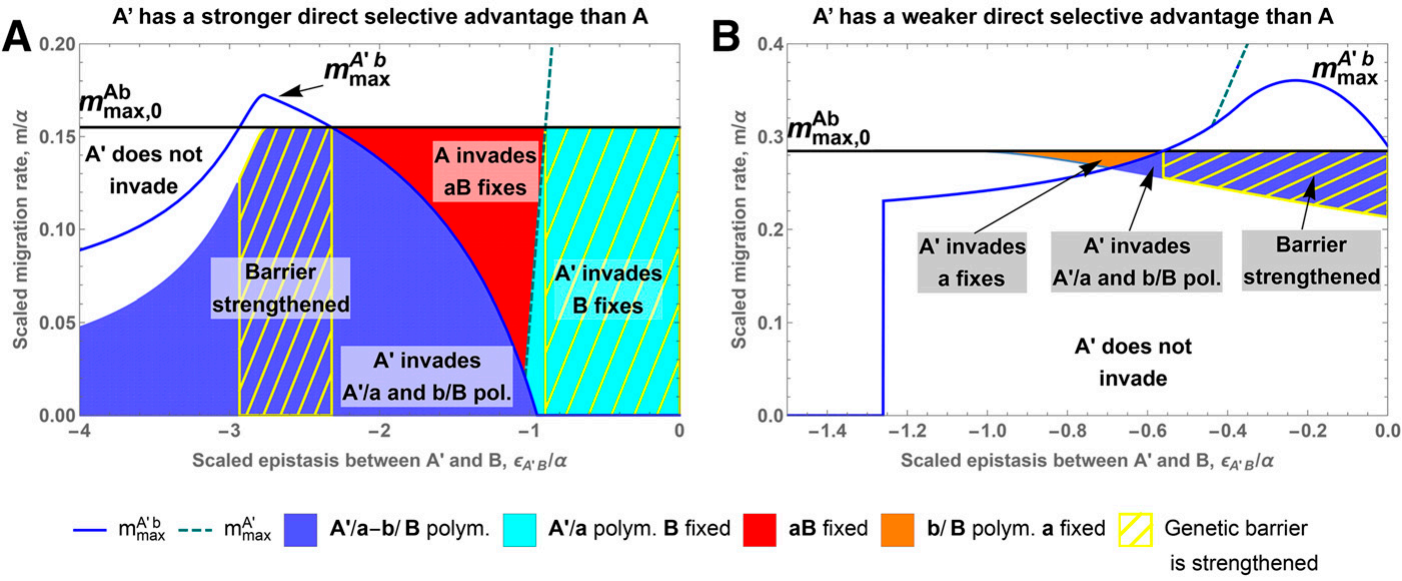
The impact of the invasion of a new allele **A’** at locus 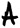 on an existing two-locus barrier (loci 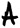, 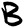 in loose linkage). We show the strength of a genetic barrier against swamping for a new allele **A’** as a function of its (scaled) epistatic coefficient. The strength of the original genetic barrier is indicated by the black line. In both examples, we have $m_{\max,0}^{Ab}$ = $m_{\max,0}^{A}.$ Invasion of allele **A’** can only happen in a finite interval for *m* (Equation (B7) in File S2 for explicit expressions), corresponding to the colored area. There are four possible outcomes to the successful invasion of the **A’** allele, denoted by the background color: **A’** replaces **A** and **b** remains present (in blue), **A’** replaces **A** but allele **B** fixes (in cyan), the polymorphism at locus 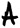 is lost (orange), and the continental haplotype fixes (red). If **A’** successfully replaces **A**, the new two-locus barrier strength, $m_{\max}^{A^{\prime }b},$ is given by the blue line. The dashed cyan line shows the strength of the new single-locus barrier, $m_{\max}^{A^{\prime }},$ whenever the 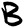 locus is swamped and $m_{\max}^{A^{\prime }}$ > $m_{\max}^{A^{\prime }b}.$ The yellow hatched area indicates that the genetic barrier at the 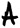 locus is strengthened by the invasion of allele **A’**. Panel A is obtained for $\beta /\alpha =0.95,\alpha^{\prime }/\alpha =1.05\;\text{and}\;\epsilon_{AB}/\alpha =-2.5$ and panel B for $\beta /\alpha =-0.28,\alpha^{\prime }/\alpha =0.75\;\text{and}\;\epsilon_{AB}/\alpha =-2.1.$

Whereas **A** and **A’** cannot coexist, allele **A’** can still invade the equilibrium formed by the DMI between loci 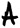 and 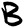. In contrast to the single-locus case, the potential for **A’** to invade no longer depends only on the fitness values, but also on the strength of migration (analytical expressions of the bounds are given in Equation B7 in File S2). In Figure 1, invasion of **A’** is possible in all colored regions. Figure 1A shows invasion of an allele **A’** with larger direct effect $\alpha^{\prime }>\alpha .$ If negative epistasis is less severe for **A’** than for **A** ($\epsilon_{AB}<\epsilon_{A\prime B}<0$), **A’** will always invade (*i.e.*, up to the maximal migration rate, $m_{\max,0}^{Ab},$ of the original two-locus polymorphism). However, for strong negative epistasis of the new allele ($\epsilon_{A^{\prime }B}<\epsilon_{AB}<0$), invasion of **A’** is only possible for weak migration $m\ll m_{\max,0}^{Ab}$ and a sufficiently low frequency of the competing **B** allele on the island. Figure 1B shows that the **A’** allele can also invade if its direct effect is weaker ($\alpha^{\prime }<\alpha$), provided that negative epistasis is also weaker ($\epsilon_{AB}<\epsilon_{A^{\prime }B}<0$). This requires that migration is sufficiently strong, because the marginal fitness of **A’** becomes larger than the marginal fitness of **A** only for a sufficiently large frequency of **B** alleles.

Successful invasion of **A’** can have qualitatively different outcomes, indicated by the different colors in Figure 1. In many cases, an invading **A’** allele displaces the old **A** allele and the system settles at a new equilibrium with an **a**/**A’** polymorphism. The new equilibrium can either be a two-locus polymorphism (blue areas in Figure 1) or a single-locus polymorphism with the 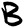 locus fixed for the **B** allele (cyan area). In both cases, the strength of the genetic barrier with respect to swamping can either increase (blue or cyan line above the black line) or decrease (blue or cyan line below the black line). Parameter ranges where invasion leads to a stronger genetic barrier, $m_{\max}^{A^{\prime }b}$ > $m_{\max,0}^{Ab},$ or $m_{\max}^{A^{\prime }}$ > $m_{\max,0}^{A},$ are indicated by yellow hatches.

Strengthening of the two-locus barrier (blue area with yellow hatches in Figure 1) can be due to two mechanisms. First, selection against migrants can be stronger due to additional local adaptation ($\alpha^{\prime }>\alpha$) and therefore leads to a larger fitness deficit ($\alpha^{\prime }-\beta$) for the continental haplotype on the island. This is the same mechanism as for the single-locus case. The genetic barrier is strengthened as long as epistasis, $\epsilon_{A^{\prime }B},$ does not deviate too much from the epistasis generated by the previous allele, $\epsilon_{AB}$ (Figure 1A, blue line above the black line). Indeed, if epistasis is too weak, the boost provided by the increased selection against migrants is negated by the weakening of selection against hybrids (since β>0). If epistasis is too strong, on the other hand, the marginal fitness of allele **A’** is decreased due to the increased cost of hybrids. Allele **A** can invade such an equilibrium as soon as migration increases and **A’** cannot strengthen the genetic barrier (see also section B6 in File S2).

The alternative mechanism corresponds to the reduction of selection against hybrids (Figure 1B). It works only if the continental **B** allele is deleterious on the island. Indeed, in this scenario, selection against hybrids does not contribute to the genetic barrier, as **B** is already maladaptive on the island. Nevertheless, epistasis still generates a cost for the island adaptation through the production of hybrids. Therefore, releasing the selective pressure on locus 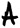 due to the hybrid cost ($\epsilon_{AB}\ll \epsilon_{A^{\prime }B}$) can strengthen the genetic barrier, even if this relief is associated with a reduction of the direct selective advantage of the island adaptation ($\alpha^{\prime }<\alpha$). The reduction of the selection against migrants is here compensated by the much lower hybrid cost paid by allele **A’** relative to allele **A**.

In contrast to the single-locus case, invasion of **A’** does not imply that this allele is maintained in the population. Indeed, we find significant parameter regions, where the following scenario happens. First, allele **A’** invades the island population (at its initial equilibrium with two-locus polymorphism), leading to the loss of the **A** allele. In the absence of allele **A**, allele **B** is no longer repressed and increases in frequency, making it impossible for allele **A’** to maintain itself in the population. Consequently, the continental **a** allele swamps the island and the polymorphism at the 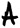 locus is lost altogether (red and orange are as in Figure 1). Again, the polymorphism at the 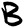 locus can either be maintained (orange area, Figure 1B) or destroyed (red, Figure 1A). Clearly, a necessary condition for such behavior is that the original two-locus polymorphism is not globally stable in the original **a**/**A**, **b**/**B** state space, but bistable together with an equilibrium with the **a** allele fixed. Numerical evidence strongly suggests that the fate of an invading **A’** allele depends on the existence of a stable **a/A’** polymorphism in the state space spanned by **a**/**A’**, **b**/**B**. If it does, the **A’** allele will eventually establish (as discussed above); if it does not, the **a** allele will take over (we did not find a case where the **A** allele would return and displace **A’** once the latter has been able to invade; see also section B3 in File S2 for a more detailed discussion and some proofs for specific cases).

**Figure 2:**
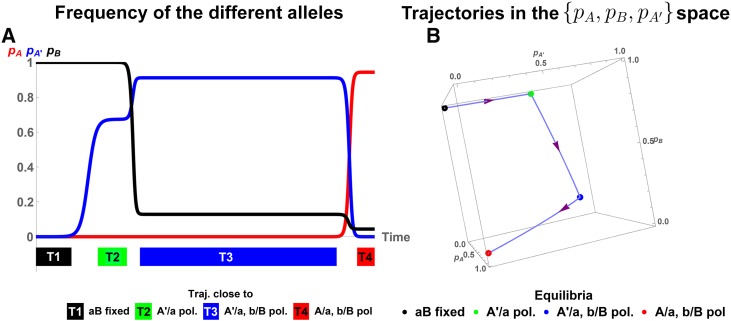
Evolutionary trajectory with **A’** as transient state. Panel A Frequency of derived alleles **B** (black), **A** (red), and **A’** (blue) as a function of time. At *t* = 0, the population is almost monomorphic for the continental haplotype **aB**, with both the alleles **A** and **A’** present at an extremely low frequency ($\approx 10^{-6}$). Colored blocks T1–T4 indicate when the population is close to an equilibrium, with the color matching the corresponding equilibrium. Panel B We represent the same trajectory in the $\left\{p_{A},p_{B},p_{A^{\prime }}\right\}$ space. Dots indicate equilibria, as indicated. Arrows indicate the evolutionary trajectory. Parameters used are: $\beta /\alpha =13/300,\alpha^{\prime }/\alpha =2/3,\epsilon_{AB}/\alpha =-4/3,\epsilon_{A^{\prime }B}/\alpha =-151/300,m/\alpha =4/75.$ One can observe a similar behavior with locus B starting polymorphic and allele **B** deleterious on the island (see Figure B2 in File S2).

A new allele **A’** can thus function as a temporary state that enables switching among different equilibria of the original two-locus two-allele system. In the examples discussed above, temporary invasion of **A’** will destroy a DMI polymorphism in this case. However, we also observe the opposite phenomenon: invasion of **A’** may create an **a/A**-**b/B** polymorphism rather than destroying it. This is illustrated in Figure 2. Bank *et al.* (2012) described the origin of such a DMI as a result of secondary contact (or a similar starting condition). Here, we provide an alternative explanation that does not require an interruption of gene flow.

We can compare the consequences of further adaptation on the island at an existing genetic barrier in the two cases discussed so far: a single polymorphic locus 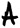 and a polymorphic locus 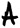 that interacts with a second polymorphic locus 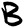. There are two notable differences. First, while further adaptation on the island always leads to a stronger barrier in the single-locus case, this is not the case for a two-locus barrier. Furthermore, invasion of a new allele no longer even guarantees establishment of this allele. On the contrary, we see that such an event can erase the existing barrier entirely. Second, the potential to strengthen the genetic barrier does not only depend on the fitness landscape, but also on the migration rate. Suppose that an allele **A’** exists that leads to a stronger barrier than **A**, if it invades. Figure 1 shows that invasion may either require sufficiently weak (Figure 1A) or sufficiently strong migration (Figure 1B). The latter scenario leads to the interesting observation that stronger gene flow can sometimes trigger the evolution of stronger barriers to gene flow [in Figure 1B, $m_{\max}^{A^{\prime }b}>$ $m_{\max,0}^{Ab},$ the blue line is above the black line; invasion of the new mutant is only possible with relatively strong migration (colored area in the figure)].

We also observe a general trend to replace a polymorphism that is maintained by selection against hybrids by one that is maintained due to selection against immigrants. Indeed, whereas it is possible to strengthen the genetic barrier by weakening the strength of epistasis without affecting the amount of local adaptation, the opposite is impossible. Any increase in the strength of selection against hybrids needs to be associated to some increase in local adaptation.

### Extension of the genetic barrier

We now turn to the extension of a genetic barrier by adaptation at an interacting locus 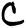 that is far away from the existing barrier loci and only loosely linked. We start with going from one to two loci and then study the case when a third locus is added.

#### Extension of a single-locus genetic barrier:

Assume that 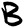 is the only polymorphic locus on the island (β<0, therefore **$m_{\max,0}^{b}$** = −β) and a new mutation **C** occurs on the island at a loosely linked locus 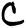. In the absence of epistasis, this mutation can invade and establish if, and only if, γ>m. 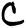 does not affect the barrier at all.

**Figure 3 C:**
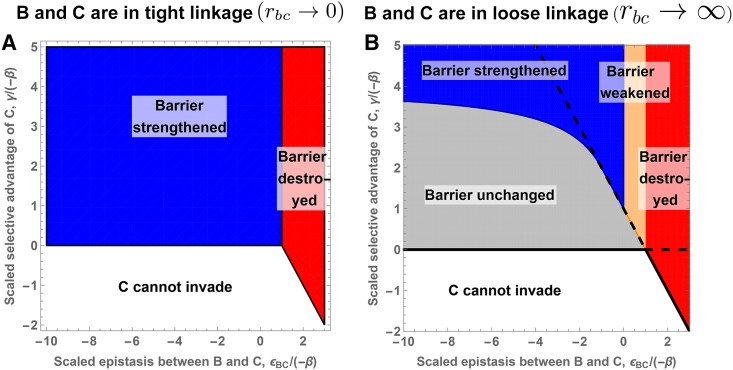
strengthens the genetic barrier formed by a single polymorphic locus: comparison between a new mutation in tight linkage and one in loose linkage. The *x*-axis shows the strength of epistasis between **B** and **C**. The *y*-axis shows the selective advantage of new allele **C**. The background color indicates the consequence of the invasion of allele **C** on the genetic barrier at the 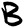 locus. Gray: the genetic barrier remains unchanged; blue: the genetic barrier is strengthened; orange: the genetic barrier is weakened; and red: the polymorphism at locus 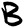 is lost. In addition, on panel B, the solid black line gives the necessary condition for invasion of allele **C** on the island. Below this, bound invasion is always impossible. The black dashed line gives the sufficient condition for invasion. Above this bound, allele **C** can always invade, regardless of the migration rate (provided the polymorphism at the 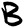 locus still exists). Analytical expressions for the two lines are given in Equation (C2) in File S3.

Figure 3B shows the effect of epistasis between **C** and **B** on the barrier strength. As expected, negative epistasis can strengthen the genetic barrier (blue area), while positive epistasis will almost always weaken it (orange and red areas). However, Figure 3 also shows that negative epistasis is not sufficient to strengthen the barrier. Indeed, a **C** allele may invade for sufficiently weak migration, but will be the first polymorphism swamped once migration increases (gray area). Obviously, in this case, the barrier strength $m_{\max}^{b}$ at the polymorphic 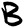 locus remains unaffected.

It is instructive to see how linkage affects the parameter range where further adaptation leads to a stronger barrier. For tight linkage (adaptation at the polymorphic locus itself), any allele with γ>0 will invade the island and will strengthen the barrier (see Figure 3A) as long as epistasis does not cancel the selective disadvantage of allele **B** ($\epsilon_{BC}+\beta >0$). In contrast, strengthening the barrier by adaptation at a loosely linked locus is much more difficult. To reinforce the barrier at the loosely linked 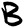 locus, the new **C** allele has to withstand both migration for m> $m_{\max}^{b}$ and the hybrid cost generated by its interaction with allele **B**. The first condition alone implies γ>−β as a necessary condition for a stronger barrier. Indeed, for a given γ>−β, the genetic barrier $m_{\max}^{b}$ is strengthened as long as negative epistasis is not too strong. Stronger epistasis results in larger hybrid cost for **C** and therefore a larger direct effect (larger γ) is needed to compensate for it. For γ>−4β, the barrier is strengthened for any negative epistasis, including a lethal incompatibility.

Finally, even if a new **C** allele would strengthen the barrier (blue area), it is not always able to invade. Invasion of allele **C** requires

$$\gamma >m\left(1+\frac{\epsilon_{BC}}{\beta }\right).$$ (3)

From Equation (3), one can deduce that a necessary condition for invasion of allele **C** is γ>0 and a sufficient one is $\gamma >-\left(\beta +\epsilon_{BC}\right).$ For any γ value between these two limits, invasion will be possible only if migration is sufficiently small. Such a constraint does not exist for the tight linkage case, as migration does not affect the fate of the new allele (given that 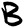 remains polymorphic).

So far, we have considered the interaction of a new island adaptation **C** with a continental adaptation **B**. Alternatively, there are two other possibilities: we can also study interactions between **B** and a new continental adaptation or interactions among two island adaptations. The results are similar (see Figures C2 and C3 in File S3 and our discussion in the Supplemental Material).

#### Extension of a two-locus genetic barrier:

To complete the analysis of this section, we now ask how an existing genetic barrier of two interacting loci in loose linkage is affected by adaptation at a third locus that is also in loose linkage with the previous ones. In particular, we focus on the question of how a continental allele (the **B** allele at the 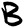 locus) can be prevented from swamping the island. Depending on the direct fitness effect β of this allele on the island, we find similarities or differences to the extension from one to two loci discussed above.

**Figure 4:**
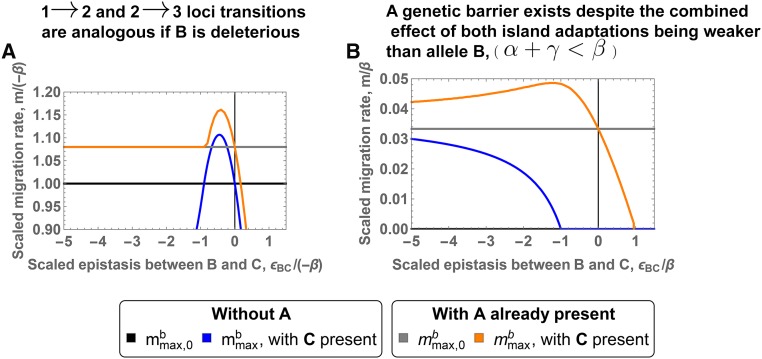
$m_{\max}^{b}$ for two and three loci in loose linkage. The *x*-axis corresponds to the epistasis between alleles **B** and **C**. The *y*-axis measures migration rate. The different lines correspond to $m_{\max}^{b},$ the resistance to swamping at locus 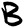 under different scenarios. The initial single-locus and two-locus barriers, $m_{\max,0}^{b}$ ≤ $m_{\max,0}^{Ab},$ are given in black and gray. The impact of a new allele **C** on the single-locus and two-locus genetic barrier is represented by the blue and orange lines, respectively. Allele **B** is deleterious on the island for panel A and advantageous on the island for panel B. The thin vertical black line indicates the absence of epistasis. Panel A is obtained for ($\alpha /-\beta =2,\gamma /-\beta =1.9\;\text{and}\;\epsilon_{AB}/-\beta =-0.2$) and panel B for ($\alpha /\beta =0.2,\gamma /\beta =0.15\;\text{and}\;\epsilon_{AB}/\beta =-3$).

If the continental adaptation **B** is deleterious on the island (β<0), direct selection against migrants (all carrying allele **B**) contributes to the genetic barrier, $m_{\max}^{b},$ at that locus. As Figure 4A shows, transition from two to three loci is analogous to the step from one to two loci and also the qualitative results agree (see section C1.2 in File S3 for details). Indeed, the presence of a first island adaptation (the **A** allele) does not make it any easier for a second, loosely linked island adaptation (the **C** allele) to strengthen the genetic barrier. In particular, the **C** allele still needs to have a stronger direct effect (in magnitude) than **B**, γ>−β. Allele **C** also needs to interact negatively with allele **B**, $\epsilon_{BC}<0.$ Finally, since this interaction generates some hybrid cost, this cost must be compensated by some extra local adaptation (larger γ). For example, a new mutation, interacting with allele **B**, with a direct selective advantage γ slightly larger than −β, might not be able to strengthen the genetic barrier even if it fulfills the first criteria. These conditions are analogous to the one-to-two-locus barrier transition (see Figure 3B, gray area above the −γ/β=1 line). However, **C** can be weaker than the **A** adaptation (γ<α) and still lead to a stronger barrier (see Figure 4A).

We now consider a continental allele **B** that is beneficial also on the island (Figure 4B). In the haploid model, a single-locus genetic barrier is impossible. A genetic barrier can be formed if a second polymorphic locus, 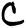, interacts with 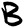 through negative epistasis, generating selection against hybrids. However, a stable genetic barrier only exists if the direct and the epistatic effect of the **C** allele are both strong, $\epsilon_{BC}<-\beta -m$ and γ>4m, (see section C1.1.2 in File S3 for details), represented by the blue line in Figure 4.

Consider now such a two-locus barrier between loci 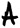 and 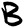. We want to investigate under which conditions a polymorphism at a loosely linked locus 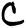 strengthens the barrier against swamping at the 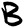 locus, $m_{\max}^{b}$ (orange line on Figure 4). With an **A** allele already present, there is no lower bound for the negative epistasis of the new **C** allele: any value $\epsilon_{BC}$ ($\epsilon_{BC}<0$) can increase the barrier strength. The new allele still has to fulfill a condition on the direct effect: γ> $m_{\max,0}^{b}.$ Otherwise, allele **C** is the first allele that is lost when gene flow increases. However, the condition is weaker than the one on the **A** allele; indeed γ>α/4 is a sufficient condition. Allele **C** can even have the weakest direct effect (Figure 4A) and still contribute to the strengthening of the barrier, in contrast to the case β<0 discussed above. The two island adaptations share the cost of forming hybrids, making it possible to prevent a strongly advantageous continental allele to fix on the island, despite their own relatively weak selective advantage (α+γ<β) (Figure 4A).

Not only the maintenance, but also the invasion, of the new polymorphism in loose linkage is strongly affected by the existence of a polymorphism at locus 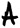. In its absence, the new mutation has to overcome the migration cost and the full incompatibility due to **B** being already fixed $\gamma >m-\epsilon_{BC}.$ In addition, epistasis has an ambiguous effect: it hinders invasion of the new allele while the formation of the two-locus genetic barrier requires relatively strong negative epistasis ($\epsilon_{BC}<-\beta -m$). This ambiguous effect makes invasion and establishment of a genetic barrier in this setting extremely unlikely. However, once a two-locus genetic barrier exists, invasion of a new allele is much easier and always possible if migration is sufficiently small (the invasion criterion tends to γ as m→0). Invasion of a third mutation is therefore similar to the previous case (allele **B** is deleterious on the island).

As we have seen, the constraints on the **C** allele are not so severe, but the flip side is that also the effect on the barrier strength is quite weak: roughly 10% of the direct effect of the **C** allele for Figure 4A and 5% for Figure 4B. In comparison, when all loci are in tight linkage, 100% of the direct effect of the new mutation contributes to strengthening the genetic barrier.

In the supplement, we explore a slightly different scenario, where the new mutation **C** does not interact directly with allele **B** but with allele **a**. Our results show that indirect strengthening of the genetic barrier, by increasing the marginal fitness of the **A** allele, can be the most efficient scenario (Figure C6 in File S3).

To summarize, if **B** is deleterious on the island, the presence of previous island adaptations does not much affect the invasion criterion for a new barrier gene, nor its impact on the barrier strength. However, if **B** is advantageous on the island, previous island adaptations change the invasion criteria from extremely stringent to somewhat more flexible. In addition, a genetic barrier can exist over a larger parameter range thanks to this third polymorphism.

#### Barrier strength and linkage architecture:

Assume now that 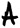, 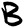, and 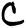 are placed without restrictions on recombination distance. For a given set of selection parameters, which linkage architecture will form the strongest barrier?

For a two-locus genetic barrier (loci 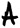 and 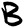), this question has been addressed by Bank *et al.* (2012). The main finding there is that selection against migrants is strongest for tight linkage while selection against hybrids is maximal in loose linkage, when most incompatible hybrids are produced. With both factors acting, the strongest barrier still results from one of these extreme architectures: $m_{\max}^{Ab}$ is maximized for tight linkage whenever selection against migrants is the main driving force. In particular, this is the case whenever **B** is deleterious on the island (Figure C20a in File S3). In contrast, selection against hybrids is the only viable factor if the continental type, **aB**, also has the highest fitness on the island. In this case, we obtain the maximal $m_{\max}^{Ab}$ in loose linkage (Figure C20b in File S3). Assuming that a genetic barrier can be formed both in tight and loose linkage, the loose linkage architecture forms the strongest barrier if:

$$\frac{3}{4}\alpha <\beta <\alpha \;\text{and}\;\epsilon_{AB}<\frac{\alpha \beta }{3\alpha -4\beta }.$$ (4)

In particular, there is never a maximum for intermediate recombination rates.

**Figure 5:**
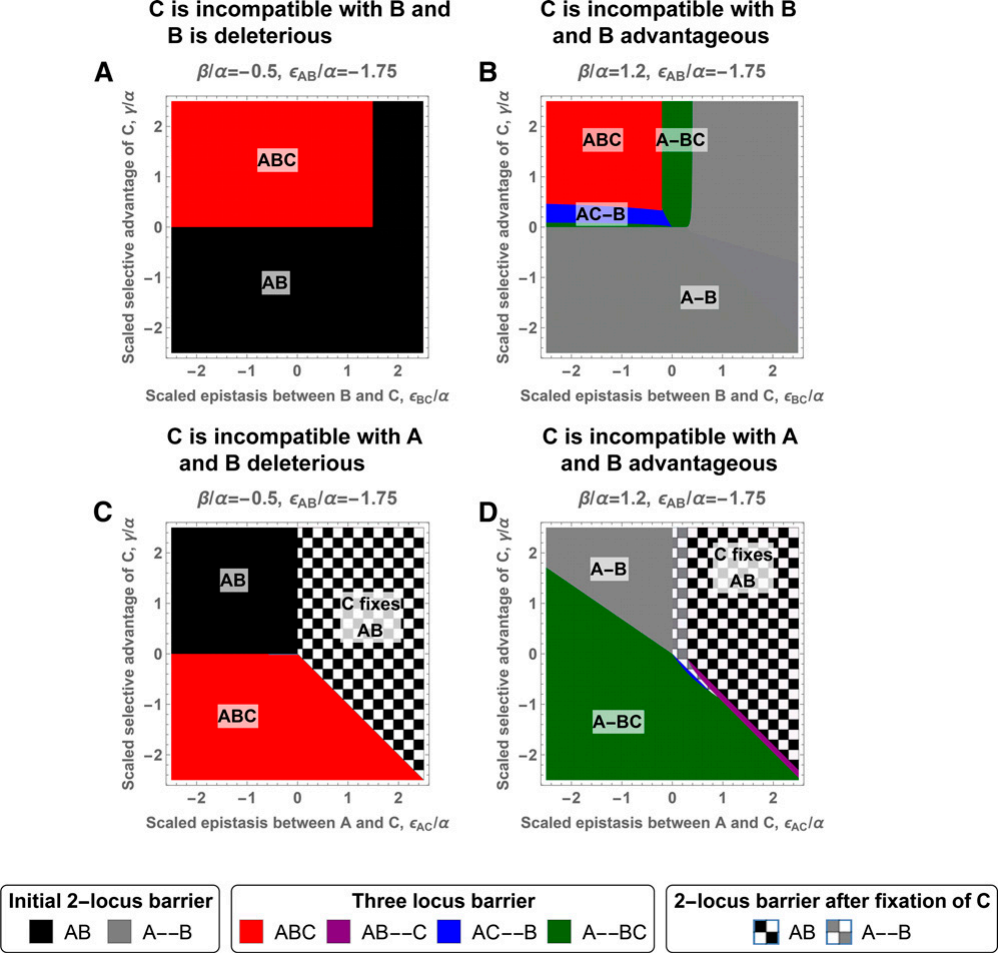
Linkage architecture forming the strongest genetic barrier for three mutations. In each panel, the *x*-axis corresponds to the epistasis between **C** and its interacting allele, **B** for the first row and **A** for the second row. The *y*-axis corresponds to the selective advantage of allele **C** on the island. The different colors indicate the linkage architecture and location at which a **C** mutation should appear to maximize $m_{\max}^{Ab}.$ Having all loci in tight linkage can be interpreted as the existence of a single island of divergence, and two loci in tight linkage and a third one in loose linkage as two islands of divergence, see *Discussion*. The case of three loci in loose linkage is not represented as it never provides the strongest barrier. Analytical expressions for the barrier strengths are given in the Equations (C7)–(C14) in File S3. If the initial barrier remains the strongest when **C** appears on the continent, it indicates that the new mutation will always weaken the barrier. If **C** appears on the island, the barrier is unaffected by the presence of the new mutation. The strongest genetic barrier corresponds to: Initial two-locus barrier: 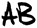, black and 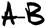, gray. Three-locus barrier: 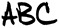, red; 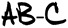, purple; 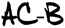, blue; and 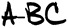, green. Two-locus barrier after fixation of **C**: black and white chequered pattern, 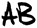 and gray and white chequered pattern, 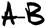.

The case of three loci is more complicated because conflicting options can exist, *e.g.*, the strongest barrier for pairs 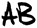 and 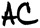 is obtained with the different loci in tight linkage, but the strongest barrier for the pair 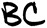 is generated with the two loci in loose linkage. Still, numerical analysis suggests that the strongest barrier is obtained at the extreme ends of the recombination scale, either for r→0 or for r→∞ between pairs of loci (we were not able to prove this claim, but did not find any counterexamples in numerical checks; see in Figures C24 and C25 in File S3). In more detail, we find the following. First, assume that **C** appears on the island. As long as tight linkage among 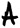 and 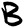 provides the strongest two-locus barrier, 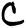 in tight linkage with 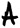 and 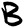 formed the strongest barrier (Figure 5, A and B red area, proof in section C3.2.1 in File S3). Selection against migrants is the key mechanism. If the strongest two-locus barrier is shaped by loci 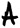 and 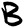 in loose linkage, we obtain the strongest three-locus barrier for an additional adaptation **C** that occurs in tight linkage with either 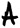 or 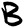. The new mutation contributes to a stronger barrier by either strengthening selection against hybrids (blue area, γ small, $\epsilon_{BC}$ strongly negative), or by strengthening selection against migrants by reducing the direct effect of the **B** allele (green area, $\epsilon_{BC}$ close to 0). Figure 5 shows that the parameter space for having the strongest barrier in tight linkage is much larger than for loose linkage. However, genomic regions around any locus that are effectively in tight linkage are small and randomly placed loci will more likely behave as loosely linked. Therefore, optimal nonlocal barriers with loose linkage between two loci may be easier to evolve than local (island-type) barriers with tight linkage among all loci.

If **C** appears on the continent, we observe similar results (*cf*. Figure 5, C and D). Having all loci in tight linkage forms the strongest barrier as long as the continental adaptations are deleterious on the island and do not generate positive epistasis (see section C3.2.2 in File S3 for proof). If having all loci in tight linkage does not generate the strongest barrier, then two loci in tight linkage and the last one in loose linkage offers the strongest genetic barrier, with 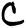 in tight linkage with 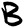, increasing both selection against migrants and hybrids (green area), or 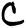 in tight linkage with 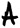, to only strengthen selection against migrants (rare, blue area). Fixing **C** is another possible mechanism to strengthen the genetic barrier if **C** generates positive epistasis with **A** (checkered areas). In this last case, the genomic location of locus 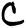 does not matter.

Three loci in loose linkage never seems to be the strongest linkage architecture in our model. Indeed, we did not find such an architecture despite an extensive numerical search (although we were not able to prove this). Results can be different in more complex models. After extending our model to include general three-locus epistasis, we were able to construct a case where the strongest barrier has all three loci in loose linkage (*cf*. Figure C23 in File S3). However, the scenario requires a very specific type of three-locus interaction (epistasis between **B** and **C** is only expressed in the absence of **A**) and careful fine-tuning of the selection parameters.

As a general rule, the strongest barrier is usually formed with two clusters of loci in tight linkage: one cluster formed by all loci with selection against the immigrating allele, and the other cluster with all loci where the immigrating allele is beneficial on the island. These two clusters of loci are in loose linkage with each other, maximizing the expression of the incompatibilities among clusters and thus selection against the immigrating continental alleles that are not themselves deleterious on the island.

#### Extension of a two-locus genetic barrier, diploid populations:

**Figure 6:**
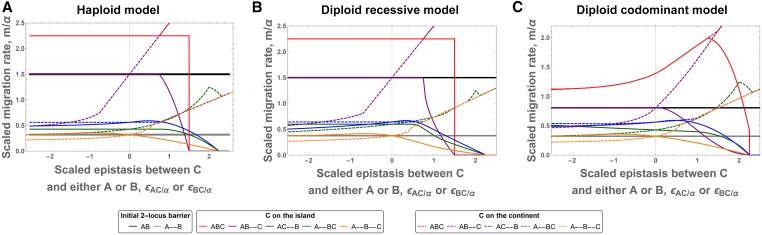
Maximal migration rate, $m_{\max}^{Ab},$ in haploid and diploid models. Colored lines represent the maximal migration rate to maintain a polymorphism at loci 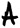 and 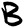 in the presence of a **C** adaptation for various linkage architectures. The black and gray lines serve as reference for a two-locus genetic barrier (in the absence of **C**) for tight linkage and loose linkage, respectively. We observe that the pattern of colored lines in (panel A and panel B) (haploid and diploid recessive) is very similar. For the codominant diploid case (panel C), there are a few notable differences, as explained in the text. Parameters: β/α=−0.5, $\epsilon_{AB}/\alpha =-1.75,$ and γ/α=0.75 if the **C** allele appears on the island (solid lines) and γ/α=−0.75 if **C** appears on the continent (dashed lines). The *x*-axis shows the epistatic interaction between **C** and its interacting allele ($\epsilon_{AC}/\alpha$ if **C** appears on the continent or $\epsilon_{BC}/\alpha$ if **C** appears on the island).

Here, we extend our analysis to diploid populations. More precisely, we are interested in the similarities and differences between the haploid and diploid models. The diploid model is quite complex due to the number of equations and parameters. As mentioned in the model section, we focus on two specific dominance schemes for the interactions: codominance and recessivity. Despite this simplification, only few cases (mostly when the diploid case reduces to the haploid case) allow for analytical results. In Figure 6, we therefore compare numerical results for the strength of migration barriers. Rather than discussing each single linkage architecture (colored lines in Figure 6), we focus on the qualitative similarities and differences that emerge from comparing the total pattern.

Comparing the migration barriers for the haploid case (Figure 6A) with recessive diploids (Figure 6B), we find broad qualitative agreement (if all loci are in tight linkage, $m_{\max}^{Ab}$ for both cases are identical). In particular, adaptation at the 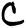 locus will weaken or strengthen the genetic barrier $m_{\max}^{Ab}$ for the same linkage architectures among the three loci, and in approximately the same parameter ranges. Also, having all loci in loose linkage never seems to generate the strongest barrier for a given set of parameters. Furthermore, for a given set of parameters, numerical simulations suggest that we will observe qualitatively the same optimal linkage architectures as in the haploid case when we increase $\epsilon_{BC}$.

Also, the comparison of the haploid case and codominant diploids (Figure 6A *vs.* Figure 6C) shows many similarities. For several architectures, the dynamics (and thus the migration barriers) are identical. Indeed, as long as all interacting loci are in loose linkage, the haploid and diploid codominant model share their dynamics. This result holds for three different linkage architectures: all loci in loose linkage (orange lines), as well as 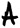 and 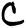 in tight linkage and 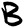 in loose linkage if **C** appears on the island (blue solid line), or 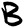 and 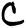 in tight linkage and 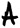 in loose linkage, if **C** appears on the continent (green dashed line). However, there is one major difference: epistasis between loci in tight linkage can be expressed. This is most noticeable when all loci are in tight linkage (red lines). Epistasis can be expressed directly in the F1 generation without any recombination event and therefore the behavior of DMIs in tight linkage differs strongly from its haploid or recessive counterparts. Surprisingly, positive epistasis between **B** and **C**, with **C** appearing on the island, can strengthen the genetic barrier. One can relate this case to the two-locus three-alleles model, where we have seen that reducing the hybrid cost is a viable option to strengthen the genetic barrier. A similar mechanism applies here as well.

## Discussion

How can a genetic barrier build up between two spatially separated populations that are connected by gene flow? What is the relative role of local adaptation and selection against hybrids (incompatibilities) in this process? Starting from a genetically homogeneous ancestral population, the first step of this process requires some amount of local adaptation to protect locally divergent alleles from swamping. For the case of (one or) two additive loci, this was discussed in detail by Bürger and Akerman (2011) and Aeschbacher and Bürger (2014), and for two loci with epistasis (allowing for incompatibilities) and unidirectional gene flow by Bank *et al.* (2012). Here, we have studied in more detail how a genetic barrier can be extended from such a first nucleus. As in Bank *et al.* (2012), we consider the case of unidirectional gene flow from a continental population to an island population.

*A priori*, there is good reason to believe that extending a barrier, once it has been initiated, should be easier than this first step (Navarro and Barton 2003; Bank *et al.* 2012). Indeed, any existing divergence will reduce the effective migration rate (Barton and Bengtsson 1986). This effect is strongest in close linkage to the first divergent locus, but also exists genome-wide, corresponding to what has been called “divergence hitch-hiking” (Via and West 2008) and “genome hitch-hiking” (Feder *et al.* 2012b), respectively. It is primarily this argument that triggered the idea of islands of divergence, which may act as nuclei of emergent speciation [speciation islands, *cf*. Hawthorne and Via (2001), Via and West (2008), Feder and Nosil (2010), and Nadeau *et al.* (2012); for confounding effects due to the sorting of ancestral polymorphisms see Guerrero and Hahn (2017)]. If hybrid incompatibilities are involved in the build-up of such a barrier, there is a second line of argument for a subsequently increased growth of the barrier. This is the so-called snowball effect that predicts the accelerated growth of a genetic barrier between two allopatric populations, (Orr 1995; Orr and Turelli 2001), simply because with more divergent loci, there are more opportunities for incompatibilities between these loci.

Our results shed some light on the probability of observing a snowball effect in parapatry. Generally, strengthening of a genetic barrier in the presence of gene flow, while possible, is not a straightforward process, neither for haploid nor diploid populations. Indeed, although the existence of previous polymorphism can support the establishment of further divergent alleles in some cases, this process is far from being constraint-free. Furthermore, even if a new polymorphism succeeds in establishing, this does not imply that the genetic barrier is strengthened: it can be weakened or destroyed as well.

### Strengthening of an already existing genetic barrier

There are two sources from which a genetic barrier can be built or extended (Bank *et al.* 2012). On the one hand, selection against immigrants is effective as long as there is a fitness deficit for the immigrant haplotype relative to the island haplotype. In this case, selection acts directly to prevent introgression of the continental alleles on the island. On the other hand, selection against hybrids acts against both incompatible alleles (continent and island). It still acts as a force against introgression as long as the proportion of immigrants is small on the island. However, it is less efficient than selection against immigrants as it only acts indirectly through the selection against hybrid descendants. It also is associated with a cost for the island haplotype (production of unfit hybrids).

In the previous literature, diverse approaches have been used to study the accumulation of divergent alleles in incipient species. Flaxman *et al.* (2013, 2014) studied speciation with gene flow in a model without epistasis, purely through the accumulation of genes under local adaptation (selection against immigrants). As the number of locally adapted mutations increases, effective gene flow between both populations gradually declines until it reaches a so-called “congealing” threshold (*sensu* Turner 1967; Barton 1983; Kruuk *et al.* 1999), where effective migration rates are almost zero genome-wide and further divergence can occur at an elevated speed. The model allows for an unlimited number of local adaptation genes and generally leads to very low fitness of immigrants at (or near) speciation. There is no genetic mechanism to induce speciation in this setting: given an environment (such as a laboratory) in which both populations can survive, nothing prevents the production of viable and fertile hybrid offspring. This is at odds with theories of speciation due to the accumulation of genetic incompatibilities. Indeed, studies of allopatric speciation typically focus entirely on incompatibilities (and selection against hybrids after secondary contact) and do not include any local adaptation [*e.g.*, Orr (1995), see also Paixão *et al.* (2014)]. Both mechanisms, selection against immigrants and against hybrids, are included in the two-locus study by Bank *et al.* (2012), which we extend here.

Our results can be summarized as follows: first and foremost, we observe clear differences compared to the allopatric case concerning the accumulation of divergent alleles. Speciation in the presence of gene flow implies that each new barrier gene does not only compete against a single wild-type, but is tested against all haplotypes that can be created by gene flow and recombination. In particular, there is always selection for the reduction of hybrid cost. New adaptations on the island thus need to be locally beneficial to counter two types of costs: the direct “migration cost” to withstand swamping by the corresponding continental allele and (in case of an incompatibility) the hybrid cost. Previous divergence polymorphisms can alleviate the migration cost if a secondary adaptation occurs in close linkage, but not the cost of a stronger incompatibility. As a consequence, the number of potential barrier genes is strongly reduced relative to the allopatric case. Furthermore, there is a high probability for each new successful adaptation, on either the continent or the island, that an existing barrier will be weakened (or even destroyed) rather than strengthened. We have demonstrated this effect going from one to three barrier genes. With an increasing number of divergent genes, the constraints due to hybrid costs should only grow larger, acting against any snowball effect, possibly until some sort of congealing threshold [*sensu* Flaxman *et al.* (2014), Nosil *et al.* (2017)] is reached. Indeed, both the migration pressure and the cost of generating hybrids act as a sieve on potential new barrier genes. Due to this sieve, loci involved in DMIs (under parapatric conditions) should have on average larger direct fitness effects than loci involved in DMIs evolved in allopatry, as they have to compensate for the different costs. Furthermore, the expression of the different incompatibilities makes the process reversible as it is possible to lose some barrier genes if further adaptation reduces the hybrid cost of invading (continental) alleles.

Our results thus show that, in contrast to the allopatric case, reproductive isolation will not evolve with necessity just given enough time and a generic set of substitutions that sometimes lead to incompatibilities. Instead, genetic barriers in the face of gene flow may grow, shrink, or vanish. In particular, growth of the barrier does not only depend on sufficiently weak gene flow, but also on favorable combinations of barrier genes that interact in the right way.

#### Migration may help to build a stronger genetic barrier to swamping:

As explained above (and as expected), it is usually more difficult to extend a barrier when there is ongoing gene flow. However, we have shown that migration is sometimes necessary for a new mutation to invade. Sometimes migration can even promote adaptations (making invasion possible and/or more likely) that strengthen the genetic barrier against swamping. This can happen if the initial genetic barrier can be sustained by selection against migrants only, but the incompatibility is also strong. In that case, a new mutation generating a much weaker incompatibility, but with a weaker direct effect, can invade and strengthen the genetic barrier to gene flow if migration is strong enough. This is analogous to a reinforcement process due to prezygotic incompatibilities that is triggered by migration, (Kirkpatrick and Servedio 1999). In the postzygotic case, any adaptation that strengthens the barrier to swamping by lowering the hybrid cost (*i.e.*, weakening the incompatibility) will make the resulting barrier more dependent on local adaptation and therefore on differences in the environment. Thus, it is questionable whether this is truly a step toward reproductive isolation.

#### The strongest genetic barrier for a specific set of loci:

For a given set of fitness effects (both direct and epistatic) at the barrier loci, we can ask which linkage architecture provides the strongest protection against swamping. In particular: do we get clusters of linked genes for optimal architectures?

For a two-locus barrier, Bank *et al.* (2012) have shown that the most stable architecture is always one with extreme linkage. If the barrier is primarily maintained due to selection against immigrants, tight linkage (r=0) results in the strongest barrier [this is always the case in the absence of epistasis (Akerman and Bürger 2014)]. In contrast, the most stable barrier is obtained with maximally loose linkage (corresponding to r→∞ in the model) if selection mainly acts against hybrid recombinants.

When we extend the two-locus model to three loci, we can distinguish three patterns with extreme linkage among pairs of loci: all three loci in tight linkage, two tightly linked loci and one loosely linked locus, or all three loci loosely linked. We find examples, for each of three patterns mentioned above, where the considered linkage architecture formed the strongest genetic barrier. However, the pattern of three loosely linked loci seems to be very rare. We only observe this result in a custom-made model with three-way epistasis among the three loci and restricted to a small parameter range. Usually, we obtain either one or two islands of divergence: one if continental adaptations are deleterious on the island and two otherwise. As in the two-locus case, we do not observe (based on a limited number of numerical studies) an optimal architecture involving intermediate recombination, even if the genetic barrier is no longer a monotonic function of recombination and local maxima of the barrier strength as a function of the recombination rate exist in some cases (Figure C24b in File S3, blue dashed line). However, note that such an optimum at intermediate recombination distances can occur in stochastic models (Aeschbacher and Bürger 2014).

Although stable architectures will be favored in the presence of gene flow, the most stable barriers are not necessarily the ones that will evolve most easily in natural populations. For most selection parameters, two or more loci in tight linkage provide the strongest barrier. However, the area around each single locus that behaves as essentially tightly linked is usually very small relative to the size of the genome. Thus, if interacting genes are scattered across random positions in the genome, stable configurations will be rare. Chromosomal rearrangements such as inversions can procure larger regions of no recombination, increasing the likelihood of barrier loci in tight linkage. Navarro and Barton (2003) discussed the importance of such rearrangements in the speciation process. However, gene conversion can also occur in inversions (Korunes and Noor 2017). In addition, a study between two *Senecio* species (Brennan *et al.* 2014) found no associations between those rearrangements and incompatible genes.

New adaptations that appear at loosely linked loci could be transient. As demonstrated by Yeaman (2013), adaptations that first occur in different genomic regions can later move into tight linkage due to genome rearrangement. Indeed, some studies, reviewed in Feder *et al.* (2012a), report small regions of divergence hitch-hiking in several species, suggesting that there may be at least a weak trend for an accumulation of divergent sites. However, currently neither theory nor empirical evidence provide a strong basis for divergence islands as a reliable pattern for parapatric speciation.

#### Biological evidence and implications:

Growth of a genetic barrier starting from an initial pairwise DMI could be common in nature. Indeed, Corbett-Detig *et al.* (2013) reported that two locus DMIs already exist within populations of the same species, with an average of 1.15 DMIs between different *Drosophila melanogaster* recombinant inbred lines that were derived from a common parental pool. Segregating incompatibilities have also been found for yeast (Marsit *et al.* 2017). This suggests that speciation through the accumulation of postzygotic incompatibilities may not start from scratch (a common hypothesis in many models) but can rely on divergence that already exists between populations. This makes the process investigated here (how new mutations can strengthen a genetic barrier) a crucial step of the speciation process.

Dettman *et al.* (2008) evolved populations of *Neurospora* in two different environments. They crossed individuals that had evolved independently (in allopatry) either in the same environment (parallel evolution) or in a different environment (divergent evolution). Since crosses between individuals under different selective pressure tended to generate more unfit individuals, they concluded that genes involved in early divergence also generate genetic incompatibilities [see also Kulmuni and Westram (2017)]. This corresponds to the assumptions of our model. In addition, in our model we do not consider independent DMIs but partially saturated ones (one locus involved in two DMIs). Guerrero *et al.* (2017) provided an example of such saturated incompatibilities in the pollen of two species of *Solanum* genus.

Ono *et al.* (2017) measured the epistasis between first-step adaptations within a pathway responsible for fungicide resistance in yeast, with each mutation in a different gene. They found pervasive epistasis among these mutations, with a third of the interactions classified as DMIs. Based on these findings, they suggested a scenario of parallel adaptation (without local adaptation) for allopatric speciation with secondary contact: if populations that adapt in parallel to the same environment (during an allopatric phase) fix different mutations in the same pathway, incompatibilities can easily be generated. Our model predicts that this kind of adaptation (α≈β, *i.e.*, only selection against hybrids) can only be maintained in the face of gene flow when the DMI loci are far away from each other. This is indeed the case for Ono *et al.* (2017): the mutations involved in DMIs occur in genes located on different chromosomes. In addition, our model [and that of Bank *et al.* (2012)] shows that an allopatric phase is not needed for the evolution of such a DMI: it can also evolve with ongoing unidirectional gene flow given that the first substitution happens on the island. For bidirectional gene flow, local adaptation is required. Note that when extended to diploids, the constraint on the linkage architecture vanishes if the incompatibility is expressed in F1 hybrids (codominant incompatibility).

#### Model assumptions and possible extensions:

Our model relies on a number of hypotheses, most of them shared with Bank *et al.* (2012). First, we assume an infinite population size to ignore the effects of genetic drift. This assumption is adequate as long as the population size is large enough that drift can be ignored relative to the other evolutionary forces (1/N≪s,m). We only study whether the mutant can invade or not, but do not consider establishment probabilities. When these are included [see Aeschbacher and Bürger (2014) for two loci without epistasis], the highest establishment probability is often not found for the most stable configuration (tight linkage in this case), but for small, nonzero recombination rates.

We focus entirely on a continent–island model with unidirectional migration. This is realistic if either physical mechanisms enforce unidirectional gene flow (*e.g.*, wind, water current, or flowering time) or if the contribution of both populations to a common migrant pool is strongly biased (because of unequal population size or because of reduced fertility, *e.g.*, of a marginal population). If there is weak back migration, the effects described here should still hold, as long as the island adaptations are not advantageous on the continent. For strong bidirectional migration, generalist genotypes can gain an advantage and different results are obtained (Akerman and Bürger 2014).

Due to the complexity of the system, we restrict our analytical analysis to the limiting cases of recombination (r=0 and r=∞). Bank *et al.* (2012) have shown that the analysis of these limiting cases provides a good understanding of the general case for the two-locus model. Our numerical study for intermediate recombination rates confirms this for three loci (Figures C24 and C25 in File S3). We assume that all loci are autosomal. Höllinger and Hermisson (2017) provide an analysis of a two-locus DMI in parapatry for organelles and sex chromosomes.

Finally, we have restricted our detailed analysis in this paper to epistasis schemes with only pairwise interactions. Complex epistasis networks with interactions linking three or more loci offer further routes to strengthen a genetic barrier that will be explored in a forthcoming study.

## Supplementary Material

Supplemental material is available online at www.genetics.org/lookup/suppl/doi:10.1534/genetics.117.300652/-/DC1.
