# Supplementary material for: The Limits to Parapatric Speciation II: Strengthening a Preexisting Genetic Barrier to Gene Flow in Parapatry
Source: Genetics. 2018 Feb 28;209(1):241–54. doi: 10.1534/genetics.117.300652 (PMC5937195; doi:10.1534/genetics.117.300652)
Supplement: Supplementary file 1 [file 241FileS1.pdf]

Supplementary File A: Detailed model

Alexandre Blanckaert *[1,2] and Joachim Hermisson[1,3]

[1]Department of Mathematics, University of Vienna, 1090 Vienna, Austria

[2]Instituto Gulbenkian de Ciência, 2780-156 Oeiras, Portugal

[3]Mathematics and Biosciences Group, Max F. Perutz Laboratories, 1030 Vienna, Austria

February 28, 2018

A 1 Haploid populations; arbitrary recombination

We consider two panmictic populations with unidirectional migration from one to the other, i.e. a continent-island model. We only focus on the dynamics of the different alleles on the island. This is based on the model used by Bank et al. [2012] and is extended to three diallelic loci, A,B and C. Using the fitness table A1, one can derive, in continuous time, the dynamics of the frequencies of each of the 8 possible haplotypes. The general formulation of the model, for haploid populations, with arbitrary recombination rate is given by equation (A2), with the mean fitness of the population, $\bar{w}$, given in equation (A1). For equation (A2), we assume that the B locus is situated between the A and C loci on the chromosome. To allow for a more general formulation, we define two migration rates: m_I and m_C that are specific to the possible immigrating haplotypes x_3 and x_7: if **C** appears on the island, the immigrating haplotype is x_3 and therefore $m_I = m$ and $m_C = 0$. If **C** appears on the continent, the immigrating haplotype is x_7 and therefore $m_I = 0$ and $m_C = m$.

Hap.	**abc**	**Abc**	**aBc**	**abC**	**ABc**	**AbC**	**aBC**	**ABC**
x_i	x_1	x_2	x_3	x_4	x_5	x_6	x_7	x_8
w_i	0	α	β	γ	$\alpha+\beta$ $+\epsilon_{AB}$	$\alpha+\gamma$ $+\epsilon_{AC}$	$\beta+\gamma$ $+\epsilon_{BC}$	$\alpha+\beta+\gamma$ $+\epsilon_{AB}+\epsilon_{BC}+\epsilon_{AC}$

Table A1: **Frequencies x_i and fitness values w_i of the different haplotypes for haploid populations**

*ablanckaert@igc.gulbenkian.pt

$$\bar{w} = \sum_{i=1}^{8} w_i x_i \tag{A1}$$

$$\left\{\begin{array}{ll} \dot{x_1} = & x_1(w_1 - \bar{w} - m) - r_{ab}D_{12,A} - r_{bc}D_{14,C} \\ \dot{x_2} = & x_2(w_2 - \bar{w} - m) - r_{ab}D_{21,a} - r_{bc}D_{26,C} \\ \dot{x_3} = & m_I + x_3(w_3 - \bar{w} - m) - r_{ab}D_{35,A} - r_{bc}D_{37,C} \\ \dot{x_4} = & x_4(w_4 - \bar{w} - m) - r_{ab}D_{46,A} - r_{bc}D_{41,c} \\ \dot{x_5} = & x_5(w_5 - \bar{w} - m) - r_{ab}D_{53,a} - r_{bc}D_{58,C} \\ \dot{x_6} = & x_6(w_6 - \bar{w} - m) - r_{ab}D_{64,a} - r_{bc}D_{62,c} \\ \dot{x_7} = & m_C + x_7(w_7 - \bar{w} - m) - r_{ab}D_{78,A} - r_{bc}D_{73,c} \\ \dot{x_8} = & x_8(w_8 - \bar{w} - m) - r_{ab}D_{87,a} - r_{bc}D_{85,c} \end{array}\right. \quad \text{with} \quad \left\{\begin{array}{ll} D_{ij,X} = & x_i p_X - x_j(1 - p_X) \\ p_A = & (1 - p_a) = x_2 + x_5 + x_6 + x_8 \\ p_B = & (1 - p_b) = x_3 + x_5 + x_7 + x_8 \\ p_C = & (1 - p_c) = x_4 + x_6 + x_7 + x_8 \end{array}\right. \tag{A2}$$

By making the expression for the linkage disequilibrium term in equation (A2) explicit, we provide a detailed expression of each term, emphasizing which element of the equation corresponds to which evolutionary force:

$$\begin{aligned} \dot{x_1} = \; & \underbrace{x_1(w_1 - \bar{w})}_{\text{(soft) selection}} - \underbrace{x_1 * m}_{\text{individuals replaced by migrants}} \\ & \underbrace{- r_{ab}(\underbrace{x_1 p_A}_{\text{destruction of haplotype } \mathbf{abc}} - \underbrace{x_2(1 - p_A)}_{\text{formation of haplotype } \mathbf{abc}})}_{\text{recombination between loci A and B}} \\ & \underbrace{- r_{bc}(\underbrace{x_1 p_C}_{\text{destruction of haplotype } \mathbf{abc}} - \underbrace{x_4(1 - p_C)}_{\text{formation of haplotype } \mathbf{abc}})}_{\text{recombination between loci B and C}} \end{aligned} \tag{A3}$$

The complete system can also be expressed in terms of allele frequencies and linkage disequilibrium between loci instead of haplotype frequencies. Equation (A4) gives the relation between these two ways of expressing the system of equations and equation (A5) gives the resulting system once expressed with the new variables, if **C** appears on the island ($\epsilon_{AC} = 0, m_I = m, m_C = 0$). Equation (A5) is especially useful when we assume loose linkage, since it simplifies to equation (1), presented in the main manuscript.

$$\begin{cases} p_A &= x_2 + x_5 + x_6 + x_8 \\ p_B &= x_3 + x_5 + x_7 + x_8 \\ p_C &= x_4 + x_6 + x_7 + x_8 \\ D_{AB} &= (x_5 + x_8)(x_1 + x_4) - (x_2 + x_6)(x_3 + x_7) \\ D_{BC} &= (x_7 + x_8)(x_1 + x_2) - (x_4 + x_6)(x_3 + x_5) \\ D_{AC} &= (x_6 + x_8)(x_1 + x_3) - (x_2 + x_5)(x_4 + x_7) \\ D_{ABC} &= x_8 - p_A D_{BC} - p_B D_{AC} - p_C D_{AB} - p_A p_B p_C \end{cases} \tag{A4}$$

$$
\begin{cases}
\dot{p_A} = p_A(-m + (1-p_A)\alpha - (D_{AB} + (-1+p_A)p_B)\epsilon_{ab}) + \epsilon_{bc}(D_{ABC} + p_B D_{AC} + p_C D_{AB}) \\
\quad + D_{AC}\gamma + D_{AB}(\beta + \epsilon_{AB}) \\
\dot{p_B} = m + p_B(-m\beta - p_B\beta - p_A(-1+p_B)\epsilon_{AB} - (D_{BC} + (-1+p_B)p_C)\epsilon_{BC}) \\
\quad + D_{AB}(\alpha - (-1+p_B)\epsilon_{AB}) + D_{BC}(\gamma + \epsilon_{BC}) \\
\dot{p_C} = p_C(-m + (1-p_C)\gamma - (D_{BC} - (1-p_C)p_B)\epsilon_{BC}) + \epsilon_{AB}(D_{ABC} + p_B D_{AC} + p_A D_{BC}) \\
\quad + D_{AC}\alpha + D_{BC}(\beta + \epsilon_{BC}) \\
\dot{D_{AB}} = m p_A(-1+p_B) + D_{ABC}\gamma - D_{AB}^2\epsilon_{AB} - p_A p_B(-1 + p_A + p_B - p_A p_B)\epsilon_{AB} \\
\quad + D_{AB}(-m - r_{ab} + \alpha(1-2p_A) + \beta(1-2p_B) - (-1+p_A+p_B)\epsilon_{AB} \\
\quad -(D_{BC} + (-1+2p_B)p_C)\epsilon_{BC}) - (-1+p_B)(D_{ABC} + D_{AC}p_B)\epsilon_{BC} \\
\dot{D_{BC}} = m(-1+p_B)p_C + D_{ABC}(\alpha + \epsilon_{AB}) - (D_{ABC} + D_{AC}(-1+p_B))p_B\epsilon_{AB} - D_{BC}^2\epsilon_{BC} \\
\quad + (-1+p_B)p_B(-1+p_C)p_C\epsilon_{BC} + D_{BC}(-m - r_{bc} + \beta - 2p_B\beta + \gamma - 2p_C\gamma \\
\quad -(D_{AB} + p_A(-1+2p_B))\epsilon_{AB} - (-1+p_B+p_C)\epsilon_{BC}) \\
\dot{D_{AC}} = m p_A p_C + D_{ABC}(\beta + \epsilon_{AB}) - (D_{ABC} + D_{BC}(-1+p_A))p_A\epsilon_{AB} \\
\quad -(-1+p_C)(D_{ABC} + D_{AB}p_C)\epsilon_{BC} - D_{AC}(m + r_{ab} + r_{bc} + (-1+2p_A)\alpha \\
\quad + (-1+2p_C)\gamma + D_{AB}\epsilon_{AB} + D_{BC}\epsilon_{BC} + 2p_B p_C\epsilon_{BC} + p_B(-\epsilon_{AB} + 2p_A\epsilon_{AB} - \epsilon_{BC})) \\
\dot{D_{ABC}} = D_{BC} m p_A + D_{AB} m p_C + m p_A p_C - m p_A p_B p_C - 2D_{AB}D_{BC}\beta - D_{AB}D_{BC}\epsilon_{AB} \\
\quad + D_{BC}p_A\epsilon_{AB} - D_{BC}p_A^2\epsilon_{AB} - 2D_{BC}p_A p_B\epsilon_{AB} + 2D_{BC}p_A^2 p_B\epsilon_{AB} - D_{AB}(D_{BC} \\
\quad + p_C(-1 - 2p_B(-1+p_C) + p_C))\epsilon_{BC} + D_{ABC}(-m - r_{ab} - r_{bc} + \alpha - 2p_A\alpha + \beta \\
\quad -2p_B\beta + \gamma - 2p_C\gamma - (-1 + 2D_{AB} + p_A + p_B)\epsilon_{AB} - (-1 + 2D_{BC} + p_B)\epsilon_{BC} \\
\quad -p_C\epsilon_{BC}) + D_{AC}(-2D_{AB}\alpha - 2D_{BC}\gamma - D_{AB}\epsilon_{AB} - D_{BC}\epsilon_{BC} + (-1+p_B)(m \\
\quad + p_B(-\epsilon_{AB} + 2p_A\epsilon_{AB} - \epsilon_{BC} + 2p_C\epsilon_{BC})))
\end{cases}
\tag{A5}
$$

It is not possible to solve the system (A2) or (A5) for arbitrary recombination rates. Therefore, we mainly focus on limiting cases, tight linkage or loose linkage ($r_{ab} \to 0$ or ∞, and $r_{bc} \to 0$ or ∞). Below, we detail the different systems of equations corresponding specifically to each linkage architecture, both for haploid and diploid populations.

A 2 Haploid populations; specific linkage architectures

For tight linkage or loose linkage among the three loci, the full system of equations reduces to a slightly simpler system. The equations for the different linkage architectures can be derived from (A2) and (A5), either assuming vanishing recombination $r_{ab} = 0$, $r_{bc} = 0$, or no linkage disequilibrium $D_{AB} = 0, D_{BC} = 0, D_{AC} = 0$ and $D_{ABC} = 0$. For equation (A7), loci B and C need to be exchanged in the system (A5) to account for the architecture **AC-B** with the B locus flanking the AC pair.

- ABC: If all loci are in tight linkage, the system is given by equation (A6). If **C** appears on the island, $\{\underset{i\neq 3}{M_i} = 0, M_3 = m\}$, else $\{\underset{i\neq 7}{M_i} = 0, M_7 = m\}$. $\bar{w}$ corresponds to the Malthusian fitness, defined in equation (A1).

$$\dot{x_i} = x_i(w_i - \bar{w} - m) + M_i \tag{A6}$$

- AC-B: If the A and B loci are in loose linkage and the C locus appears in tight linkage with the A locus, then the dynamics of such a system are described in equations (A7) with $m_C = 0$ if **C** appears on the island and $m_C = m$ otherwise.

$$\begin{cases} \dot{p_{Ac}} &= p_{Ac}(-m - (-1 + p_{Ac} + p_{AC})\alpha - p_{aC}\gamma - p_{AC}\gamma - (-1 + p_{Ac})p_B\epsilon_{AB} \\ &- p_{AC}p_B\epsilon_{AB} - p_{AC}\epsilon_{AC} - (p_{aC} + p_{AC})p_B\epsilon_{BC}) \\ \dot{p_{aC}} &= m_C + p_{aC}(-m + \gamma - p_{aC}\gamma + p_{Ac}(-\alpha - p_B\epsilon_{AB}) \\ &+ p_{AC}(-\alpha - \gamma - p_B\epsilon_{AB} - \epsilon_{AC}) - (-1 + p_{aC} + p_{AC})p_B\epsilon_{BC}) \\ \dot{p_{AC}} &= -p_{AC}(m + (-1 + p_{Ac} + p_{AC})\alpha + (-1 + p_{aC} + p_{AC})\gamma \\ &+ (-1 + p_{Ac} + p_{AC})p_B\epsilon_{AB} - \epsilon_{AC} + p_{AC}\epsilon_{AC}) - p_{AC}(-1 + p_{aC} + p_{AC})p_B\epsilon_{BC} \\ \dot{p_B} &= -(-1 + p_B)(m + p_B(\beta + (p_{Ac} + p_{AC})\epsilon_{AB} + (p_{aC} + p_{AC})\epsilon_{BC})) \end{cases} \tag{A7}$$

- A-BC: If the A and B loci are in loose linkage and the C locus appears in tight linkage with the B locus, then the dynamics of this system are given in equation (A8) with $m_I = m, m_C = 0$ if **C** appears on the island and $m_I = 0, m_C = m$ otherwise.

$$
\begin{cases}
\dot{p_{Bc}} &= m_I + p_{Bc}(-m - (-1 + p_{Bc} + p_{BC})\beta - p_A(-1 + p_{Bc})\epsilon_{AB} \\
&+ \; p_{bC}(-\gamma - p_A\epsilon_{AC}) + p_{BC}(-\gamma - p_A(\epsilon_{AB} + \epsilon_{AC}) - \epsilon_{BC})) \\
\dot{p_{bC}} &= p_{bC}(-m + \gamma - p_{bC}\gamma + p_{Bc}(-\beta - p_A\epsilon_{AB}) - p_A(-1 + p_{bC})\epsilon_{AC} \\
&+ \; p_{BC}(-\beta - \gamma - p_A(\epsilon_{AB} + \epsilon_{AC}) - \epsilon_{BC})) \\
\dot{p_{BC}} &= m_C - p_{BC}(m + (-1 + p_{Bc} + p_{BC})\beta + (-1 + p_{bC} + p_{BC})\gamma \\
&+ \; p_A(-1 + p_{Bc} + p_{BC})\epsilon_{AB} + p_A(-1 + p_{bC} + p_{BC})\epsilon_{AC} - \epsilon_{BC} + p_{BC}\epsilon_{BC}) \\
\dot{p_A} &= -p_A(m + (-1 + p_A)(\alpha + (p_{Bc} + p_{BC})\epsilon_{AB} + (p_{bC} + p_{BC})\epsilon_{AC}))
\end{cases}
\tag{A8}
$$

- **AB-C**: The system of equations for having the **A** and **B** loci in tight linkage and the **C** loci in loose linkage is equivalent to the **AC-B** architecture if **C** appears on the continent. One needs to simply exchange the role of the **B** and **C** loci. In that case, equation (A7) can be used with the following transformations:

$$\beta \leftrightarrow \gamma, \epsilon_{AB} \leftrightarrow \epsilon_{AC}, p_{Ac} \rightarrow p_{Ab}, p_{AC} \rightarrow p_{AB}, p_{aC} \rightarrow p_{aB}, p_B \rightarrow p_C. \tag{A9}$$

  If **C** appears on the island, then the dynamics of this system are equivalent to those of the **A-BC** architecture, if the role of the **A** and **C** loci are switched. In that case, equation (A8) can be used with the following transformations

$$\alpha \leftrightarrow \gamma, \epsilon_{AB} \leftrightarrow \epsilon_{BC}, p_{Bc} \rightarrow p_{aB}, p_{BC} \rightarrow p_{AB}, p_{bC} \rightarrow p_{Ab}, p_A \rightarrow p_C \tag{A10}$$

- **A-B-C**: All loci are in loose linkage. The system of equations is given in the main text, see equation (1).

In addition to $r \rightarrow 0$, we can also study the case $r = 0$. In that case, it corresponds to a new allele at an already existing polymorphism. The equations for the case $r = 0$, instead of $r \rightarrow 0$ can be easily deduced by removing the unwanted allele from the system of equation; for example for equation (A8), by having $p_{BC} = 0$ or $p_{bC} = 0$. The system reduces from a system of 4 equations to a system of three equations.

A 3 Diploid populations

For diploid populations, one needs to define the level of dominance both of the single locus effect (or direct effect) and of the different epistatic interactions. We assume additivity for the direct effect of mutations. However, epistatic interactions can have different levels of dominance as long as the symmetry is respected (e.g., **AaBB** and **AABb** generate the same amount of epistasis); the full fitness table for diploids (only pairwise epistasis) is given below (Table A2).

w_{ij}	abc $w_{1j} =$	Abc $w_{2j} =$	aBc $w_{3j} =$	abC $w_{4j} =$	ABc $w_{5j} =$	AbC $w_{6j} =$	aBC $w_{7j} =$	ABC $w_{8j} =$
abc $w_{i1} =$	0	α	β	γ	$\alpha+\beta+\eta_{AB}$	$\alpha+\gamma+\eta_{AC}$	$\beta+\gamma+\eta_{BC}$	$\alpha+\beta+\gamma$ $+\eta_{AB}+\eta_{BC}+\eta_{AC}$
Abc $w_{i2} =$		2α	$\alpha+\beta$ $+\eta_{AB}$	$\alpha+\gamma$ $+\eta_{AC}$	$2\alpha+\beta+\epsilon_{AB}$	$2\alpha+\gamma+\epsilon_{AC}$	$\alpha+\beta+\gamma$ $+\eta_{AB}+\eta_{BC}+\eta_{AC}$	$2\alpha+\beta+\gamma$ $+\eta_{BC}+\epsilon_{AB}+\epsilon_{AC}$
aBc $w_{i3} =$			2β	$\beta+\gamma$ $+\eta_{BC}$	$\alpha+2\beta$ $+\epsilon_{AB}$	$\alpha+\beta+\gamma$ $+\eta_{AB}+\eta_{BC}+\eta_{AC}$	$2\beta+\gamma+\epsilon_{BC}$	$\alpha+2\beta+\gamma$ $+\eta_{AC}+\epsilon_{AB}+\epsilon_{BC}$
abC $w_{i4} =$				2γ	$\alpha+\beta+\gamma$ $+\eta_{AB}+\eta_{BC}+\eta_{AC}$	$\alpha+2\gamma+\epsilon_{AC}$	$\beta+2\gamma+\epsilon_{AC}$	$\alpha+\beta+2\gamma$ $+\eta_{AB}+\epsilon_{BC}+\epsilon_{AC}$
ABc $w_{i5} =$					$2\alpha+2\beta+\theta_{AB}$	$2\alpha+\beta+\gamma$ $+\eta_{BC}+\epsilon_{AB}+\epsilon_{AC}$	$\alpha+2\beta+\gamma$ $+\eta_{AC}+\epsilon_{AB}+\epsilon_{BC}$	$2\alpha+2\beta+\gamma$ $+\epsilon_{BC}+\epsilon_{AC}+\theta_{AB}$
AbC $w_{i6} =$						$2\alpha+2\gamma+\theta_{AC}$	$\alpha+\beta+2\gamma$ $+\eta_{AB}+\epsilon_{BC}+\epsilon_{AC}$	$2\alpha+\beta+2\gamma$ $+\epsilon_{AB}+\epsilon_{BC}+\theta_{AC}$
aBC $w_{i7} =$							$2\beta+2\gamma+\theta_{BC}$	$\alpha+2\beta+2\gamma$ $+\epsilon_{AB}+\epsilon_{AC}+\theta_{BC}$
ABC $w_{i8} =$								$2\alpha+2\beta+2\gamma$ $+\theta_{AB}+\theta_{BC}+\theta_{AC}$

Table A2: **Fitness values (w_{ij}) of the different genotypes for diploid individuals** We give the fitness of each genotype. Since we assume that all epistatic interaction are symmetric, we have $w_{ij} = w_{ji}$. Therefore, we only gives the fitness values of the genotypes for $i \leq j$

The marginal fitness w_i for each haplotype is $w_i = \sum_{j=1}^{8} x_j w_{ij}$. The mean fitness is given as the weighted mean of the marginal fitness: $\bar{w} = \sum_{i=1}^{8} x_i \sum_{j=1}^{8} x_j w_{ij}$. Below, we give the system of equations for each of the linkage architectures mentioned in the previous section for haploid populations.

- **ABC**: If all loci are in tight linkage, the system of equations is equivalent to the haploid case with all loci in tight linkage, given in equation (A6), with w_i standing for the marginal fitness of the i haplotype.

- **AC-B**: If the **A** and **B** loci are in loose linkage and the **C** locus appears in tight linkage with the **A** locus, then the dynamics of such a system are described in equations (A11) with $m_C = 0$ if **C** appears on the island and $m_C = m$ otherwise. One can note that equation (A11) reduces to equation (A7) if the incompatibilities are all codominant, *i.e.* $\eta_{AB} = \frac{\epsilon_{AB}}{2}$ and $\theta_{AB} = 2\epsilon_{AB}$ and **C** appears on the island.

$$
\begin{cases}
\dot{p_{Ac}} &= p_{Ac}\left(4p_B^2\epsilon_{BC}p_{aC}^2 - 2p_B\epsilon_{BC}p_{aC}^2 - 4p_B^2\eta_{BC}p_{aC}^2 + 4p_B\eta_{BC}p_{aC}^2 - p_B^2\theta_{BC}p_{aC}^2 - \gamma p_{aC} - p_B^2\epsilon_{BC}p_{aC}\right.\\
&- 2p_{Ac}\eta_{AC}p_{aC} + \eta_{AC}p_{aC} + 2p_B^2\eta_{BC}p_{aC} - 2p_B\eta_{BC}p_{aC} - m - (p_{Ac}+p_{AC}-1)\alpha + 4p_{Ac}^2p_B^2\epsilon_{AB}\\
&- 5p_{Ac}p_B^2\epsilon_{AB} + p_B^2\epsilon_{AB} - 2(p_{Ac}-1)p_{Ac}p_B\epsilon_{AB} - 4p_{Ac}^2p_B^2\eta_{AB} + 6p_{Ac}p_B^2\eta_{AB} - 2p_B^2\eta_{AB} + 4p_{Ac}^2p_B\eta_{AB}\\
&- 6p_{Ac}p_B\eta_{AB} + 2p_B\eta_{AB} - p_{Ac}^2p_B^2\theta_{AB} + p_{Ac}p_B^2\theta_{AB} - p_{AC}\left((-8p_{Ac}\epsilon_{AB} + 5\epsilon_{AB} - 8p_{aC}\epsilon_{BC} + \epsilon_{BC} + 8p_{Ac}\eta_{AB}\right.\\
&- 6\eta_{AB} + 8p_{aC}\eta_{BC} - 2\eta_{BC} + 2p_{Ac}\theta_{AB} - \theta_{AB} + 2p_{aC}\theta_{BC})p_B^2 + 2(2p_{Ac}\epsilon_{AB} - \epsilon_{AB} + 2p_{aC}\epsilon_{BC} - 4p_{Ac}\eta_{AB} + 3\eta_{AB}\\
&- 4p_{aC}\eta_{BC} + \eta_{BC})p_B + \gamma + (2p_{aC}+2p_{Ac}-1)\epsilon_{AC} - 2(p_{aC}+p_{Ac}-1)\eta_{AC}) + p_{AC}^2(2\eta_{AC} - \theta_{AC}\\
&+ \left.\left. p_B((4p_B-2)\epsilon_{AB} + (4p_B-2)\epsilon_{BC} + 4(\eta_{AB}+\eta_{BC}) - p_B(4\eta_{AB}+4\eta_{BC}+\theta_{AB}+\theta_{BC}))\right)\right)\\
\dot{p_{aC}} &= m_C + p_{aC}\left(p_B((4p_B-2)\epsilon_{AB} + 4\eta_{AB} - p_B(4\eta_{AB}+\theta_{AB}))p_{Ac}^2 - (\alpha + p_B(2\eta_{AB} + p_B(\epsilon_{AB}-2\eta_{AB}))\right.\\
&+ (2p_{aC}-1)\eta_{AC} + 2p_{AC}(\epsilon_{AC} - \eta_{AC} + p_B((2-4p_B)\epsilon_{AB} + 4(p_B-1)\eta_{AB} + p_B\theta_{AB})))p_{Ac} - m\\
&- p_{AC}\left((\epsilon_{AB} - 8p_{aC}\epsilon_{BC} + 5\epsilon_{BC} - 2\eta_{AB} + 8p_{aC}\eta_{BC} - 6\eta_{BC} + 2p_{aC}\theta_{BC} - \theta_{BC})p_B^2 + 2((2p_{aC}-1)\epsilon_{BC} + \eta_{AB}\right.\\
&- 4p_{aC}\eta_{BC} + 3\eta_{BC})p_B + \alpha + \gamma + 2p_{aC}\epsilon_{AC} - \epsilon_{AC} - 2p_{aC}\eta_{AC} + 2\eta_{AC}) + p_{AC}^2(2\eta_{AC} - \theta_{AC} + p_B((4p_B-2)\epsilon_{AB}\\
&+ (4p_B-2)\epsilon_{BC} + 4(\eta_{AB}+\eta_{BC}) - p_B(4\eta_{AB}+4\eta_{BC}+\theta_{AB}+\theta_{BC}))) - (p_{aC}-1)(\gamma + p_B(p_B\epsilon_{BC} - 2p_B\eta_{BC}\\
&+ \left.\left. 2\eta_{BC} + p_{aC}((2-4p_B)\epsilon_{BC} + 4(p_B-1)\eta_{BC} + p_B\theta_{BC}))\right)\right)\\
\dot{p_{AC}} &= -p_{AC}\left(-4p_B^2\epsilon_{BC}p_{aC}^2 + 2p_B\epsilon_{BC}p_{aC}^2 + 4p_B^2\eta_{BC}p_{aC}^2 - 4p_B\eta_{BC}p_{aC}^2 - \epsilon_{AC}p_{aC} + 5p_B^2\epsilon_{BC}p_{aC} - 2p_B\epsilon_{BC}p_{aC}\right.\\
&+ 2p_{Ac}\eta_{AC}p_{aC} + \eta_{AC}p_{aC} - 6p_B^2\eta_{BC}p_{aC} + 6p_B\eta_{BC}p_{aC} + (p_{aC}-1)p_B^2\theta_{BC}p_{aC} + m + (p_{Ac}+p_{AC}-1)\alpha\\
&+ (p_{aC}-1)\gamma - 4p_{Ac}^2p_B^2\epsilon_{AB} + 5p_{Ac}p_B^2\epsilon_{AB} - p_B^2\epsilon_{AB} + 2(p_{Ac}-1)p_{Ac}p_B\epsilon_{AB} - p_{Ac}\epsilon_{AC} - p_B^2\epsilon_{BC} + 4p_{Ac}^2p_B^2\eta_{AB}\\
&- 6p_{Ac}p_B^2\eta_{AB} + 2p_B^2\eta_{AB} - 4p_{Ac}^2p_B\eta_{AB} + 6p_{Ac}p_B\eta_{AB} - 2p_B\eta_{AB} + p_{Ac}\eta_{AC} - \eta_{AC} + 2p_B^2\eta_{BC} - 2p_B\eta_{BC}\\
&+ p_{Ac}^2p_B^2\theta_{AB} - p_{Ac}p_B^2\theta_{AB} + p_{AC}^2\left((-4\epsilon_{AB} - 4\epsilon_{BC} + 4\eta_{AB} + 4\eta_{BC} + \theta_{AB} + \theta_{BC})p_B^2 + 2(\epsilon_{AB} + \epsilon_{BC}\right.\\
&- 2(\eta_{AB}+\eta_{BC}))p_B - 2\eta_{AC} + \theta_{AC}) + p_{AC}(-((8p_{Ac}-5)\epsilon_{AB} + (8p_{aC}-5)\epsilon_{BC} + 6\eta_{AB} + 6\eta_{BC} + \theta_{AB}\\
&- 2p_{Ac}(4\eta_{AB}+\theta_{AB}) + \theta_{BC} - 2p_{aC}(4\eta_{BC}+\theta_{BC}))p_B^2 + (4p_{Ac}\epsilon_{AB} - 2\epsilon_{AB} + 4p_{aC}\epsilon_{BC} - 2\epsilon_{BC} - 8p_{Ac}\eta_{AB}\\
&- \left. 8p_{aC}\eta_{BC} + 6(\eta_{AB}+\eta_{BC}))p_B + \gamma + 2(p_{aC}+p_{Ac})(\epsilon_{AC}-\eta_{AC}) + 3\eta_{AC} - \theta_{AC})\right)\\
\dot{p_B} &= m - p_B\left(m + (p_B-1)\left((-4p_B\epsilon_{AB} + \epsilon_{AB} + (4p_B-2)\eta_{AB} + p_B\theta_{AB})p_{Ac}^2 + 2(\eta_{AB} + p_B(\epsilon_{AB}-2\eta_{AB})\right.\right.\\
&+ p_{AC}(-4p_B\epsilon_{AB} + \epsilon_{AB} + 4p_B\eta_{AB} - 2\eta_{AB} + p_B\theta_{AB}))p_{Ac} + \beta + p_{AC}^2(-4p_B\epsilon_{AB} + \epsilon_{AB} + \epsilon_{BC} - 2(\eta_{AB}+\eta_{BC})\\
&+ p_B(-4\epsilon_{BC} + 4\eta_{AB} + 4\eta_{BC} + \theta_{AB} + \theta_{BC})) + 2p_{AC}(\eta_{AB} + \eta_{BC} + p_{aC}(\epsilon_{BC} - 2\eta_{BC}) + p_B(\epsilon_{AB} - 4p_{aC}\epsilon_{BC} + \epsilon_{BC}\\
&+ 4p_{aC}\eta_{BC} - 2(\eta_{AB}+\eta_{BC}) + p_{aC}\theta_{BC})) + p_{aC}(2(\eta_{BC} + p_B(\epsilon_{BC}-2\eta_{BC})) + p_{aC}(-4p_B\epsilon_{BC} + \epsilon_{BC} + 4p_B\eta_{BC}\\
&- \left.\left. 2\eta_{BC} + p_B\theta_{BC}))\right)\right)
\end{cases}
\tag{A11}
$$

- **A-BC**: If the **A** and **B** loci are in loose linkage and the **C** locus appears in tight linkage with the **B** locus, then the dynamics of such a system are described in equations (A12) with $m_I = m, m_C = 0$ if allele **C** appears on the island and $m_I = 0, m_C = m$ otherwise.

$$
\begin{cases}
\dot{p_A} &= -p_A \big(m + (p_A - 1) \big(\epsilon_{AC} p_{bC}^2 + \alpha + 2p_{Bc}(\eta_{AB} + p_{BC}(\epsilon_{AB} - 2\eta_{AB})) + p_{Bc}^2(\epsilon_{AB} - 2\eta_{AB}) + p_{BC}(2(p_{bC}\epsilon_{AC} \\
&+ \eta_{AB}) + p_{BC}(\epsilon_{AB} + \epsilon_{AC} - 2\eta_{AB})) - 2(p_{bC} + p_{BC} - 1)(p_{bC} + p_{BC})\eta_{AC} + p_A \big((-4\epsilon_{AB} + 4\eta_{AB} + \theta_{AB})p_{Bc}^2 \\
&+ 2(-4p_{BC}\epsilon_{AB} + \epsilon_{AB} + 4p_{BC}\eta_{AB} - 2\eta_{AB} + p_{BC}\theta_{AB})p_{Bc} + p_{BC}^2(-4\epsilon_{AB} - 4\epsilon_{AC} + 4\eta_{AB} + 4\eta_{AC} + \theta_{AB} + \theta_{AC}) \\
&+ p_{bC}((2 - 4p_{bC})\epsilon_{AC} + 4(p_{bC} - 1)\eta_{AC} + p_{bC}\theta_{AC}) + 2p_{BC}(\epsilon_{AB} - 4p_{bC}\epsilon_{AC} + \epsilon_{AC} + 4p_{bC}\eta_{AC} \\
&- 2(\eta_{AB} + \eta_{AC}) + p_{bC}\theta_{AC}))))\\
\dot{p_{Bc}} &= m_I + p_{Bc} \big(\epsilon_{AB}p_A^2 + 4p_{bC}^2\epsilon_{AC}p_A^2 - p_{bC}\epsilon_{AC}p_A^2 - 4p_{Bc}^2\eta_{AB}p_A^2 + 6p_{Bc}\eta_{AB}p_A^2 - 2\eta_{AB}p_A^2 - 4p_{bC}^2\eta_{AC}p_A^2 \\
&+ 2p_{bC}\eta_{AC}p_A^2 - p_{Bc}^2\theta_{AB}p_A^2 + p_{Bc}\theta_{AB}p_A^2 - p_{bC}^2\theta_{AC}p_A^2 + p_{Bc}(-2p_{Bc} + p_A(4p_{Bc} - 5) + 2)\epsilon_{AB}p_A \\
&- 2p_{bC}^2\epsilon_{AC}p_A + 4p_{Bc}^2\eta_{AB}p_A - 6p_{Bc}\eta_{AB}p_A + 2\eta_{AB}p_A + 4p_{bC}^2\eta_{AC}p_A - 2p_{bC}\eta_{AC}p_A - m - (p_{Bc} + p_{BC} - 1)\beta \\
&- p_{bC}\gamma + p_{bC}\eta_{BC} - 2p_{bC}p_{Bc}\eta_{BC} - p_{BC}\big(\,((5 - 8p_{Bc})\epsilon_{AB} - 8p_{bC}\epsilon_{AC} + \epsilon_{AC} + 8p_{Bc}\eta_{AB} - 6\eta_{AB} + 8p_{bC}\eta_{AC} \\
&- 2\eta_{AC} + 2p_{Bc}\theta_{AB} - \theta_{AB} + 2p_{bC}\theta_{AC})p_A^2 + 2((2p_{Bc} - 1)\epsilon_{AB} + 2p_{bC}\epsilon_{AC} - 4p_{Bc}\eta_{AB} + 3\eta_{AB} - 4p_{bC}\eta_{AC} + \eta_{AC})p_A \\
&+ \gamma - \epsilon_{BC} + 2(p_{bC} + p_{Bc})(\epsilon_{BC} - \eta_{BC}) + 2\eta_{BC}\,\big) + p_{BC}^2(2\eta_{BC} + p_A((4p_A - 2)\epsilon_{AB} + (4p_A - 2)\epsilon_{AC} \\
&+ 4(\eta_{AB} + \eta_{AC}) - p_A(4\eta_{AB} + 4\eta_{AC} + \theta_{AB} + \theta_{AC})) - \theta_{BC})\,\big)\\
\dot{p_{bC}} &= p_{bC}\big(p_A((4p_A - 2)\epsilon_{AB} + 4\eta_{AB} - p_A(4\eta_{AB} + \theta_{AB}))p_{Bc}^2 - ((-8p_{BC}\epsilon_{AB} + \epsilon_{AB} + 8p_{BC}\eta_{AB} - 2\eta_{AB} \\
&+ 2p_{BC}\theta_{AB})p_A^2 + 2(2p_{BC}\epsilon_{AB} - 4p_{BC}\eta_{AB} + \eta_{AB})p_A + \beta + 2p_{BC}\epsilon_{BC} + 2p_{bC}\eta_{BC} - 2p_{BC}\eta_{BC} - \eta_{BC}\big)\,p_{Bc} - m \\
&- p_{BC}\big((\epsilon_{AB} - 8p_{bC}\epsilon_{AC} + 5\epsilon_{AC} - 2\eta_{AB} + 8p_{bC}\eta_{AC} - 6\eta_{AC} + 2p_{bC}\theta_{AC} - \theta_{AC})p_A^2 + 2((2p_{bC} - 1)\epsilon_{AC} + \eta_{AB} \\
&- 4p_{bC}\eta_{AC} + 3\eta_{AC})p_A + \beta + \gamma + 2p_{bC}\epsilon_{BC} - \epsilon_{BC} - 2p_{bC}\eta_{BC} + 2\eta_{BC}\,\big) - (p_{bC} - 1)(\gamma + p_A(2(p_{bC}\epsilon_{AC} \\
&- 2p_{bC}\eta_{AC} + \eta_{AC}) + p_A(-4p_{bC}\epsilon_{AC} + \epsilon_{AC} + 4p_{bC}\eta_{AC} - 2\eta_{AC} + p_{bC}\theta_{AC}))) + p_{BC}^2(2\eta_{BC} + p_A((4p_A - 2)\epsilon_{AB} \\
&+ (4p_A - 2)\epsilon_{AC} + 4(\eta_{AB} + \eta_{AC}) - p_A(4\eta_{AB} + 4\eta_{AC} + \theta_{AB} + \theta_{AC})) - \theta_{BC})\,\big)\\
\dot{p_{BC}} &= m_C - p_{BC}\big(-\epsilon_{AB}p_A^2 - 4p_{bC}^2\epsilon_{AC}p_A^2 + 5p_{bC}\epsilon_{AC}p_A^2 - \epsilon_{AC}p_A^2 + 4p_{Bc}^2\eta_{AB}p_A^2 - 6p_{Bc}\eta_{AB}p_A^2 + 2\eta_{AB}p_A^2 \\
&+ 4p_{bC}^2\eta_{AC}p_A^2 - 6p_{bC}\eta_{AC}p_A^2 + 2\eta_{AC}p_A^2 + p_{Bc}^2\theta_{AB}p_A^2 - p_{Bc}\theta_{AB}p_A^2 + (p_{bC} - 1)p_{bC}\theta_{AC}p_A^2 + p_{Bc}(-4p_{Bc}p_A \\
&+ 5p_A + 2p_{Bc} - 2)\epsilon_{AB}p_A + 2p_{bC}^2\epsilon_{AC}p_A - 2p_{bC}\epsilon_{AC}p_A - 4p_{Bc}^2\eta_{AB}p_A + 6p_{Bc}\eta_{AB}p_A - 2\eta_{AB}p_A - 4p_{bC}^2\eta_{AC}p_A \\
&+ 6p_{bC}\eta_{AC}p_A - 2\eta_{AC}p_A + m + (p_{Bc} + p_{BC} - 1)\beta + (p_{bC} - 1)\gamma - p_{bC}\epsilon_{BC} - p_{Bc}\epsilon_{BC} + p_{bC}\eta_{BC} + 2p_{bC}p_{Bc}\eta_{BC} \\
&+ p_{Bc}\eta_{BC} - \eta_{BC} + p_{BC}\big(\,-((8p_{Bc} - 5)\epsilon_{AB} + (8p_{bC} - 5)\epsilon_{AC} + 6\eta_{AB} + 6\eta_{AC} + \theta_{AB} - 2p_{Bc}(4\eta_{AB} + \theta_{AB}) + \theta_{AC} \\
&- 2p_{bC}(4\eta_{AC} + \theta_{AC}))p_A^2 + (4p_{Bc}\epsilon_{AB} - 2\epsilon_{AB} + 4p_{bC}\epsilon_{AC} - 2\epsilon_{AC} - 8p_{Bc}\eta_{AB} - 8p_{bC}\eta_{AC} + 6(\eta_{AB} + \eta_{AC}))p_A + \gamma \\
&+ 2(p_{bC} + p_{Bc})(\epsilon_{BC} - \eta_{BC}) + 3\eta_{BC} - \theta_{BC}\,\big) + p_{BC}^2\big(\,(-4\epsilon_{AB} - 4\epsilon_{AC} + 4\eta_{AB} + 4\eta_{AC} + \theta_{AB} + \theta_{AC})p_A^2 \\
&+ 2(\epsilon_{AB} + \epsilon_{AC} - 2(\eta_{AB} + \eta_{AC}))p_A - 2\eta_{BC} + \theta_{BC}\,\big)\,\big)
\end{cases}
\tag{A12}
$$

- **AB-C**: If the **A** and **B** loci are in tight linkage and the **C** locus in loose linkage, then the system of equations is equivalent to the **AC-B** architecture if allele **C** appears on the continent. One simply needs to exchange the role of the **B** and **C** loci. In that case, equation (A11) can be used to describe the dynamics of this system, with the following transformations:

$$
\beta \leftrightarrow \gamma, \eta_{AB} \leftrightarrow \eta_{AC}, \epsilon_{AB} \leftrightarrow \epsilon_{AC}, \theta_{AB} \leftrightarrow \theta_{AC}, p_{Ac} \rightarrow p_{Ab}, p_{AC} \rightarrow p_{AB}, p_{aC} \rightarrow p_{aB}, p_B \rightarrow p_C \tag{A13}
$$

If allele **C** appears on the island, then this system is equivalent to the **A-BC** architecture; with the **A** and **C** loci switching roles. In that case, equation (A12) can be used with the

following transformations:

$$\alpha \leftrightarrow \gamma, \eta_{AB} \leftrightarrow \eta_{BC}, \epsilon_{AB} \leftrightarrow \epsilon_{BC}, \theta_{AB} \leftrightarrow \theta_{BC}, p_{Bc} \rightarrow p_{aB}, p_{BC} \rightarrow p_{AB}, p_{bC} \rightarrow p_{Ab}, p_A \rightarrow p_C \tag{A14}$$

- **A-B-C**: All loci are in loose linkage. The dynamics of such a system are described in equations (A15) with $m_C = 0$ if **C** appears on the island and $m_C = m$ otherwise.

$$\begin{cases} \dot{p_A} &= p_A(-m - (-1+p_A)(\alpha + p_B(p_B(\epsilon_{AB} - 2\eta_{AB}) + 2\eta_{AB}) + p_C(p_C(\epsilon_{AC} - 2\eta_{AC}) + 2\eta_{AC}) \\ &- p_A(p_B((-2+4p_B)\epsilon_{AB} + 4\eta_{AB} - p_B(4\eta_{AB} + \theta_{AB})) \\ &+ p_C((-2+4p_C)\epsilon_{AC} + 4\eta_{AC} - p_C(4\eta_{AC} + \theta_{AC}))))) \\ \dot{p_B} &= -(-1+p_B)(m + p_B(\beta + 2p_A(p_B(\epsilon_{AB} - 2\eta_{AB}) + \eta_{AB}) - p_A^2((-1+4p_B)\epsilon_{AB} + 2\eta_{AB} \\ &- p_B(4\eta_{AB} + \theta_{AB})) - p_C(-p_C\epsilon_{BC} + 2(-1+p_C)\eta_{BC} \\ &+ p_B((-2+4p_C)\epsilon_{BC} + 4\eta_{BC} - p_C(4\eta_{BC} + \theta_{BC}))))) \\ \dot{p_C} &= m_C + p_C(-m - (-1+p_C)(\gamma + 2p_A(p_C(\epsilon_{AC} - 2\eta_{AC}) + \eta_{AC}) - p_A^2((-1+4p_C)\epsilon_{AC} + 2\eta_{AC} \\ &- p_C(4\eta_{AC} + \theta_{AC})) - p_B(-2(p_C(\epsilon_{BC} - 2\eta_{BC}) + \eta_{BC}) \\ &+ p_B((-1+4p_C)\epsilon_{BC} + 2\eta_{BC} - p_C(4\eta_{BC} + \theta_{BC}))))) \end{cases} \tag{A15}$$

A 4 Role of the different loci

Table A3 displays all possible evolutionary histories. Each history can be described by an ancestral haplotype, the order in which new mutations appear and where those mutations happen. We show here that we can assume that **abc** is always the ancestral haplotype. This is achieved either through simple relabeling (if **C** is not ancestral) or through more complex re-parametrization (if **C** is ancestral). The results for the three-locus model differs from the two-locus model, in the sense that relabeling is no longer enough to describe all potential histories. This result is obtained by fully parametrizing the system and therefore adding a seventh parameter, describing three-locus interaction. We choose to define the fitness of the haplotype **abC** by $\gamma + \epsilon_{abC}$.

If allele **c** is the ancestral allele at locus **C**, we recover the results from Bank et al. [2012]: using **abc** as the ancestral haplotype captures all the different scenarios described in table A3 upon relabeling. For example, if we consider **Abc** as the ancestral haplotype, with mutation appearing in the following order: first **a** on the continent, then **B** on the continent and finally **C**,

then this scenario is absolutely equivalent to having **abc** as the ancestral haplotype and the "Isl-Cont-Mod" scenario. When allele **C** is the ancestral allele, this is no longer true: relabeling the different alleles is not sufficient to recover the case "**abc** is the ancestral haplotype". However, when using seven parameters, the system is fully parametrized. Therefore, by transforming the different parameters, we can draw an equivalence between the current scenario and the scenario with haplotype **abc**. For example, if **aBC** is the ancestral haplotype, and new mutations appears in the following order: **b** then **A** and lastly **c**, then this is equivalent to the "Cont-Isl-Mod" scenario with **abc** as the ancestral haplotype if one transform the different parameters, using the correspondence given in the table A3. Therefore using only **abc** as the ancestral haplotype would be sufficient to cover all cases as long as the system is fully parametrized. Opposite to the two-locus case, for some scenarios the transformation is no longer trivial.

References

C. Bank, R. Bürger, and J. Hermisson. The Limits to Parapatric Speciation: Dobzhansky–Muller Incompatibilities in a Continent–Island Model. *Genetics*, 191(3):845–863, 2012.

ancestral haplotype	1st mut.	2nd mut.	3rd mut.	Definition	
**abc**	**A**	**B**	**C**	Isl-Cont-Mod	
**abc**	**A**	**C**	**B**	Isl-Mod-Cont	
**abc**	**B**	**A**	**C**	Cont-Isl-Mod	
**abc**	**B**	**C**	**A**	Cont-Mod-Isl	
**abc**	**C**	**A**	**B**	Mod-Isl-Cont	
**abc**	**C**	**B**	**A**	Mod-Cont-Isl	
anc. hap.				equivalent. to	Transformation
**Abc**	**a**	**B**	**C**	Isl-Cont-Mod	
**Abc**	**a**	**C**	**B**	Isl-Mod-Cont	**a** $\leftrightarrow$ **A**
**Abc**	**C**	**a**	**B**	Mod-Isl-Cont	
**abC**	**A**	**B**	**c**	Isl-Cont-Mod	
**abC**	**A**	**c**	**B**	Isl-Mod-Cont	
**abC**	**B**	**A**	**c**	Cont-Isl-Mod	$\alpha^* = \alpha + \epsilon_{AC} - \epsilon_{abC}, \beta^* = \beta + \epsilon_{BC} - \epsilon_{abC},$
**abC**	**B**	**c**	**A**	Cont-Mod-Isl	$\gamma^* = -\gamma, \epsilon^*_{AB} = \epsilon_{AB} + \epsilon_{abC},$
**abC**	**c**	**A**	**B**	Mod-Isl-Cont	$\epsilon^*_{BC} = -\epsilon_{BC}, \epsilon^*_{AC} = -\epsilon_{AC}, \epsilon^*_{abC} = -\epsilon_{abC}$
**abC**	**c**	**B**	**A**	Mod-Cont-Isl	
**AbC**	**a**	**B**	**c**	Isl-Cont-Mod	$\alpha^* = \epsilon_{abC} - \alpha - \epsilon_{AC}, \beta^* = \beta + \epsilon_{AB} + \epsilon_{BC},$
**AbC**	**a**	**c**	**B**	Isl-Mod-Cont	$\gamma^* = -(\gamma + \epsilon_{abC} + \epsilon_{AC}), \epsilon^*_{AB} = -(\epsilon_{AB} + \epsilon_{abC}),$
**AbC**	**c**	**a**	**B**	Mod-Isl-Cont	$\epsilon^*_{BC} = \epsilon_{abC} - \epsilon_{BC}, \epsilon^*_{AC} = \epsilon_{AC}, \epsilon^*_{abC} = \epsilon_{abC}$
**aBc**	**b**	**A**	**C**	Cont-Isl-Mod	
**aBc**	**b**	**C**	**A**	Cont-Mod-Isl	**b** $\leftrightarrow$ **B**
**aBc**	**C**	**b**	**A**	Mod-Cont-Isl	
**aBC**	**b**	**A**	**c**	Cont-Isl-Mod	$\alpha^* = \alpha + \epsilon_{AB} + \epsilon_{AC}, \beta^* = \epsilon_{abC} - \beta - \epsilon_{BC},$
**aBC**	**b**	**c**	**A**	Cont-Mod-Isl	$\gamma^* = -(\gamma + \epsilon_{BC} + \epsilon_{abC}), \epsilon^*_{AB} = -(\epsilon_{AB} + \epsilon_{abC}),$
**aBC**	**c**	**b**	**A**	Mod-Cont-Isl	$\epsilon^*_{BC} = \epsilon_{BC}, \epsilon^*_{AC} = \epsilon_{abC} - \epsilon_{AC}, \epsilon^*_{abC} = \epsilon_{abC}$

Table A3: **All possible evolutionary histories and how they can always be reduced to abc being the ancestral type**
For each ancestral haplotype, we give all possible evolutionary histories. We precise the new allele at each loci as well as their order of mutation. The first part of the table defines the different evolutionary scenarios with **abc** as the ancestral haplotype. Mod. stands for modifier and simply indicates where the mutation at locus **C** happens. This allows us to give a condense expression without differentiating between **C** appearing on the island or on the continent. The second part of the table consider all the other possible ancestral haplotypes. We then indicate to which of the 6 scenarios defined above they correspond to and which transformation are necessary to be equivalent to this scenario but with **abc** as the ancestral haplotype. It can either be achieved through relabeling ($x \leftrightarrow X$) or re-parametrization ($x* = f(x)$). Note that the transformation only depends on the ancestral haplotype and not on the order of mutations.
