# Supplementary material for: The Limits to Parapatric Speciation II: Strengthening a Preexisting Genetic Barrier to Gene Flow in Parapatry
Source: Genetics. 2018 Feb 28;209(1):241–54. doi: 10.1534/genetics.117.300652 (PMC5937195; doi:10.1534/genetics.117.300652)
Supplement: Supplementary file 2 [file 241FileS2.pdf]

Supplementary File B: Adaptation at existing barrier loci

Alexandre Blanckaert *[1,2] and Joachim Hermisson[1,3]

[1]Department of Mathematics, University of Vienna, 1090 Vienna, Austria

[2]Instituto Gulbenkian de Ciência, 2780-156 Oeiras, Portugal

[3]Mathematics and Biosciences Group, Max F. Perutz Laboratories, 1030 Vienna, Austria

February 28, 2018

In this Supplementary file, we focus on the impact of further mutations at one of barrier loci. We consider that A has 3 alleles **a**, **A** and **A'**.

B 1 Expression for the strength of genetic barrier

The system of equations describing the dynamics of such system can be obtained through equation (A7) with the following substitutions:

$$\dot{p_{AC}} = p_{AC} = 0, p_{Ac} \to p_A, p_{aC} \to p_{A'}, \gamma \to \alpha', \epsilon_{BC} \to \epsilon_{A'B} \tag{B1}$$

This leads to equation 2. Such system has 9 equilibriums, some are shared with the two diallelic model. They correspond to the boundary solutions when **A** or **A'** are absent: fixation of the continental haplotype, fixation of allele **B** (S_A or S_A'), loss of allele **A** (resp. **A'**) S_B, stable and unstable two-locus polymorphism equilibriums S_{AB}^{stable}, $S_{AB}^{unstable}$, $S_{A'B}^{stable}$ and $S_{A'B}^{unstable}$. In addition, there is one equilibrium with both **A** and **A'** polymorphic at the same time, $S_{AA'B}$, given by:

$$\begin{cases} p_A = & \frac{\beta+\epsilon_{AB}}{-\epsilon_{AB}+\epsilon_{A'B}} + \frac{\alpha' m \epsilon_{AB}}{\alpha\alpha'\epsilon_{AB}-\alpha^2\epsilon_{A'B}} + \frac{\alpha^2-\alpha\alpha'+\alpha' m}{\alpha^2-\alpha\alpha'} \\ p_{A'} = & \frac{\epsilon_{AB}+\beta}{\epsilon_{AB}-\epsilon_{A'B}} + m\left(\frac{\epsilon_{AB}}{\alpha\epsilon_{A'B}-\alpha'\epsilon_{AB}} + \frac{1}{\alpha'-\alpha}\right) \\ p_B = & \frac{\alpha'-\alpha}{\epsilon_{AB}-\epsilon_{A'B}} \end{cases} \tag{B2}$$

*ablanckaert@igc.gulbenkian.pt

The internal equilibrium exists and is strictly internal only if one of the two following conditions holds (from the expression of p_B):

$$\begin{cases} cond_1 = \epsilon_{AB} > \epsilon_{A'B} \text{ and } \alpha \le \alpha' \le \alpha + \epsilon_{AB} - \epsilon_{A'B} \\ cond_2 = \epsilon_{AB} < \epsilon_{A'B} \text{ and } \alpha + \epsilon_{AB} - \epsilon_{A'B} \le \alpha' \le \alpha \end{cases} \tag{B3}$$

In addition, equation (B4) gives for which migration rates the $S_{A'B}^{stable}$ equilibrium can be stable. The expression of this equilibrium can be deduced from Bank et al. [2012] (equations (S.91) and (S.92)). Note that equation (B4) also holds for the $S_{A'B}^{stable}$ equilibrium if the proper substitutions are applied ($\alpha \leftrightarrow \alpha'$ and $\epsilon_{AB} \leftrightarrow \epsilon_{A'B}$).

$$\left.\begin{array}{ll} \text{if } cond_5 & \frac{(\alpha-\alpha')(\beta+\epsilon_{A'B})(\alpha'\epsilon_{AB}-\alpha\epsilon_{A'B})}{\alpha'(\epsilon_{AB}-\epsilon_{A'B})^2} \\ \text{else} & 0 \end{array}\right\} = m_{min}^{A'b} \le m \le m_{max}^{A'b} = \begin{cases} -\frac{(\alpha'+\epsilon_{A'B})(\beta+\epsilon_{A'B})}{\alpha'} & \text{if } cond_1 \\ \frac{\alpha'\beta}{\beta+\epsilon_{A'B}} & \text{if } cond_2 \\ \frac{\alpha'(\beta+\epsilon_{A'B})}{4\epsilon_{A'B}} & \text{if } cond_3 \\ \frac{(\alpha-\alpha')(\beta+\epsilon_{A'B})(\alpha'\epsilon_{AB}-\alpha\epsilon_{A'B})}{\alpha'(\epsilon_{AB}-\epsilon_{A'B})^2} & \text{if } cond_4 \end{cases}$$

$$\begin{aligned}
cond_1 = \;& \text{Max}[0,\beta] \le -\epsilon_{A'B} \le \text{Min}[\tfrac{\alpha'}{2}, \alpha'+\beta] \text{ and } ((\epsilon_{A'B} > \epsilon_{AB} \text{ and } \alpha \le \alpha') \text{ or } (\alpha + \epsilon_{AB} < \alpha' + \epsilon_{A'B} \text{ and} \\
& \epsilon_{AB} > \epsilon_{A'B} \text{ and } 2\alpha > \tfrac{\alpha'\epsilon_{AB}}{\epsilon_{A'B}} + \alpha') \text{ or } (((\alpha' = -2\epsilon_{A'B} \text{ and } \alpha'\epsilon_{AB} < \epsilon_{A'B}(\alpha - \epsilon_{AB} + \epsilon_{A'B})) \text{ or} \\
& (\alpha' > -2\epsilon_{A'B} \text{ and } \alpha + \epsilon_{AB} < \alpha' + \epsilon_{A'B})) \text{ and } \tfrac{\alpha'(\epsilon_{AB}+\epsilon_{A'B})}{2\epsilon_{A'B}} > \alpha > \alpha') \text{ or} \\
& (\alpha'(\epsilon_{AB} + \epsilon_{A'B}) < 2\alpha\epsilon_{A'B} \text{ and } \epsilon_{A'B} \le \epsilon_{AB})) \\
cond_2 = \;& \alpha' \ge 0 \text{ and Max}[0, \alpha'+\beta] + \epsilon_{A'B} \le 0 \text{ and } \beta \le \epsilon_{A'B} \text{ and } ((\epsilon_{A'B} > \epsilon_{AB} \text{ and } \alpha \le \alpha') \text{ or } ((\alpha'+\beta \ge 0 \text{ or} \\
& \alpha'(\beta + \epsilon_{AB}) \le \alpha(\beta + \epsilon_{A'B})) \text{ and } (\alpha' + \beta < 0 \text{ or } \alpha'(\beta + \epsilon_{AB}) < \alpha(\beta + \epsilon_{A'B})) \text{ and } \beta < \epsilon_{A'B} \text{ and} \\
& \epsilon_{A'B} < \epsilon_{AB} \text{ and } \tfrac{\alpha'(\epsilon_{AB}+\epsilon_{A'B})}{2\epsilon_{A'B}} < \alpha < \alpha') \text{ or } (\alpha'(\beta + \epsilon_{AB}) < \alpha(\beta + \epsilon_{A'B}) \text{ and } \tfrac{\alpha'(\epsilon_{AB}+\epsilon_{A'B})}{2\epsilon_{A'B}} > \alpha > \alpha') \text{ or} \\
& (\alpha'(\epsilon_{AB} + \epsilon_{A'B}) < 2\alpha\epsilon_{A'B} \text{ and } \epsilon_{A'B} \le \epsilon_{AB})) \\
cond_3 = \;& \alpha' \ge 0 \text{ and Max}[\tfrac{\alpha'}{2}, |\beta|] + \epsilon_{A'B} \le 0 \text{ and } ((\epsilon_{A'B} > \epsilon_{AB} \text{ and } \alpha \le \alpha') \text{ or } (\beta \ge \epsilon_{A'B} \text{ and} \\
& \tfrac{\alpha'(\epsilon_{AB}+\epsilon_{A'B})}{2\epsilon_{A'B}} > \alpha > \alpha') \text{ or } (\alpha'(\epsilon_{AB} + \epsilon_{A'B}) < 2\alpha\epsilon_{A'B} \text{ and } \epsilon_{A'B} \le \epsilon_{AB})) \\
cond_4 = \;& \alpha' \ge 0 \text{ and } \epsilon_{A'B} < \epsilon_{AB} \text{ and } \tfrac{\alpha'(\epsilon_{AB}+\epsilon_{A'B})}{2\epsilon_{A'B}} < \alpha < \alpha' \text{ and } (((\alpha' = -2\epsilon_{A'B} \text{ or } (\alpha' > -2\epsilon_{A'B} \text{ and} \\
& \alpha + \epsilon_{AB} \ge \alpha' + \epsilon_{A'B})) \text{ and Max}[0,\beta] \le -\epsilon_{A'B} \le \text{Min}[\tfrac{\alpha'}{2}, \alpha'+\beta]) \text{ or } ((\beta \ge \epsilon_{A'B} \text{ or } (\alpha' + \beta < 0 \text{ and} \\
& \alpha(\beta + \epsilon_{A'B}) < \alpha'(\beta + \epsilon_{AB})) \text{ or } (\alpha' + \beta \ge 0 \text{ and } \alpha(\beta + \epsilon_{A'B}) \le \alpha'(\beta + \epsilon_{AB}))) \text{ and} \\
& \text{Max}[0, \alpha'+\beta] + \epsilon_{A'B} \le 0 \text{ and } \beta \le \epsilon_{A'B}) \text{ or } (\beta \ge \epsilon_{A'B} \text{ and Max}[\tfrac{\alpha'}{2}, |\beta|] + \epsilon_{A'B} \le 0)) \\
cond_5 = \;& \tfrac{\alpha'(\epsilon_{AB}+\epsilon_{A'B})}{2\epsilon_{A'B}} > \alpha > \alpha' \text{ and } ((((\alpha' = -2\epsilon_{A'B} \text{ and } \alpha'\epsilon_{AB} < \epsilon_{A'B}(\alpha - \epsilon_{AB} + \epsilon_{A'B})) \text{ or } (\alpha' > -2\epsilon_{A'B} \text{ and} \\
& \alpha + \epsilon_{AB} < \alpha' + \epsilon_{A'B})) \text{ and Max}[0,\beta] \le -\epsilon_{A'B} \le \text{Min}[\tfrac{\alpha'}{2}, \alpha'+\beta]) \text{ or } (\text{Max}[0, \alpha'+\beta] + \epsilon_{A'B} \le 0 \text{ and} \\
& \beta \le \epsilon_{A'B} \text{ and } \alpha'(\beta + \epsilon_{AB}) < \alpha(\beta + \epsilon_{A'B})) \text{ or } (\beta \ge \epsilon_{A'B} \text{ and Max}[\tfrac{\alpha'}{2}, |\beta|] + \epsilon_{A'B} \le 0))
\end{aligned} \tag{B4}$$

B 2 Proof that the A and A' alleles can not coexist in a stable way

One of the main result from Nagylaki and Lou [2001] is that two alleles at most can coexist in a stable way. Here, we prove that this result still holds when the locus interacts epistatically with another one.

Consider the matrix J (equation (B5)), the Jacobian of the system, given in equation (2), at the internal fixed point, $S_{AA'B}$, given in equation (B2).

$$J = \begin{pmatrix} j_1 & j_1 & j_2 \\ j_3 & j_3 & j_4 \\ \epsilon_{AB} j_5 & \epsilon_{A'B} j_5 & j_6 \end{pmatrix}$$

$$\begin{aligned}
j_1 &= \frac{\epsilon_{AB}^2 m - 2\epsilon_{AB}\epsilon_{A'B} m + \epsilon_{A'B}(-\alpha\beta - \alpha\epsilon_{A'B} + \epsilon_{A'B} m)}{(\epsilon_{AB} - \epsilon_{A'B})^2} + \frac{\epsilon_{AB}\alpha'(\beta + \epsilon_{A'B})}{(\epsilon_{AB} - \epsilon_{A'B})^2} + \frac{\alpha m}{\alpha' - \alpha} \\
j_2 &= \left(\alpha' \left(\epsilon_{AB}^2 m + \epsilon_{AB}\alpha'(\beta + \epsilon_{A'B}) - \epsilon_{AB}(\alpha\beta + \alpha\epsilon_{A'B} + 2\epsilon_{A'B} m) + \epsilon_{A'B}(-\alpha\beta - \alpha\epsilon_{A'B} + \epsilon_{A'B} m)\right) + \alpha^2\epsilon_{A'B}(\beta + \epsilon_{A'B})\right) \\
&\quad * \frac{\left(\alpha\beta - \alpha'(\beta + \epsilon_{AB}) + \epsilon_{AB}(\alpha - m) + \epsilon_{A'B} m\right)}{(\alpha - \alpha')^2(\epsilon_{AB} - \epsilon_{A'B})(\epsilon_{AB}\alpha' - \alpha\epsilon_{A'B})} \\
j_3 &= \frac{(\beta + \epsilon_{AB})\left(\alpha\epsilon_{A'B} - \epsilon_{AB}\alpha'\right)}{(\epsilon_{AB} - \epsilon_{A'B})^2} + \frac{\alpha m}{\alpha - \alpha'} \\
j_4 &= \frac{\left(-\alpha\beta + \epsilon_{AB} m + \alpha'(\beta + \epsilon_{A'B}) - \epsilon_{A'B}(\alpha + m)\right)\left(\alpha\left(\epsilon_{AB}^2 m + \epsilon_{AB}\epsilon_{A'B}(\alpha - 2m) + \epsilon_{A'B}(\alpha\beta + \epsilon_{A'B} m)\right) + \alpha'(\beta + \epsilon_{AB})\left(\epsilon_{AB}\alpha' - \alpha(\epsilon_{AB} + \epsilon_{A'B})\right)\right)}{(\alpha - \alpha')^2(\epsilon_{AB} - \epsilon_{A'B})(\epsilon_{AB}\alpha' - \alpha\epsilon_{A'B})} \\
j_5 &= -\frac{\left(\alpha - \alpha'\right)\left(-\alpha' + \alpha + \epsilon_{AB} - \epsilon_{A'B}\right)}{(\epsilon_{AB} - \epsilon_{A'B})^2} \\
j_6 &= \frac{m(\epsilon_{AB} - \epsilon_{A'B})}{\alpha - \alpha'} + m
\end{aligned} \tag{B5}$$

Then the maximal eigenvalue can be written as:

$$\Lambda(J) = Max_v(< v, Jv > / < v, v >) \tag{B6}$$

where $< .,. >$ is the scalar product and v some vector. Take, in particular, $v = (1, -1, 0)$. Then we find $< v, Jv >= 0$ and therefore $\Lambda(J) \geq 0$.

If $\Lambda(J) = 0$, we need an eigenvalue 0 of J, thus the row and column vectors are linearly dependent. The first and second column vector are linearly dependent if and only if $\epsilon_{AB} = \epsilon_{A'B}$. If not, linear combinations of these two vectors will give us the column vectors $(1, 1, 0)$ and $(0, 0, 1)$. The third column vector (j_2, j_4, j_6) is linear dependent of these if and only if its first two entries are equal: $j_2 = j_4$. If not, 0 is not an eigenvalue and we have $\Lambda(J) > 0$.

Otherwise, if $\epsilon_{AB} = \epsilon_{A'B}$, but $j_2 \neq j_4$. Consider $v = (1, -1, \pm\delta)$. For small enough δ, we get $< v, Jv >$ with opposite sign for + and - δ and thus $\Lambda(J) > 0$. The same holds if $j_2 = j_4$, but $\epsilon_{AB} \neq \epsilon_{A'B}$. Finally, if both are equal, we need to have $\epsilon_{AB} = \epsilon_{A'B}$ and $\alpha = \gamma$.

B 3 Conditions under which invasion of S_{AB}^{stable} by allele A' leads to the loss of A

We now investigate the consequence of the apparition of allele **A'**. One important outcome is the destruction of the genetic barrier. While this is impossible for a single-locus barrier, this no longer holds true when extended to multiple interacting loci.

Conditions for invasibility of the S_{AB}^{stable} equilibrium by allele **A'** are given in equation (B7).

$$
\left.\begin{array}{ll}
\text{if } cond_5 & \frac{(\alpha-\alpha')(\beta+\epsilon_{AB})(\alpha'\epsilon_{AB}-\alpha\epsilon_{A'B})}{\alpha(\epsilon_{AB}-\epsilon_{A'B})^2} \\
\text{if } cond_6 & 0
\end{array}\right\} = m_{min}^{inv} \leq m \leq m_{max}^{inv} = \begin{cases}
-\frac{(\alpha+\epsilon_{AB})(\beta+\epsilon_{AB})}{\alpha} & \text{if } cond_1 \\
\frac{\alpha\beta}{\beta+\epsilon_{AB}} & \text{if } cond_2 \\
\frac{\alpha(\beta+\epsilon_{AB})}{4\epsilon_{AB}} & \text{if } cond_3 \\
\frac{(\alpha-\alpha')(\beta+\epsilon_{AB})(\alpha'\epsilon_{AB}-\alpha\epsilon_{A'B})}{\alpha(\epsilon_{AB}-\epsilon_{A'B})^2} & \text{if } cond_4
\end{cases}
$$

$$
\begin{aligned}
cond_1 = \;& \text{Min}[\tfrac{\alpha}{2}, \alpha+\beta] + \epsilon_{AB} \geq 0 \text{ and } (((\epsilon_{AB}-\epsilon_{A'B})^2(\alpha-\alpha'+\epsilon_{AB}-\epsilon_{A'B})(\epsilon_{AB}(\alpha'+\epsilon_{AB})-\epsilon_{A'B}(\alpha+\epsilon_{AB}))<0 \\
& \text{ and } \alpha<\alpha' \text{ and } \alpha(\epsilon_{AB}+\epsilon_{A'B}) \leq 2\alpha'\epsilon_{AB}) \text{ or } (2\alpha'\epsilon_{AB} < \alpha(\epsilon_{AB}+\epsilon_{A'B}) \text{ and} \\
& (((\epsilon_{AB}-\epsilon_{A'B})^2(\alpha-\alpha'+\epsilon_{AB}-\epsilon_{A'B})(\epsilon_{AB}(\alpha'+\epsilon_{AB})-\epsilon_{A'B}(\alpha+\epsilon_{AB}))>0 \text{ and } \alpha'<\alpha) \\
& \text{ or } \epsilon_{A'B} \leq \epsilon_{AB})) \text{ or } (\alpha \leq \alpha' \text{ and } \epsilon_{A'B} > \epsilon_{AB})) \\
cond_2 = \;& \text{Max}[0, \alpha+\beta] + \epsilon_{AB} \leq 0 \text{ and } \beta \leq \epsilon_{AB} \text{ and } ((\alpha<\alpha' \text{ and } \tfrac{\alpha\beta}{\beta+\epsilon_{AB}} < \tfrac{(\alpha-\alpha')(\beta+\epsilon_{AB})(\alpha'\epsilon_{AB}-\alpha\epsilon_{A'B})}{\alpha(\epsilon_{AB}-\epsilon_{A'B})^2} \text{ and} \\
& \alpha(\epsilon_{AB}+\epsilon_{A'B}) \leq 2\alpha'\epsilon_{AB}) \text{ or } (2\alpha'\epsilon_{AB} < \alpha(\epsilon_{AB}+\epsilon_{A'B}) \text{ and } ((\tfrac{\alpha\beta}{\beta+\epsilon_{AB}} > \tfrac{(\alpha-\alpha')(\beta+\epsilon_{AB})(\alpha'\epsilon_{AB}-\alpha\epsilon_{A'B})}{\alpha(\epsilon_{AB}-\epsilon_{A'B})^2} \text{ and} \\
& \alpha'<\alpha) \text{ or } \epsilon_{A'B} \leq \epsilon_{AB})) \text{ or } (\alpha \leq \alpha' \text{ and } \epsilon_{A'B} > \epsilon_{AB})) \\
cond_3 = \;& \text{Max}[\tfrac{\alpha}{2}, |\beta|] + \epsilon_{AB} \leq 0 \text{ and } ((2\alpha'\epsilon_{AB} < \alpha(\epsilon_{AB}+\epsilon_{A'B}) \text{ and } (\alpha'<\alpha \text{ or} \\
& \epsilon_{A'B} \leq \epsilon_{AB})) \text{ or } (\epsilon_{A'B} > \epsilon_{AB} \text{ and } \alpha \leq \alpha')) \\
cond_4 = \;& \alpha<\alpha' \text{ and } \alpha(\epsilon_{AB}+\epsilon_{A'B}) \leq 2\alpha'\epsilon_{AB} \text{ and } ((\text{Min}[\tfrac{\alpha}{2}, \alpha+\beta]+\epsilon_{AB} \geq 0 \text{ and} \\
& \tfrac{(\alpha-\alpha'+\epsilon_{AB}-\epsilon_{A'B})(\epsilon_{AB}(\alpha'+\epsilon_{AB})-\epsilon_{A'B}(\alpha+\epsilon_{AB}))}{(\epsilon_{AB}-\epsilon_{A'B})^2} \geq 0) \text{ or } (\text{Max}[0,\alpha+\beta]+\epsilon_{AB} \leq 0 \text{ and } \beta \leq \epsilon_{AB} \text{ and} \\
& \tfrac{\alpha\beta}{\beta+\epsilon_{AB}} \geq \tfrac{(\alpha-\alpha')(\beta+\epsilon_{AB})(\alpha'\epsilon_{AB}-\alpha\epsilon_{A'B})}{\alpha(\epsilon_{AB}-\epsilon_{A'B})^2}) \text{ or } \text{Max}[\tfrac{\alpha}{2}, |\beta|]+\epsilon_{AB} \leq 0) \\
cond_5 = \;& \alpha'<\alpha \text{ and } 2\alpha'\epsilon_{AB} < \alpha(\epsilon_{AB}+\epsilon_{A'B}) \text{ and } ((\text{Min}[\tfrac{\alpha}{2}, \alpha+\beta]+\epsilon_{AB} \geq 0 \text{ and} \\
& \tfrac{(\alpha-\alpha'+\epsilon_{AB}-\epsilon_{A'B})(\epsilon_{AB}(\alpha'+\epsilon_{AB})-\epsilon_{A'B}(\alpha+\epsilon_{AB}))}{(\epsilon_{AB}-\epsilon_{A'B})^2} \geq 0) \text{ or } (\text{Max}[0,\alpha+\beta]+\epsilon_{AB} \leq 0 \text{ and } \beta \leq \epsilon_{AB} \text{ and} \\
& \tfrac{\alpha\beta}{\beta+\epsilon_{AB}} \geq \tfrac{(\alpha-\alpha')(\beta+\epsilon_{AB})(\alpha'\epsilon_{AB}-\alpha\epsilon_{A'B})}{\alpha(\epsilon_{AB}-\epsilon_{A'B})^2}) \text{ or } \text{Max}[\tfrac{\alpha}{2}, |\beta|]+\epsilon_{AB} \leq 0) \\
cond_6 = \;& (\epsilon_{A'B} > \epsilon_{AB} \text{ and } \alpha \leq \alpha') \text{ or } (2\alpha'\epsilon_{AB} < \alpha(\epsilon_{AB}+\epsilon_{A'B}) \text{ and } \epsilon_{A'B} \leq \epsilon_{AB}) \text{ or} \\
& (\alpha<\alpha' \text{ and } \alpha(\epsilon_{AB}+\epsilon_{A'B}) \leq 2\alpha'\epsilon_{AB})
\end{aligned}
\tag{B7}
$$

If a globally stable $S_{A'B}^{stable}$ exists, invasion of **A'** will obviously lead to the replacement of **A** by **A'**. This holds under the condition

$$
!\Big(\Big(\alpha' + \epsilon_{A'B} < \alpha + \epsilon_{AB} \text{ and } \alpha + \epsilon_{AB} > m > -\tfrac{(\alpha+\epsilon_{AB})(\beta+\epsilon_{AB})}{\alpha}\Big) \text{ or } m > \mathrm{Max}[-\beta, \alpha + \epsilon_{AB}, \alpha' + \epsilon_{A'B}]
$$
$$
\text{or } -\beta > m > \mathrm{Max}\left[\tfrac{\alpha\beta}{\beta+\epsilon_{AB}}, \tfrac{\alpha'\beta}{\beta+\epsilon_{A'B}}\right] \text{ or } \Big(\alpha + \epsilon_{AB} < \alpha' + \epsilon_{A'B} \text{ and } \alpha' + \epsilon_{A'B} > m > -\tfrac{(\alpha'+\epsilon_{A'B})(\beta+\epsilon_{A'B})}{\alpha'}\Big)\Big) \tag{B8}
$$

in addition to (B4) and (B7), see Fig. B1(a). If $S^{stable}_{A'B}$ exists, but is only locally stable, numerical observations indicate that invasion of the S^{stable}_{AB} equilibrium by **A'** still leads to replacement of the **A** allele. Here, we prove this observation for the following case: the dynamics stay in the plane defined by $\dot{p}_B = 0$ or $p_B = \frac{-m}{\beta + p_A\epsilon_{AB} + p'_A\epsilon_{A'B}}$. Because this plane is an attractor in the p_B direction, this result is also true as long as the trajectories stay close enough to this plane, which should be true if all parameters are small enough.

Secondly, we can be sure that invasion leads to replacement if there is an area between the stable and unstable equilibriums where the flow is up and to the right (Fig. B1(b), B1(d), B1(f), B1(g), B1(h)); this area does not encompass the unstable equilibrium $S^{unstable}_{AB'}$ (Fig. B1(e) and B1(i)). This means that the isocline going through S^{stable}_{AB} exits the simplex in $p_A = 0$. The conditions are described in equation (B9). If this happens, then it guarantees the existence of the area described above and therefore invasion of S^{stable}_{AB} always leads to replacement.

Lastly, when the red isocline does not cross $p_A = 0$ (Fig. B1(e) and B1(i)), we can still show that invasion leads to replacement. Indeed, let assume the opposite, i.e. the the trajectory from S^{stable}_{AB} crosses the cyan isocline and then ends up with both **A** and **A'** lost. Then, in that case, it is impossible to draw the trajectory that ends exactly in $S^{unstable}_{A'B}$ since it cannot cross the trajectory coming from S^{stable}_{AB}. Therefore, this case is impossible and the trajectory from S^{stable}_{AB} cannot cross the cyan isocline. Therefore, this trajectory always ends in $S^{stable}_{A'B}$.

$$
\begin{cases}
\frac{(\alpha-\alpha')(\beta+\epsilon_{A'B})}{\beta+\epsilon_{AB}} \leq m \leq \frac{\alpha\beta}{\beta+\epsilon_{AB}} & \text{if } \alpha'\beta < \alpha\epsilon_{A'B} \\
\frac{(\alpha-\alpha')(\beta+\epsilon_{A'B})}{\beta+\epsilon_{AB}} \leq m \leq \frac{(\alpha\epsilon_{A'B}+\alpha'\beta)^2}{4\alpha'\epsilon_{A'B}(\beta+\epsilon_{AB})} & \text{if } \frac{\alpha'\beta}{\epsilon_{A'B}} \leq \alpha < \alpha'\left(\frac{\beta}{\epsilon_{A'B}} + 2\right)
\end{cases} \tag{B9}
$$

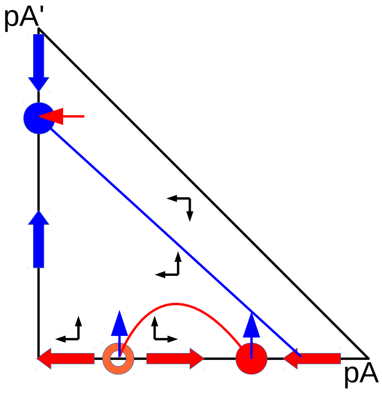

(a) $S_{A'B}^{stable}$ GS

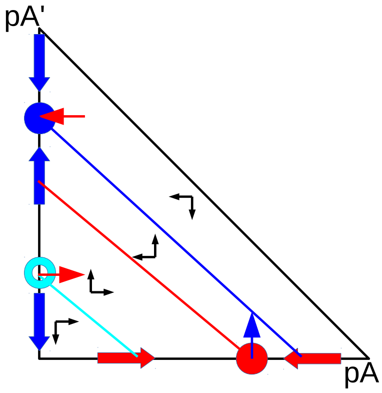

(b) S_{AB}^{stable} GS in $\{p_A, p_B\}$ and **A** invades $S_{A'B}^{unstable}$

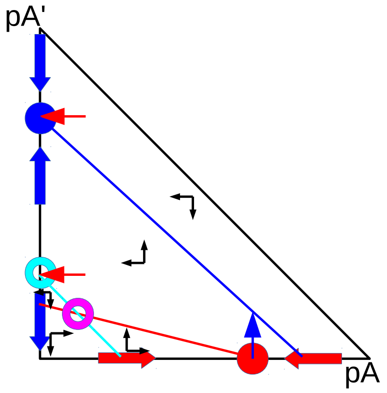

(c) S_{AB}^{stable} GS in $\{p_A, p_B\}$ and **A** does not invade $S_{A'B}^{unstable}$

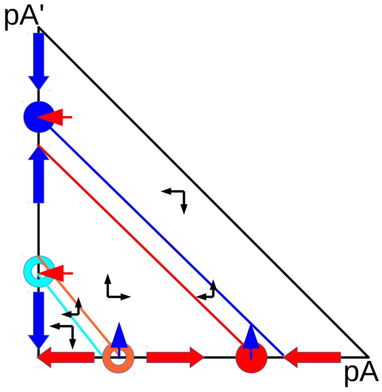

(d) **A** does not invade $S_{A'B}^{unstable}$ and **A'** invades $S_{AB}^{unstable}$

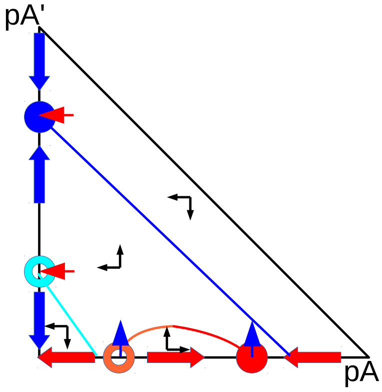

(e) **A** does not invade $S_{A'B}^{unstable}$ and **A'** invades $S_{AB}^{unstable}$

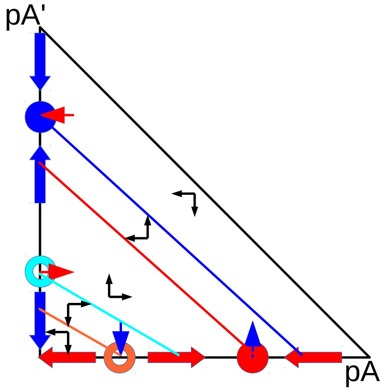

(f) **A** invades $S_{A'B}^{unstable}$ and **A** doesn't invade $S_{AB}^{unstable}$

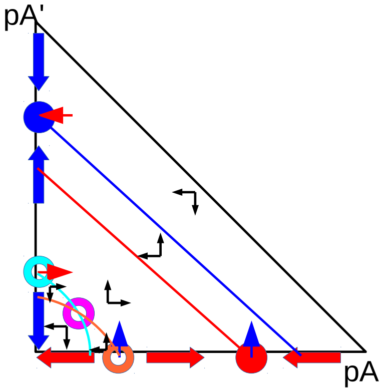

(g) **A** invades $S_{A'B}^{unstable}$ and **A'** invades $S_{AB}^{unstable}$

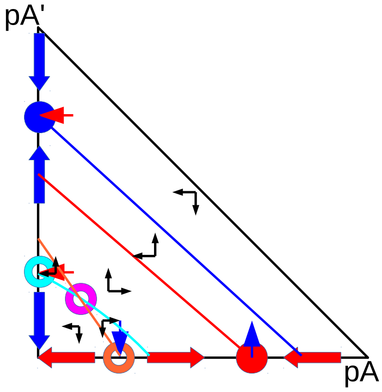

(h) **A** does not invade $S_{A'B}^{unstable}$ and **A'** does not invade $S_{AB}^{unstable}$

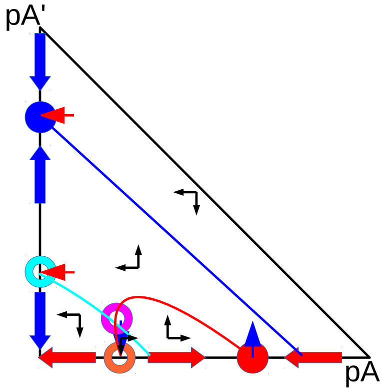

(i) **A** does not invade $S_{A'B}^{unstable}$ and **A'** does not invade $S_{AB}^{unstable}$

Figure B1: **Dynamics of the system in the plane defined by $\dot{p}_B = 0$**

The axes correspond to the frequencies of the **A** and **A'** alleles. Dots indicates equilibria, with filled dots representing stable equilibria on the border and circles unstable ones. More precisely, the red dot corresponds to the S_{AB}^{stable} equilibrium, the blue one to $S_{A'B}^{stable}$, the orange one to $S_{AB}^{unstable}$, the cyan one to $S_{A'B}^{unstable}$ and the magenta one to $S_{AA'B}$. Black arrows represent the direction of the trajectories in each part of the plane. Red, orange, blue and cyan lines correspond to the isoclines for p_A and p'_A. GS stands for globally stable.

B 4 Two-locus-three-alleles model: an alternative to secondary contact

In Fig. 2 of the main manuscript, we illustrate how a third allele in a two-locus model can bypass the local stability of an equilibrium in 2-locus-2-alleles model and can lead to the population switching between two locally stable equilibria. Fig. 2 in the main text illustrates a case where **B** started fixed on the island as it is advantageous there. Here, we present in Fig. B2 that the phenomenon described is quite general and can also be observed if **B** is already polymorphic. Subsequent substitutions at the **A** locus can bypass the local stability of the S_B equilibrium in the $\{p_A, p_B\}$ plane.

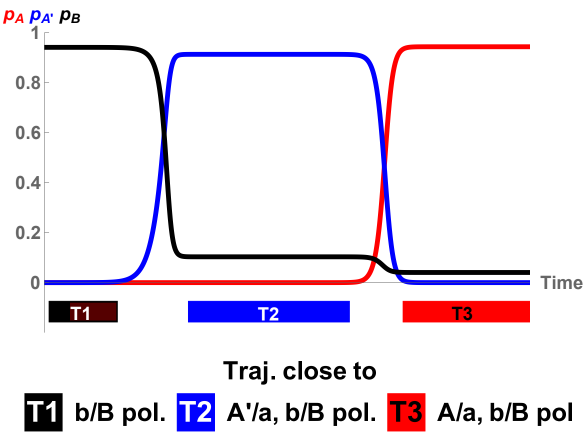

(a) Frequency of the different alleles

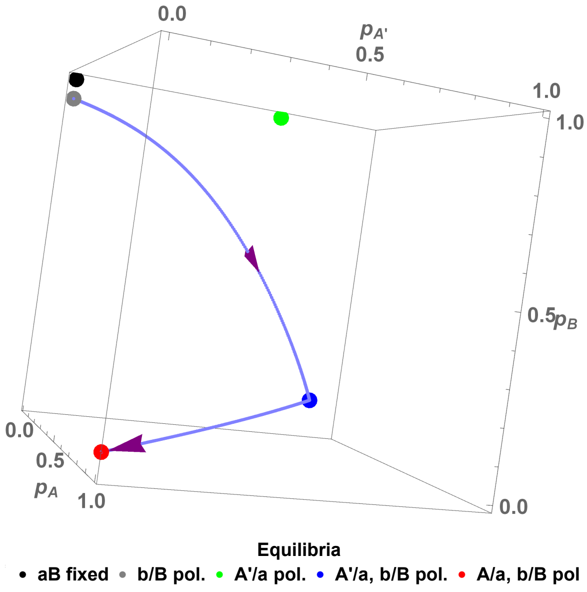

(b) Trajectories in the $\{p_A, p_B, p_{A'}\}$ space

Figure B2: **Evolutionary trajectory with A' as transient state.**

a) Frequencies of derived alleles **B** (black), **A** (red), and **A'** (blue) as a function of time. At t=0, the population is polymorphic at locus **B**, with both the allele **A** and **A'** present at an extremely low frequency ($\approx 10^{-6}$). Colored blocks T1-T3 indicate when the population is close to an equilibrium, with the color matching the corresponding equilibrium. b) We represent the same trajectory in the $\{p_A, p_B, p_{A'}\}$ space. Dots correspond to the different equilibria. Arrows indicate the evolutionary trajectory. Parameters used are: $\frac{\beta}{\alpha} = -\frac{17}{300}$, $\frac{\alpha'}{\alpha} = \frac{2}{3}$, $\frac{\epsilon_{AB}}{\alpha} = -\frac{4}{3}$, $\frac{\epsilon_{A'B}}{\alpha} = -\frac{151}{300}$, $\frac{m}{\alpha} = \frac{4}{75}$.

B 5 Does invasion of allele A' always displace allele A?

We have shown earlier that **A** and **A'** cannot coexist. In addition, we have demonstrated that replacement is a possible but not a guaranteed outcome of a successful invasion of allele **A'** (in contrast to a single-locus case). Here, we aim to prove that invasion of allele **A'** always excludes allele **A**; if **A'** invades, allele **A** is always lost.

To do this, we need to show that invasion of S_{AB}^{stable} by allele **A'** does not generate a trajectory that ends in an equilibrium where allele **A** is still present. There is only two equilibria that can be stable with allele **A** present: S_{AB}^{stable} and S_A. Per definition, the first one is not an option as we are investigating cases where it is being invaded and therefore unstable. Therefore, if S_A is either not present or unstable, invasion always leads to displacement of allele **A**.

This is the case if:

- $S_{A'}$ is stable. Indeed, S_A and $S_{A'}$ can not be stable at the same time. Indeed, the eigenvalue corresponding to the invasion of S_A by **A'** is identical to a -1 factor to the eigenvalues corresponding to the invasion of S'_A by **A**, therefore preventing co-stability of the two equilibria.

- S_B or fixation of the continental haplotype is stable. Indeed, the stability of all three equilibria S_A, S_B and fixation of the continental type is mutually exclusive; only one of them can be stable.

- $S_{A'B}^{stable}$ is globally stable.

Unfortunately, for a specific range of parameter, we observe that S_A can be locally stable when **A'** can successfully invade S_{AB}^{stable} (which is locally stable in the $\{p_A, p_B\}$ plane). Numerical estimations suggest that the trajectories following invasion of S_{AB}^{stable} by **A'** leads to the $S_{A'B}^{stable}$. We never observe one ending in S_A, suggesting that invasion indeed leads to the loss of allele **A**.

B 6 Replacement of allele A by A' leads to stronger genetic barrier

In the previous sections, we focus on the consequences of the invasion of allele **A'**. Now, we focus on how the genetic barrier is affected assuming that **A'** has successfully replaced allele **A**.

We further investigate here the reasons for the strengthening of the genetic barrier by allele **A'**. We focus mainly on the case illustrated in Fig. 1(a), where $\alpha' > \alpha$. If epistasis is unchanged,

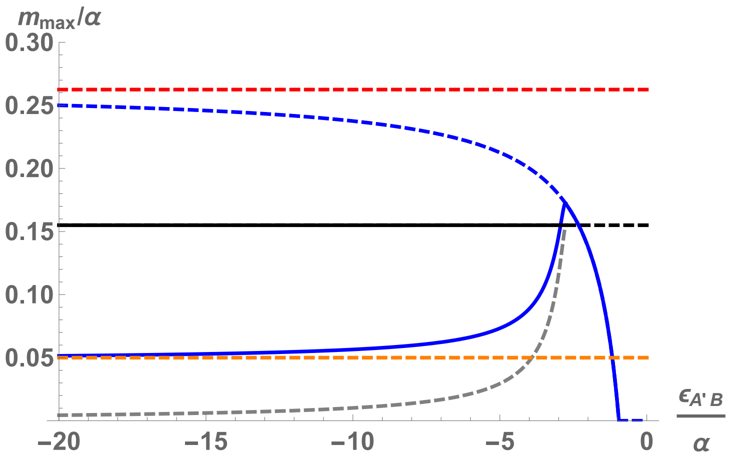

Figure B3: **Genetic barrier in the two-locus three-alleles model**

The x-axis corresponds to the epistasis between **B** and the new allele **A'**. The y-axis corresponds to migration. The black and blue lines corresponds to the genetic barrier with allele **A** and **A'**, m^{Ab}_{max} and $m^{A'b}_{max}$, respectively. The dashed lines (black and blue) correspond to the genetic barrier in the absence of the third allele and the solid line to the genetic barrier in presence of the third allele. If the dashed line is not visible, it is below the solid line. The dashed gray line corresponds to the minimal migration rate for stability of the S^{stable}_{AB} equilibrium in the presence of **A'**. The red and orange dashed lines correspond to the asymptotic behavior of $m^{A'b}_{max}$ for the $S^{stable}_{A'B}$ equilibrium in presence ($\alpha' - \alpha$, orange) and absence ($\alpha'/4$, red) of allele **A**. This figure is obtained for the exact same parameters as Fig. 1(a).

then the genetic barrier is strengthened, as selection against migrants increases. Because both selection against hybrids and selection against migrants participate to the barrier, this slight increase in selection against migrants allows a slight corresponding decrease in selection against hybrids, i.e. the genetic barrier is also strengthened for epistasis slightly weaker than the original ϵ_{AB}.

When β is positive and close to α', we know that in a two-locus two-alleles model, $m^{A'b}_{max}$ is a decreasing function of $\epsilon_{A'B}$, with an asymptote in $\alpha'/4$ if $\epsilon_{A'B} \to -\infty$. However, in the two-locus three-alleles model, as epistasis gets stronger, the $S^{stable}_{A'B}$ equilibrium gets more and more vulnerable to invasion by **A**. Indeed an increase in negative epistasis both decreases the marginal fitness of **A'** (it pays a stronger hybrid cost) and increases the marginal fitness of **A** (as the frequency of **B** is decreased due to the stronger epistasis). Therefore, the genetic barrier is only strengthened for a small range of $\epsilon_{A'B} < \epsilon_{AB}$, until invasion of the $S^{stable}_{A'B}$ equilibrium by **A** prevents any further strengthening. Under this model, $m^{A'b}_{max}$ admits an asymptote $\alpha' - \alpha$ as $\epsilon_{A'B} \to -\infty$.

In this situation, the strongest barrier is then formed for the stronger epistasis that still does not allow invasion of **A** at any migration rate : $\epsilon_{A'B} = \epsilon_{AB}\frac{2\alpha'-\alpha}{\alpha}$. Since $\alpha' > \alpha$, this only happens for $\epsilon_{A'B} < \epsilon_{AB}$, therefore guaranteeing a small range of epistasis values where the

barrier is also strengthened.

As a conclusion, in the situation illustrated here ($\beta > 0$), if the new allele **A'** is slightly better than **A**, then it will only strengthen the barrier if the change in epistasis is also small.

B 7 A third allele at locus B on the continent

So far, we investigate in detail the consequences of a third allele at the A locus. We now assume that a new allele **B'** appears on the continent and replaces the **B** allele. We investigate when **B'** will strengthen, weaken or destroy the genetic barrier formed by the A and B loci. If epistasis is unchanged, the pattern is clear: the genetic barrier is strengthened if and only if selection against migrants increases as illustrated on Fig. B4 (vertical dashed line on each panel). If selection is unchanged, we observe the following rule: if **B** is deleterious enough on the island (Fig. B4 first line) then reducing the epistasis strengthens the barrier; if **B** is advantageous, increasing epistasis strengthens the barrier (Fig. B4 last two lines). Lastly, if **B** is only slightly deleterious on the island, the genetic barrier reaches a maximum for an intermediate (not too large) value of epistasis (see Bank et al. [2012]). In that case, slightly weaker or larger negative epistasis may strengthen the barrier depending on how far the original epistasis is from this optimal value, (weaker epistasis: Fig. B4 second line,$\frac{\epsilon_{AB}}{\alpha} = -.9$ or -1.1 or stronger epistasis: Fig. B4 second line,$\frac{\epsilon_{AB}}{\alpha} = -.25$).

We can then deduce the following general rule: the genetic barrier is strengthened if **B'** is more deleterious than **B** on the island and the epistasis does not deviate too much from the original value, ϵ_{AB} (area around the lower part of vertical dashed line is always blue on (Fig. B4). If $\beta' < 0$, then strengthening of the genetic barrier is more likely with weaker epistasis, whereas if $\beta' > 0$, most of strengthening happens if the incompatibility gets stronger. If **B'** is much more deleterious than **B** on the island, the barrier will be strengthened regardless of epistasis.(the strong selection against migrants releases **A** from an unnecessary hybrid cost).

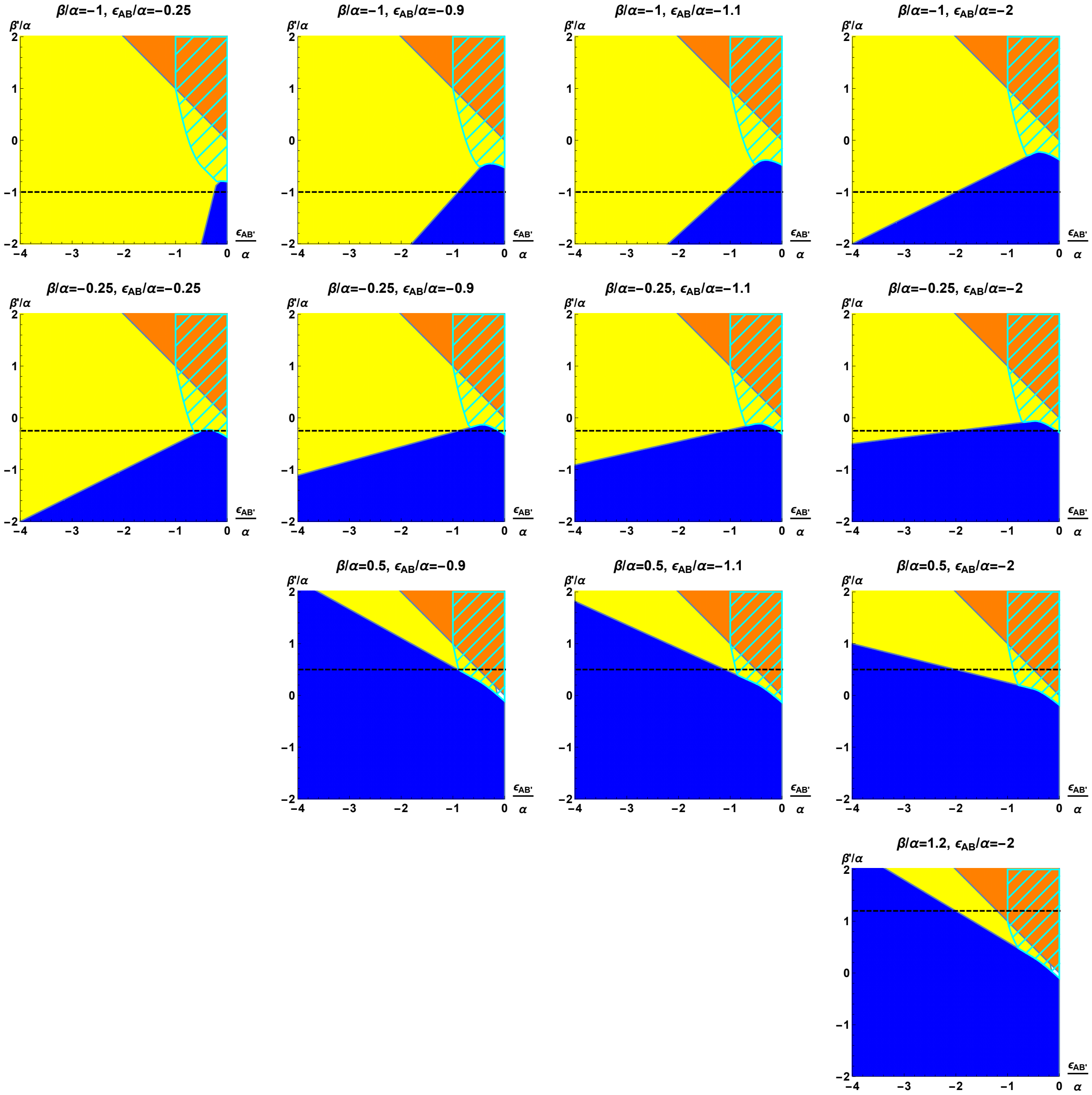

Figure B4: **A third allele at locus B on the continent**

We represent the consequence of substituting allele **B** by **B'** on the continent. The x-axis corresponds to the epistasis coefficient between **A** and B', $\epsilon_{AB'}$; the y-axis corresponds to the new allele selective advantage on the island β'. Blue indicates that the barrier is strengthened, yellow that the new DMI formed exists only for weaker migration rate and orange that the barrier is completely destroyed. The dashed lines indicated the values of allele **B**. The dashed cyan area indicates that if the DMI is lost, then **A** is still maintained polymorphic.

B 8 Diploid case

Most our analysis focus on the haploid case, as it far more easy to track this case analytically. We now consider a diploid population. The corresponding fitness table corresponding is given in Table B1. For a codominant model, we need the conditions on epistasis given in equation (B10a). With this implementation, the dynamics of the diploid model simplify to the haploid dynamics.

For the recessive model, we have two options: either we see the two-locus three-alleles model as a specific case of three locus model and then use the same implementation, or we assume that if **A** and **A'** are both present, then only the weaker incompatibility is expressed. The difference reduces mainly to whether or not the genotype **AA'Bb** expresses any epistasis. In the first case, we need the conditions given in (B10b) whereas in the second case we use the substitutions given in (B10c).

$$\eta_{AB} = \frac{\epsilon_{AB}}{2}, \eta_{A'B} = \frac{\epsilon_{A'B}}{2}, \theta_{AB} = 2\epsilon_{AB}, \theta_{A'B} = 2\epsilon_{A'B}, \epsilon_{AA'B} = \frac{\epsilon_{AB} + \epsilon_{A'B}}{2} \text{ and } \theta_{AA'B} = \epsilon_{AB} + \epsilon_{A'B} \tag{B10a}$$

$$\eta_{AB} = 0, \eta_{A'B} = 0, \theta_{AB} = 2\epsilon_{AB}, \theta_{A'B} = 2\epsilon_{A'B}, \epsilon_{AA'B} = 0 \text{ and } \theta_{AA'B} = \epsilon_{AB} + \epsilon_{A'B} \tag{B10b}$$

$$\eta_{AB} = 0, \eta_{A'B} = 0, \theta_{AB} = 2\epsilon_{AB}, \theta_{A'B} = 2\epsilon_{A'B}, \epsilon_{AA'B} = \text{Min}\left[|\epsilon_{AB}|, |\epsilon_{A'B}|\right] \text{ and } \theta_{AA'B} = 2\text{Min}\left[|\epsilon_{AB}|, |\epsilon_{A'B}|\right] \tag{B10c}$$

Both recessive models behave qualitatively differently when compared to the haploid model. Indeed, they both admit an internal stable equilibrium with both **A** and **A'** polymorphic. For the first case, the invading allele does not suffer at all from the incompatibly when rare, making invasion really easy. In the second case (illustrated in Fig. B5), if the new allele has a weaker selective advantage, it will still invade because of smaller hybrid cost. However, when the allele (**A** here) that generates the strongest hybrid cost is rare, it only uses the incompatibility of

	aB	AB	$A'B$	ab	Ab	$A'b$
aB	2β	$\alpha + 2\beta + \epsilon_{AB}$	$\alpha' + 2\beta + \epsilon_{A'B}$	β	$\alpha + \beta + \eta_{AB}$	$\alpha' + \beta + \eta_{A'B}$
AB		$2\alpha + 2\beta + \theta_{AB}$	$\alpha + \alpha' + 2\beta + \theta_{AA'B}$	$\alpha + \beta + \eta_{AB}$	$2\alpha + \beta + \epsilon_{AB}$	$\alpha + \alpha' + \beta + \epsilon_{AA'B}$
$A'B$			$2\alpha' + 2\beta + \theta_{A'B}$	$\alpha' + \beta + \eta_{A'B}$	$\alpha + \alpha' + \beta + \epsilon_{AA'B}$	$2\alpha' + \beta + \epsilon_{A'B}$
ab				0	α	α'
Ab					2α	$\alpha + \alpha'$
$A'b$						$2\alpha'$

Table B1: **Fitness table for the two-locus three-alleles model in a diploid population**

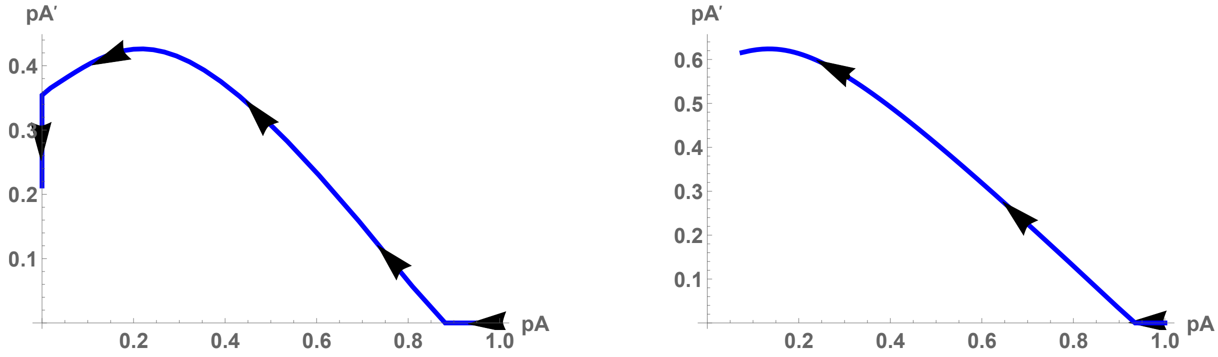

(a) $\frac{\beta}{\alpha} = -0.5$, $\frac{\alpha'}{\alpha} = 0.95$, $\frac{\epsilon_{AB}}{\alpha} = -1.75$ and $\frac{\epsilon_{A'B}}{\alpha} = -1.25$

(b) $\frac{\beta}{\alpha} = 0.5$, $\frac{\alpha'}{\alpha} = 0.95$, $\frac{\epsilon_{AB}}{\alpha} = -1.75$ and $\frac{\epsilon_{A'B}}{\alpha} = -1.25$

Figure B5: **Trajectories of the stable equilibrium as migration (m) increases in a diploid recessive model**

We represent the trajectories of the stable equilibrium (fixation of **Ab** at m=0) as m increases in the $\{p_A, p'_A\}$ plane. The black arrows indicates how the equilibrium evolves as m increases. Here we assume that **AA'Bb** expresses epistasis (equation (B10c)).

the common allele and therefore, because of its selective advantage can also invade. This is only possible if the allele with the strongest selective advantage is also the one that is most incompatible with **B** (for example $\alpha > \alpha'$ and $\epsilon_{AB} < \epsilon_{A'B}$). In Fig B5(a), at $m = 0$ we start from fixation of the **Ab** haplotype. As m increases, the frequency of **B** increases until invasion of **A'** is possible leading to a stable equilibrium with alleles **a**, **A** and **A'** at locus $\mathcal{A}$ and **b** and **B** at locus $\mathcal{B}$. As m keeps increasing, allele **B** starts to be "too frequent" and allele **A** is then lost. We return to the usual two-locus two-alleles equilibrium. This equilibrium finally vanishes as m keeps increasing. When compared to the haploid case, the internal equilibrium enters here through the stable equilibrium (S_{AB}^{stable}) instead of the unstable one ($S_{A'B}^{stable}$, as defined in section B 1, stable refers to the stability in the two-locus two alleles model). In Fig B5(b), the trajectories are identical at the beginning. However, the internal 3-alleles equilibrium vanishes before it can leave the state space through the $S_{A'B}^{stable}$ equilibrium.

With such an implementation and assuming $\epsilon_{AB} < \epsilon_{A'B}$, **AA'** is overdominant if its marginal fitness is the largest among all combination of allele at locus $\mathcal{A}$:

$$\epsilon_{A'B} > \frac{\alpha}{p_B(p_B - 2)} \text{ and } \mathrm{Max}[\alpha - 2p_B\epsilon_{A'B}, p_B(p_B\epsilon_{AB} - 2\epsilon_{A'B}), \alpha + 2p_B(\epsilon_{AB} - \epsilon_{A'B})] < \alpha' < \alpha \tag{B11}$$

For both cases illustrated in Fig. B5, overdominance is always observed when the 3-alleles internal equilibrium exists. However, this not a sufficient condition. Indeed, when the stability switches from the internal equilibrium ($S_{AA'B}$) to the $S_{A'B}^{stable}$ equilibrium, **AA'** stays overdominant. In both cases, the genetic barrier in the two-locus two-alleles model corresponds to the

collision of the 2 internal equilibriums S_{AB}^{stable} with $S_{AB}^{unstable}$.

References

C. Bank, R. Bürger, and J. Hermisson. The Limits to Parapatric Speciation: Dobzhansky–Muller Incompatibilities in a Continent–Island Model. *Genetics*, 191(3):845–863, 2012.

T. Nagylaki and Y. Lou. Patterns of multiallelic polymorphism maintained by migration and selection. *Theoretical Population Biology*, 59(4):297–313, 2001.
