# Supplementary material for: The Limits to Parapatric Speciation II: Strengthening a Preexisting Genetic Barrier to Gene Flow in Parapatry
Source: Genetics. 2018 Feb 28;209(1):241–54. doi: 10.1534/genetics.117.300652 (PMC5937195; doi:10.1534/genetics.117.300652)
Supplement: Supplementary file 3 [file 241FileS3.pdf]

Supplementary File C: Extension of the genetic barrier

Alexandre Blanckaert *[1,2] and Joachim Hermisson[1,3]

[1]Department of Mathematics, University of Vienna, 1090 Vienna, Austria

[2]Instituto Gulbenkian de Ciência, 2780-156 Oeiras, Portugal

[3]Mathematics and Biosciences Group, Max F. Perutz Laboratories, 1030 Vienna, Austria

February 28, 2018

C 1 Extension of the genetic barrier

In this section, we investigate the impact of a new mutation, **C**, appearing at a locus in loose linkage with any component of the previous genetic barrier.

C 1.1 Extension of the barrier from one to 2 loci

C 1.1.1 A single-locus barrier can be formed

We assume that **C** appears on the island. It interacts either with the island adaptation or the continental one. In the first case, A is the polymorphic locus and in the second one, it is B. This is in fact the same problem with just two different parametrizations. Indeed, with 2 loci and 2 alleles, the system is fully parametrized with 2 selection coefficients and one epistatic one, as it generates a different fitness for each haplotype. We therefore focus on the interactions between the B and C loci. Table C1 provides the two parametrizations and the link between them. Equation (C1) gives the minimal condition on the selective advantage of **C** to strengthen the genetic barrier. In addition, equation (C2) gives the necessary (γ_{nec}) and sufficient (γ_{suf}) conditions for the mutation **C** to invade on the island, with locus **B** being polymorphic, ie $0 \leq m \leq -\beta$. These two conditions are obtained at $m = 0$ and $m = m^b_{max}$. If the fitness of haplotype **bC** is smaller than the fitness of haplotype **BC**, then **B** impedes the invasion of **C**

*ablanckaert@igc.gulbenkian.pt

on the island. The necessary condition is therefore obtained for $m = 0$ and the sufficient one for $m = m^b_{max}$. If **B** helps **C** to invade, then the roles are reversed: the necessary condition is obtained for $m = m^b_{max}$ and the sufficient one for $m = 0$.

$$
\begin{array}{ll}
\text{If } \beta \leq \epsilon_{BC} & \gamma \geq -(\beta + \epsilon_{BC}) \\
\text{If } \beta \geq \epsilon_{BC} & \gamma \geq -\frac{4\beta\epsilon_{BC}}{\beta+\epsilon_{BC}}
\end{array}
\tag{C1}
$$

$$
\begin{aligned}
&\text{if } w(bC) \leq w(BC) : \begin{cases} \gamma_{nec} &= 0 \\ \gamma_{suf} &= -(\beta + \epsilon_{BC}) \end{cases} \\
&\text{if } w(bC) \geq w(BC) : \begin{cases} \gamma_{nec} &= -(\beta + \epsilon_{BC}) \\ \gamma_{suf} &= 0 \end{cases}
\end{aligned}
\tag{C2}
$$

1st option	**ac**	**Ac**	**aC**	**AC**
	0	α	γ	$\alpha + \gamma + \epsilon_{AC}$
**2nd option**	**Bc**	**bc**	**BC**	**bC**
	0	$-\beta'$	$\gamma' + \epsilon'_{BC}$	$\gamma' - \beta'$

Table C1: **Fitness table and equivalence between an island adaptation C interacting with either an island adaptation, A, or a continental adaptation B**

We choose the continental haplotype to have a fitness of zero on the island. Then, the two parametrization are equivalent with the following change $\alpha = -\beta', \gamma = \gamma' + \epsilon'_{BC}$ and $\epsilon_{AC} = -\epsilon'_{BC}$.

First, we assume that **C** interacts with an immigrating **B** allele. If the barrier is strengthened, then the new barrier m^b_{max} is given either by fixation of allele **B** (transcritical bifurcation, Fig. C1(a)) despite the presence of **C** or by loosing the **C** allele because the hybrid cost becomes too large (saddle-node bifurcation, Fig. C1(b)). Indeed, if **C** is lost due to its incapacity to withstand migration (transcritical bifurcation), then **B** is still polymorphic when this happens, i.e. $m^{bC}_{max} < m^b_{max,0}$. Therefore, strengthening can never happen in this case.

This is further illustrated in Fig. C1, where we represent the frequencies of alleles **B** and **C**. The barrier is strengthened for any ϵ_{BC} between the vertical gray line and 0. For Fig. C1(a), at $m = m^b_{max}$, allele **B** fixes on the island and **C** stays polymorphic. This corresponds to the blue area above the thick black dashed line on Fig. 3. For Fig. C1(b), at $m = m^b_{max}$, both **B** and **C** stay polymorphic for strong enough ϵ_{BC} (blue area below the thick black dashed line on Fig. 3). Then as epistasis weakens, **B** fixes on the island and **C** stays polymorphic. This corresponds to the blue area above the thick black dashed line on Fig. 3.

Now, we assume that **C** interacts with a locally adapted **A** allele, Fig C2. The barrier is

strengthened as long as there is some synergy (positive epistasis) between the two mutations. As we can see, the barrier can be strengthened for parameters where neither **A** nor **C** can exist on their own on the island (for example, $\gamma < 0$ and **A** exists on the island for $m > \alpha$). The epistatic interaction boosts the marginal fitness of both alleles, allowing them to withstand stronger gene flow, until a point where both vanish (the 2 internal equilibria leave the $\{p_A, p_C\}$ plane). Especially, if both **A** and **C** have the same direct effect, they can reinforce each other if epistasis between **A** and **C** is positive enough, see Fig. C2(a). Indeed, when m is close to m^A_{max} $= m^C_{max}$, the frequencies of both alleles **A** and **C** are still high because of the extra fitness boost they provide to each other through epistasis. In addition, this effect is strong enough to allow a deleterious **C** mutation to invade and establish on the island, Fig. C2.

We now consider that the **C** allele appears on the continent and interacts with the island adaptation, the **A** allele. In that case, as illustrated in Fig. C3, negative epistasis will weaken or even destroy the barrier (orange and red area). Indeed, the **A** allele now suffers from forming some unfit hybrids, even if the immigrating individuals are extremely unfit. If epistasis is positive, the barrier is always strengthened, either through fixation of allele **C** on the island (green and cyan area) or through a two-locus polymorphism (blue area).

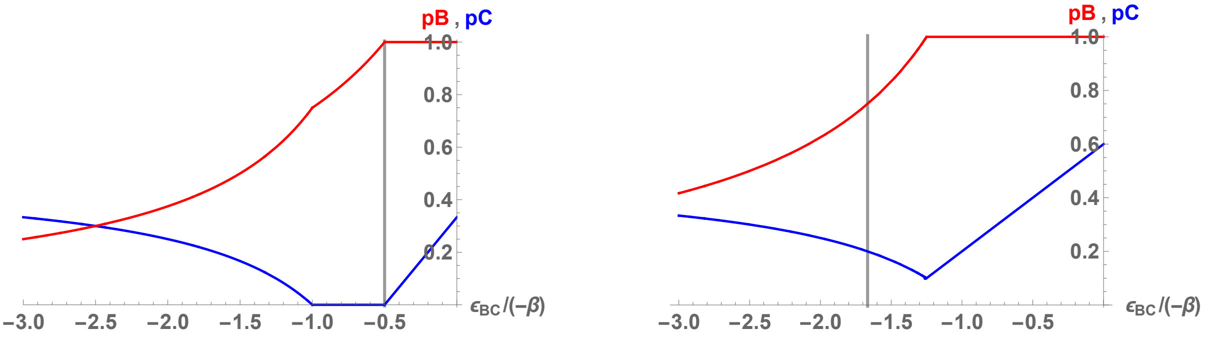

(a) $\gamma = -1.5\beta$

(b) $\gamma = -2.5\beta$

Figure C1: **Frequencies of the derived alleles at** $m = m^b_{max}$

The x-axis corresponds to the epistasis between **B** and **C**. The y-axis corresponds the frequencies of allele **B** (red) and **C** (blue) at $m = m^{AC}_{max}$. The gray vertical line corresponds to the lower limit of ϵ_{BC} for strengthening of the barrier (the two-locus barrier is stronger than the single-locus barrier between this value and 0).

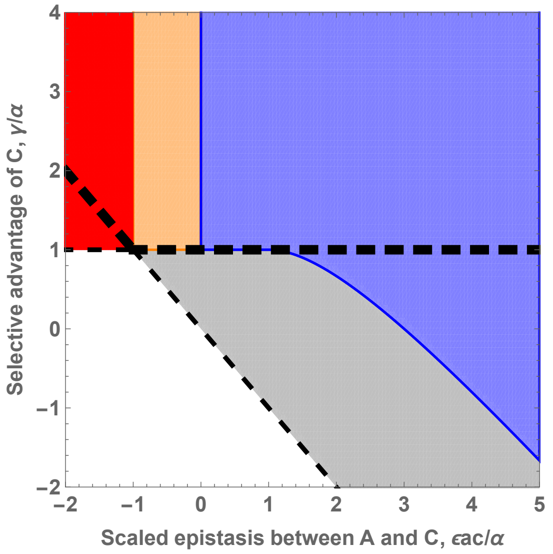

(a) **C** interacts with **A**

Figure C2: **C strengthens the genetic barrier formed by a single polymorphic locus**

The x-axis shows the strength of epistasis between **A** and **C**. The y-axis shows the selective advantage of new allele **C**. The background color indicates the consequence of the invasion of allele **C** on the genetic barrier at the **A** locus: gray the genetic barrier remains unchanged, blue the genetic barrier is strengthened, orange the genetic barrier is weakened and red the polymorphism at locus **A** is lost. In addition, the solid black line gives the necessary condition for invasion of allele **C** on the island. Below this bound invasion is always impossible. The black dashed line gives the sufficient condition for invasion. Above this bound, allele **C** can always invade, regardless of the migration rate (provided the polymorphism at the **A** locus still exists).

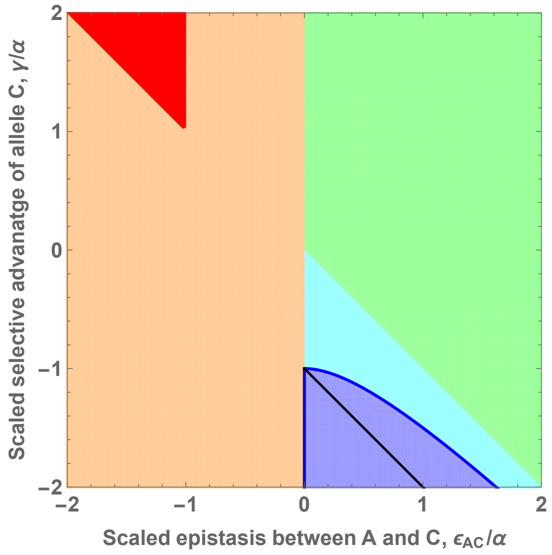

Figure C3: **$\mathcal{C}$ strengthens the genetic barrier formed by a single polymorphic locus; allele C appears on the continent**

The x-axis corresponds to the scaled epistasis between $\mathcal{A}$ and $\mathcal{C}$. The y-axis corresponds to the scaled selective advantage of **C**. The background color indicates the consequence of the invasion of allele **C** on the genetic barrier at the $\mathcal{A}$ locus: red, allele **A** is lost, orange the genetic barrier is weakened, both green and light blue indicate that the barrier is strengthened through fixation of allele **C** on the island and dark blue indicates the barrier is strengthened even if $\mathcal{C}$ remains polymorphic. The distinction between green and light blue has to do with the fate of the polymorphism at low migration rate: for green, **C** can always fix, for light blue **C** fixes only if migration is strong enough. While the dark blue area indicates that the genetic barrier is strengthened with both loci remaining polymorphic, one of them will eventually fix its continental allele: above the black line allele **C** will fix first, below allele **A** is lost first.

C 1.1.2 A single-locus barrier is impossible ($\beta > 0$).

In this paragraph, we assume that **B** is advantageous on the island. Therefore, there is no initial single-locus genetic barrier, $m^b_{max,0} = 0$. Following a successful invasion of allele **A**, the expression of the strength of the genetic barrier is given by equation (C3).

$$\begin{array}{lll} \text{If } -\infty < \epsilon_{AB} < \text{Min}[-\beta, -\alpha/2] & m^b_{max} = \frac{\alpha(\epsilon_{AB}+\beta)}{4\epsilon_{AB}} \\ \text{If } -\frac{\alpha}{2} < \epsilon_{AB} < -\beta & m^b_{max} = -\frac{(\alpha+\epsilon_{AB})(\epsilon_{AB}+\beta)}{\alpha} \end{array} \tag{C3}$$

We define α_{min} as the minimal selective advantage of a new allele **A** required to form a genetic barrier. From equation (C3), one can define necessary conditions for a new **A** mutation to form such a barrier.

- if $\beta > \frac{\alpha}{2}$ or $\epsilon_{AB} < -\beta$, then the genetic barrier is lost due to fixing both alleles **A** and **B** together (first expression in (C3)). Therefore, we obtain $\alpha_{min} = 4m\frac{\epsilon_{AB}}{\epsilon_{AB}+\beta}$ and $\alpha > 4m$ is a necessary condition (respectively the red and black dashed line on Fig. C4).
- if $\beta < -\epsilon_{AB} < \frac{\alpha}{2}$, then the genetic barrier is lost due to fixing allele **B** (second expression in (C3)). Then if $m < -(\beta + \epsilon_{AB})$, $\alpha_{min} = -\epsilon_{AB}\frac{\beta+\epsilon_{AB}}{m+\beta+\epsilon_{AB}}$ (blue line on Fig. C4) is a necessary condition. m^b_{max} admits $\frac{\alpha}{4}$ as a maximum in $\epsilon_{AB} = -\frac{\alpha+\beta}{2}$, therefore we can deduce a simpler necessary condition from this expression: $\alpha > 4m$. However, $-\frac{\alpha+\beta}{2}$ is never in the range of definition of m^b_{max}, and m^b_{max} is a decreasing function of ϵ_{AB} over $-\beta > \epsilon_{AB} > -\frac{\alpha}{2}$. Therefore, a maximum is reached in $\epsilon_{AB} = -\frac{\alpha}{2}$, $m^b_{max} \leq \frac{\alpha-2\beta}{4}$ leading to $\alpha > 4m + 2\beta$ (blue dashed line on Fig. C4). This last expression is a more precise necessary condition.

As a conclusion $\alpha > 4m$ is a always a necessary condition (it is also sufficient for lethal incompatibilities) to observe a genetic barrier against swamping.

In addition, if $\alpha/2 > \beta$ and $\epsilon_{AB} < -\alpha/2$, then $\alpha > 4m + 2\beta$ is also sufficient condition. (green dashed line on Fig. C4. Indeed, m^b_{max} is a decreasing function of ϵ_{AB}, therefore $m^b_{max} > m^b_{max}(\epsilon_{AB} = -\alpha/2) = \frac{\alpha-2\beta}{4}$.

Last, $\epsilon_{AB} < -\beta$ is a known necessary condition (see Bank et al. [2012]). We are here a bit more precise. We have shown before that $\epsilon_{AB} < -\beta - m$ is a necessary condition if the barrier disappears through fixation of allele **B**. If the barrier is lost through fixation of both allele **a** and **B**, then we have $\epsilon_{AB} < -2m - \beta$.

Therefore, a simple necessary condition for a **A** mutation to maintain a barrier to swamping is : $\alpha > 4m$ and $\epsilon_{AB} < -\beta - m$.

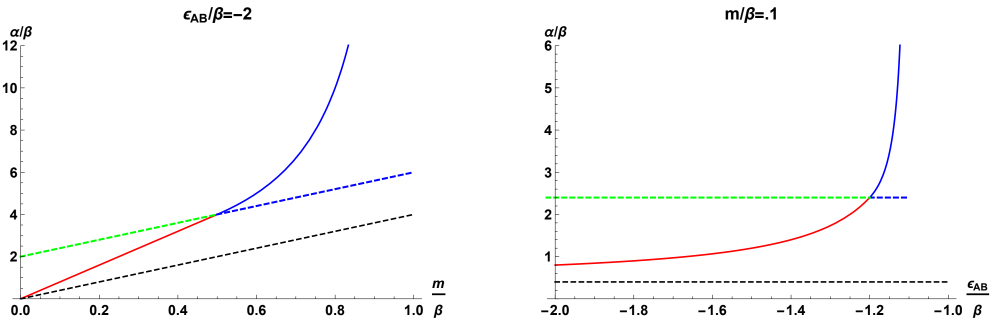

Figure C4: **Minimal selective advantage of an A mutation to form a two-locus genetic barrier**

The x-axis corresponds either to scaled migration (left panel) or scaled epistasis (right panel). The y-axis corresponds to the scaled minimum selective advantage of **A**, α, to ensure the existence of a genetic barrier. The blue line corresponds to α_{min} if the barrier is lost due to fixation of allele **B** and the red one due to fixation of both **a** and **B**. The different dashed lines correspond to necessary conditions (black ($4m$) and blue ($4m + 2\beta$) lines) and the green line ($4m + 2\beta$) to a sufficient condition.

C 1.2 Extension of the barrier from 2 to 3 loci

A new allele **C**, appearing on the island, at a locus in loose linkage with the other loci, may strengthen the genetic barrier only if it is not swamped first and alone. This in particular implies that $\gamma > m^{Ab}_{max}$, as **C** needs first to be able to withstand migration before paying any hybrid cost.

The two-locus barrier can be given by three different events: the loss of allele, **A** (transcritical bifurcation), fixation of allele **B** (transcritical bifurcation) or loosing the internal equilibrium through a saddle-node bifurcation. If $m^{Ab}_{max,0}$ is given by the fixation of allele **B** (through the transcritical bifurcation), then $m^{Ab}_{max,0} > -\beta$ and therefore $\gamma > -\beta$ is a necessary condition for the strengthening of the barrier.

$m^{Ab}_{max,0}$ is given by the loss of allele **A** (through the transcritical bifurcation). It happens if $\beta < \epsilon_{AB} < -(\alpha + \beta)$. Then $m^{Ab}_{max,0}$ is an increasing function of ϵ_{AB} and therefore, its minimum is reached in $\epsilon_{AB} = \beta$, i.e. $m^{Ab}_{max,0} \geq \frac{\alpha}{2}$. Therefore, $\gamma > \frac{\alpha}{2}$ is a necessary condition in this case.

If $m^{Ab}_{max,0}$ is given by the saddle-node bifurcation, then $m^{Ab}_{max,0}$ is an increasing function of ϵ_{AB} if $\beta < 0$. Therefore $m^{Ab}_{max,0} > \frac{\alpha}{4}$, leading to $\gamma > \frac{\alpha}{4}$. If $\beta > 0$, there is no necessary condition on γ (except the obvious $\gamma > 0$) as the minimum of m^{Ab}_{max} is 0 in that case.

Therefore, if **B** is deleterious on the island, there is always a necessary condition on allele **C**, for a new mutation to potentially strengthen the barrier:

$$
\begin{cases}
-\infty < \epsilon_{AB} < -\text{Max}\left[\frac{\alpha}{2}, -\beta\right] & \gamma > \frac{\alpha}{4} \\
\beta < \epsilon_{AB} < -(\alpha+\beta) & \gamma > \frac{\alpha}{2} \\
-\text{Min}\left[\frac{\alpha}{2}, \alpha+\beta\right] < \epsilon_{AB} < 0 & \gamma > -\beta
\end{cases} \tag{C4}
$$

In the main manuscript, we mainly focus on the case **B** advantageous on the island, as the other one is quit similar to the $1 \to 2$ transition. Here, we discuss the other case, i.e. allele **B** is deleterious on the island. Therefore, a single-locus genetic barrier, $m^b_{max,0} = -\beta$ exists. In addition, we also consider that allele **C** interacts with a different continent island. Instead of repressing allele **B**, it impacts only indirectly on the genetic barrier by interacting with allele **a**. While this case is not covered in the general model, it can easily be infer by slightly altering the fitness table A1.

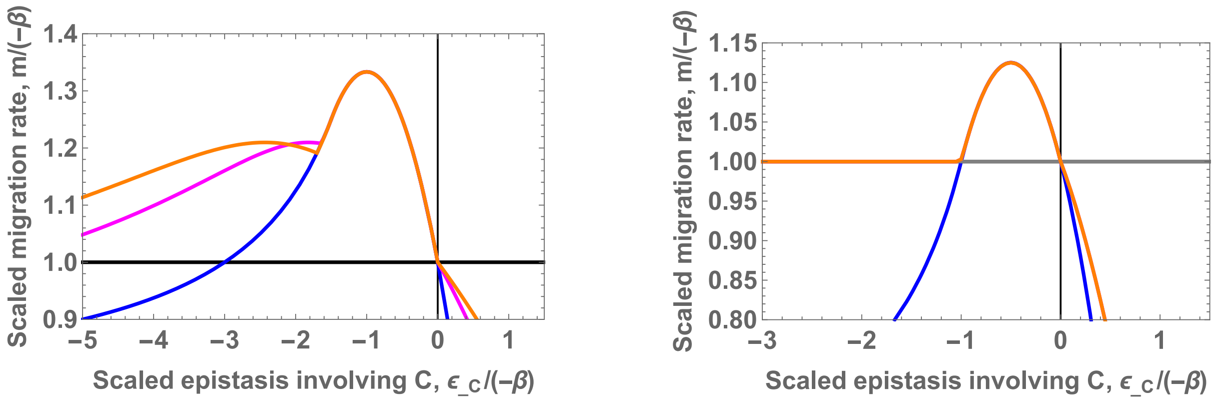

(a) $\frac{\alpha}{-\beta} = 1.2$, $\frac{\gamma}{-\beta} = 3$ and $\frac{\epsilon_{AB}}{-\beta} = -3$

(b) $\frac{\alpha}{-\beta} = 1.5$, $\frac{\gamma}{-\beta} = 2$ and $\frac{\epsilon_{AB}}{-\beta} = 0.5$

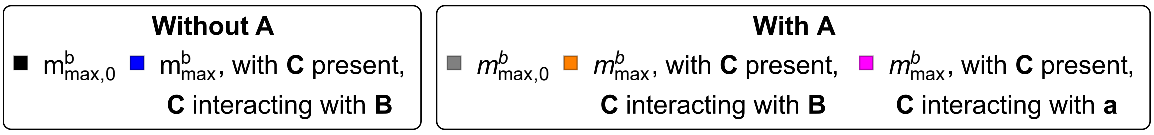

Figure C5: **Genetic barrier to swamping for all loci in loose linkage**

The x-axis corresponds to the epistasis between **C** and its interacting allele. The y-axis corresponds to m_{max}, the genetic barrier for local stability, i.e. resistance to swamping. The black line corresponds to the genetic barrier before the apparition of **C**. To make comparison easier, $m^b_{max,0} = m^{Ab}_{max,0}$. The blue line corresponds to m^b_{max} and both the orange and magenta lines to m^{Ab}_{max} with **C** interacting with allele **B** (orange) or allele **a** (magenta).

Fig. C5 illustrates the impact of a third mutation on m^b_{max}. We assume that **A** is also polymorphic (otherwise, the case has already been described in the previous section) and therefore $m^b_{max,0}$ is given by $m^{Ab}_{max,0}$. To allow for easier (visual) comparison between the different scenarios, we also choose α and ϵ_{AB} such that $m^b_{max,0} = m^{Ab}_{max,0}$ (black line). Therefore, we can compare the impact of **C** as a second mutation versus **C** as a third mutation. We represent the impact of the new mutation **C** on the genetic barrier, for the "$1 \to 2$ transition" in blue

(m^b_{max}) and the "2 → 3 transition" in orange (m^{Ab}_{max}, epistasis with allele **B**) or magenta (m^{Ab}_{max}, epistasis with allele **a**).

First, the results obtained for the "2 → 3 transition" are very similar to the "1 → 2 transition". Indeed, the genetic barrier is only strengthened for negative epistasis between the new island adaptation and its interacting continental allele. Secondly, γ has to be larger than m^{Ab}_{max} as the **C** mutation needs to be able to resist gene flow up to $m^{Ab}_{max,0}$ (otherwise it will be swamped before being able to strengthen anything) plus the hybrid cost. Lastly, if γ is only slightly larger than $m^{Ab}_{max,0}$, the barrier is strengthened only for weak negative epistasis; if γ is large enough ($> 4m^b_{max,0}$ for the 1 → 2 transition and $> 4\ m^{Ab}_{max,0}$ for 2 → 3), the barrier is always strengthened for negative epistasis, even for lethal incompatibilities.

Since we focus on preventing the fixation of allele **B** on the island, the presence of allele **A** is not necessary to the existence of the genetic barrier. Therefore, the impact of **A** is studied only if it strengthens the genetic barrier in the first place, i.e $m^{Ab}_{max} > m^b_{max} = -\beta$. Now, for **C** to strengthen further m^{Ab}_{max}, it is necessary that $\gamma > m^{Ab}_{max}$ and therefore $\gamma > -\beta$. Because the barrier relies partly on selection against migrants (and to some extend on selection against hybrids if **A** can afford it), each further mutation still has an initial cost to pay (the selection against migrants, i.e. $\gamma > -\beta$).

If **C** can indeed strengthens the barrier ($\gamma > -\beta$), it will be the most efficient (stronger barrier) if $\epsilon_{BC} = -\frac{\gamma+\beta}{2}$. Extremely weak epistasis is not effective to prevent **B** from fixing and strong epistasis generates too much strain on allele **C**, and therefore this allele disappears really quickly. In this kind of scenario, if the first mutation generates a lot of epistasis (i.e. $\epsilon_{AB} \ll -\frac{\alpha+\beta}{2}$), and the second one is close to the optimum mentioned above, then **A** will probably be lost (blue and orange lines are identical around $\epsilon_{BC} = -\frac{\gamma+\beta}{2}$ in Fig. C5). If **C** generates strong epistasis, then the cost can be split and having two repressing island adaptations, sharing the cost of hybrids, generates a stronger barrier.

Invasion of **C** in that case is relatively easy and the presence of allele **A** always makes it easier as it decreases the frequency of allele **B** on the island.

As a conclusion, one can says that transition from 2 → 3 loci are basically identical to 1 → 2 loci, with minor differences: strengthening is slightly easier but less effective.

The case of an advantageous **B** allele on the island is detailed in the main manuscript. For clarity, we discuss only the interaction between **B** and **C**. Below, we present the alternative case: allele **C** interacting with allele **a**, illustrated in figure C6. We focus on a case unique to a three-locus barrier: an island allele **A** can participate to the genetic barrier despite having a

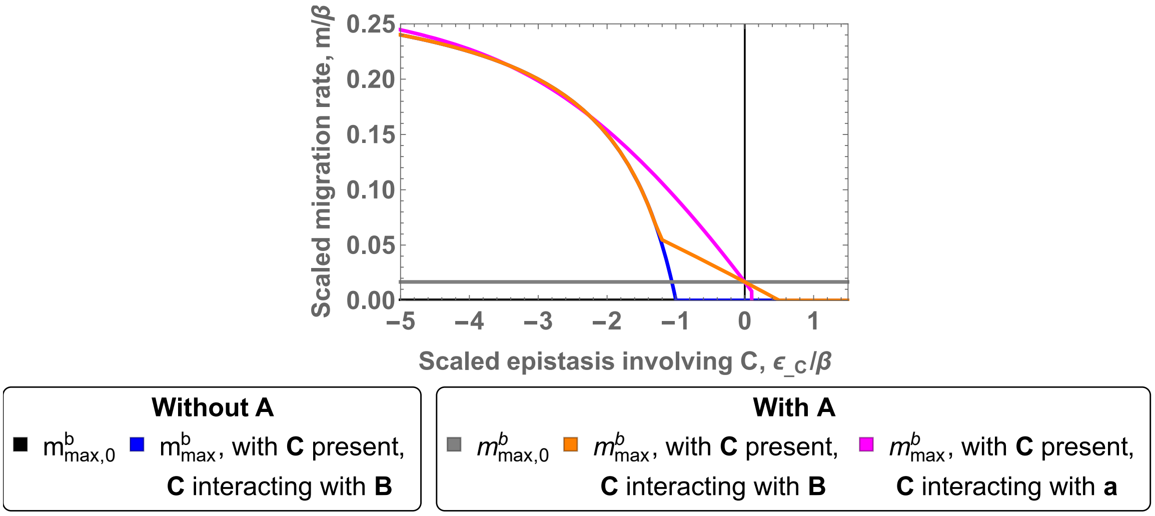

Figure C6: **An island adaptation can contribute to a genetic barrier stronger than its own selective advantage** ($m^b_{max} > \alpha$))

The x-axis corresponds to the epistasis between **C** and its interacting allele. The y-axis corresponds to the migration rate. The different lines correspond to m^b_{max}, the resistance to swamping at locus **B** under different scenarios. The initial single-locus and two-locus barriers, $m^b_{max,0} \leq m^{Ab}_{max,0}$, are given in black and gray. The impact of a new allele **C**, interacting with **B**, on the single-locus and two-locus genetic barrier is represented by the blue and orange lines, respectively. Lastly, the impact of an allele **C**, interacting with allele **a** is represented in magenta. Allele **B** is here advantageous on the island. The thin vertical black line indicates the absence of epistasis. This figure is obtained for ($\frac{\alpha}{\beta} = 0.2$, $\frac{\gamma}{\beta} = 1.2$ and $\frac{\epsilon_{AB}}{\beta} = -1.5$).

selective advantage smaller than the migration rate, $\alpha < m^b_{max}$ (magenta line above 0.2). If allele **C** interacts with allele **B** (orange line), then the strongest barrier is established while allele **A** is lost (orange line is on top of the the blue line). If allele **C** interacts with allele **a** (magenta line), the results are quite different. Allele **A** becomes critical to the existence of the barrier as it is the only allele that interacts with **B** and prevents its fixation. In this situation, allele **C** increases the marginal fitness of **A** by repressing allele **a**.

C 1.3 Maximal migration rate for maintenance of the DMI for all linkage architectures

Through Fig. 6 (top panels) and C7, one can deduce a certain number of conditions when the genetic barrier will be most of the time strengthened by the new mutation. As mentioned in Bank et al. [2012], there are two forces that shape the genetic barrier, selection against migrants and selection against hybrids. Therefore, any strengthening of the genetic barrier will be through one of these two mechanisms. We will first introduce the strengthening of the genetic barrier

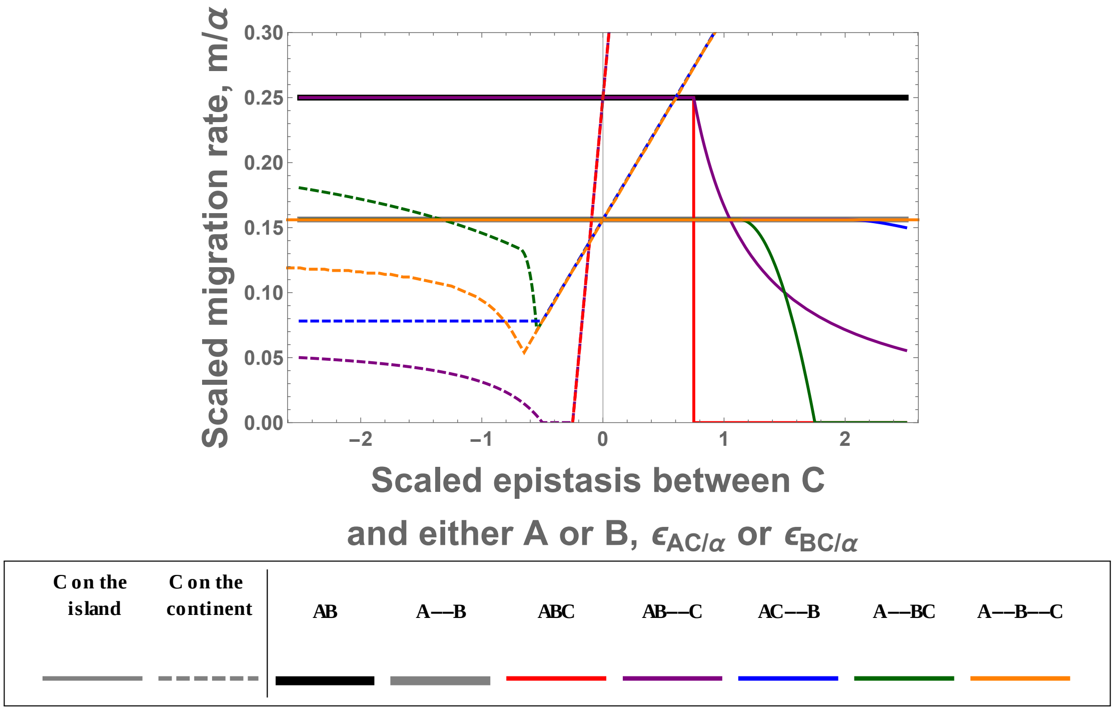

Figure C7: **Maximal migration rate for maintenance of the DMI between A and B, m^{Ab}_{max}, for the different linkage architectures in haploids**
The x-axis corresponds to the epistatic interaction between allele **C** and its interacting allele (either **A** if **C** appears on the continent ($\frac{\epsilon_{AC}}{\alpha}$) or **B** if **C** appears on the island($\frac{\epsilon_{BC}}{\alpha}$)). Both positive ($\epsilon_{BC} > 0, \epsilon_{AC} > 0$) and negative epistasis ($\epsilon_{BC} < 0, \epsilon_{AC} < 0$) are considered. The y-axis represents the maximal migration rate for maintenance, $\frac{m^{Ab}_{max}}{\alpha}$. If a curve is not visible, it means that $\frac{m^{Ab}_{max}}{\alpha} = 0$. Each color corresponds to a different linkage architecture; plain lines indicate that **C** appears on the island, dashed lines on the continent. The black and gray lines serve as a reference for a two-locus genetic barrier between **A** and **B**, for tight linkage and loose linkage. Other parameters used are: $\frac{\beta}{\alpha} = .75$, $\frac{\gamma}{\alpha} = -0.5$ and $\frac{\epsilon_{AB}}{\alpha} = -2$

based on an increase of selection against migrants. A more detailed analysis for each linkage architecture is available in the next section C 2.

- **C** appears at a locus in tight linkage with either **A** and/or **B** on the island. In that case, the genetic barrier is strengthened if **C** is advantageous on the island (by reinforcing the selective advantage of the "island alleles" either **AC** or **bC**). In addition, it requires that the epistasis between **B** and **C** is mainly negative, otherwise the new allele will boost the selective advantage of the "continental allele" **B**, making it easier for it to fix on the island (see solid green, red and blue lines in Fig. 6(a), **??**). In particular, the linkage architecture **AC-B** (in solid blue lines) behaves similarly to a substitution of allele **A** by allele **A'**, with **A'=AC**, presented in the previous section.

- **C** appears at a locus in tight linkage with either **A** and/or **B** on the continent. This requires that **C** is deleterious on the island. In this case, the genetic barrier is strengthened by

making the "continental alleles" (**aC** or **BC**) even less fit on the island than they were before. (see green, red and blue solid lines in Fig. 6(a), **??** and Fig. C7).

- **C** appears on the continent and generates positive epistasis with allele **A**. In this case, allele **C** can easily fix on the island. By doing so, it will reinforce the selective advantage of allele **A** on the island ($\alpha \to \alpha + \epsilon_{AC}$) and therefore strengthen the genetic barrier (see in Fig. 6(a), **??** and C7 the dashed lines for $\epsilon_{AC} > 0$). This happens regardless of the values of γ, since fixation of **C** removes the dependency on γ. However, if **C** is highly deleterious this behavior will only be observed for strong positive epistasis only for certain linkage architectures (Fig. C7, red and purple dashed lines). Fixation of **C** makes its position in the genome irrelevant and therefore several scenarios simplify to the same case, ABC and AB–C (red and purple dashed lines) and AC–B, A–BC and A–B–C (blue, green and orange dashed lines).

Now we will focus on the strengthening of the genetic barrier based on an increase of selection against hybrids.

- All loci are in loose linkage. **C** appears on the island. In that case, the new mutation helps to strengthen the genetic barrier by splitting the cost of selection against hybrids across two loci. This effect can be quite weak when selection against migrants is the main force for the original DMI (Fig. 6(a), orange solid line).

- **C** appears at a locus in tight linkage with B on the continent. The genetic barrier can be strengthened if the effect of **C** is weak but the incompatibility between **A** and **C** is quite strong. This effect is only visible if the genetic barrier between A and B relies on selection against hybrids (Fig. **??**, dashed green line).

We observe in Fig. C7 a minimum for the following linkage architecture: all loci are in loose linkage and **C** appears on the continent (orange dashed line). This minimum can be explained as follow: if negative epistasis is weak, fixation of **C** allows A to stay polymorphic. Therefore, the weaker the epistasis the better for allele **A** as it pays a weaker hybrid cost (m^{Ab}_{max} is increasing). For larger negative epistasis, it is no longer the case. Only the internal equilibrium is stable. Therefore, the more negative epistasis, the longer it will be stable because **C** is purged faster (if **C** increases in frequency then the frequency of allele **A** decreases, making swamping easier). This corresponds to the decreasing part of m^{Ab}_{max}. This can be summarized as follows: if **A** can prevent introgression of **C**, the stronger the incompatibility, the better. If not, then the best option for allele **A** is to not interact with **C** at all or even better generate positive epistasis.

C 1.4 Is invasion of allele C easier?

Here we focus on the influence of the first DMI on the fate of a new mutation **C** on the island. In the absence of any island polymorphism, a new substitution **C** can invade only if $\gamma > m$, Fig. C8 black line. If B is polymorphic on the island ($-\beta > m$), then the new substitution has to fulfill the conditions detailed in equation (C5).

$$\begin{cases} \gamma > m\left(1 + \frac{\epsilon_{BC}}{\beta}\right) & \text{if C is in loose linkage with B} \\ \gamma > 0 & \text{if C in tight linkage with B, } \mathbf{C} \text{ associated to allele } \mathbf{b} \\ \gamma > m - \epsilon_{BC} & \text{if C in tight linkage with B, } \mathbf{C} \text{ associated to allele } \mathbf{B} \end{cases} \tag{C5}$$

Now, if we assume that both A and B are polymorphic on the island, then the substitution has to fulfill the conditions detailed in equation (C6) to possibly invade.

$$\begin{cases} \gamma > m + \frac{\epsilon_{BC}\alpha}{2\epsilon_{AB}}\left(1 - \sqrt{1 - \frac{4m\epsilon_{AB}}{\alpha(\epsilon_{AB}+\beta)}}\right) & \text{if A, B and C are all in loose linkage} \\ \gamma > m\left(1 + \frac{1}{-\beta+\alpha}\right) & \text{if C is in loose linkage with A and B} \\ & \text{and A and B are in tight linkage} \\ \gamma > 0 & \text{if A, B and C are in tight linkage, } \mathbf{C} \text{ associated to } \mathbf{Ab} \\ \gamma > \alpha - \beta - \epsilon_{BC} & \text{if A, B and C are in tight linkage, } \mathbf{C} \text{ associated to } \mathbf{aB} \\ \gamma > -\frac{\alpha\epsilon_{BC}\left(-1+\sqrt{1-\frac{4m\epsilon_{AB}}{\alpha(\epsilon_{AB}+\beta)}}\right)}{2\epsilon_{AB}} & \text{if A, B are in loose linkage, C in tight linkage} \\ & \text{with A, } \mathbf{C} \text{ associated to } \mathbf{A} \\ \gamma > \frac{\alpha\left(\epsilon_{AB}\left(1+\sqrt{1-\frac{4m\epsilon_{AB}}{\alpha(\epsilon_{AB}+\beta)}}\right)+\epsilon_{BC}\left(1-\sqrt{1-\frac{4m\epsilon_{AB}}{\alpha(\epsilon_{AB}+\beta)}}\right)\right)}{2\epsilon_{AB}} & \text{if A, B are in loose linkage, C in tight linkage} \\ & \text{with A, } \mathbf{C} \text{ associated to } \mathbf{a} \end{cases} \tag{C6}$$

If all loci are in tight linkage, as soon as there is one locus polymorphic on the island, then the first locus "pays" the price of migration and the new substitution needs only to be advantageous. Of course, it requires that the new mutation **C** appears in the right background. For selection against migrants, the cost of migration needs to be paid only once. If C is in loose linkage, then it is most of the time easier to invade with A and B polymorphic than just with B polymorphic, Fig. C8 gray and orange lines. A and C here do not interact directly, but indirectly, through the frequency at B, which is reduced by the polymorphism at locus A. For selection against hybrids, the cost of hybrids is therefore shared between the different "island"

loci but the migration cost has to be paid by each locus independently. This is not true when m is close to $\alpha(\epsilon_{AB} - \beta)/4/\epsilon_{AB}$ and $-\epsilon_{AB} > \beta$. In this case, **B** is deleterious on the island on its own and epistasis creates a hybrid cost that does not help (because selection against migrants is the main force) and reduces the fitness of all genotypes on the island, making it harder for a new mutation to invade.

When A and B are in loose linkage, and C appears in tight linkage with one of the two loci, the results are no longer so clear, since we need to take into account the intra-locus competition between the different alleles. For example, if C appears in tight linkage with A, with **C** associated to allele **A** (resp. **a**), then we have three possible alleles at locus A: **ac**, **Ac** and **AC** (resp. **ac**, **Ac**, **aC**). Invasion of the new allele then also depends of allele **Ac**, Fig. C8 blue (for allele **AC**) and cyan (for allele **aC**) lines. Competition mainly happens if **C** appears with allele **a** since it has to overcome the selective advantage of **A** on the island before it even has time to recombine into the “optimal” genome.

In tight linkage, given that we assume $r \to 0$, the fate of the new mutation will still be determined by which allele it appears with. To form the next step of the genetic barrier, one needs to wait for either a really good **C** mutation (that can invade in both backgrounds, and then an unlikely recombination event will form the best haplotype) or for **C** to appear directly in the “good” background. If **C** appears at a locus in tight linkage with B, associated with allele **b**, then the polymorphism at locus A does not matter and the case, where only B is polymorphic, has already been explained above.

To complete what has been presented in the previous paragraph, one needs to keep in mind that it can also be difficult to hit the proper background when C is in tight linkage with another locus. This is particularly true when $m \to m_{max}^{Ab}$, then either $p_A \to 0$ and/or $p_B \to 1$. This happens if A and B are in tight linkage and if A and B are in loose linkage (if $\beta \leq \epsilon_{AB} \leq \text{Min}\left[\frac{\alpha}{2}, \alpha + \beta\right]$ $p_A \to 0$ or if $\alpha + \beta \leq \epsilon_{AB} \leq -\beta$, $p_B \to 1$). As a consequence, the new substitution has a low probability to be associated with the “island-adapted” allele (**A** or **b**) or to participate in the unlikely recombination event (because recombination depends on the frequency of the different alleles).

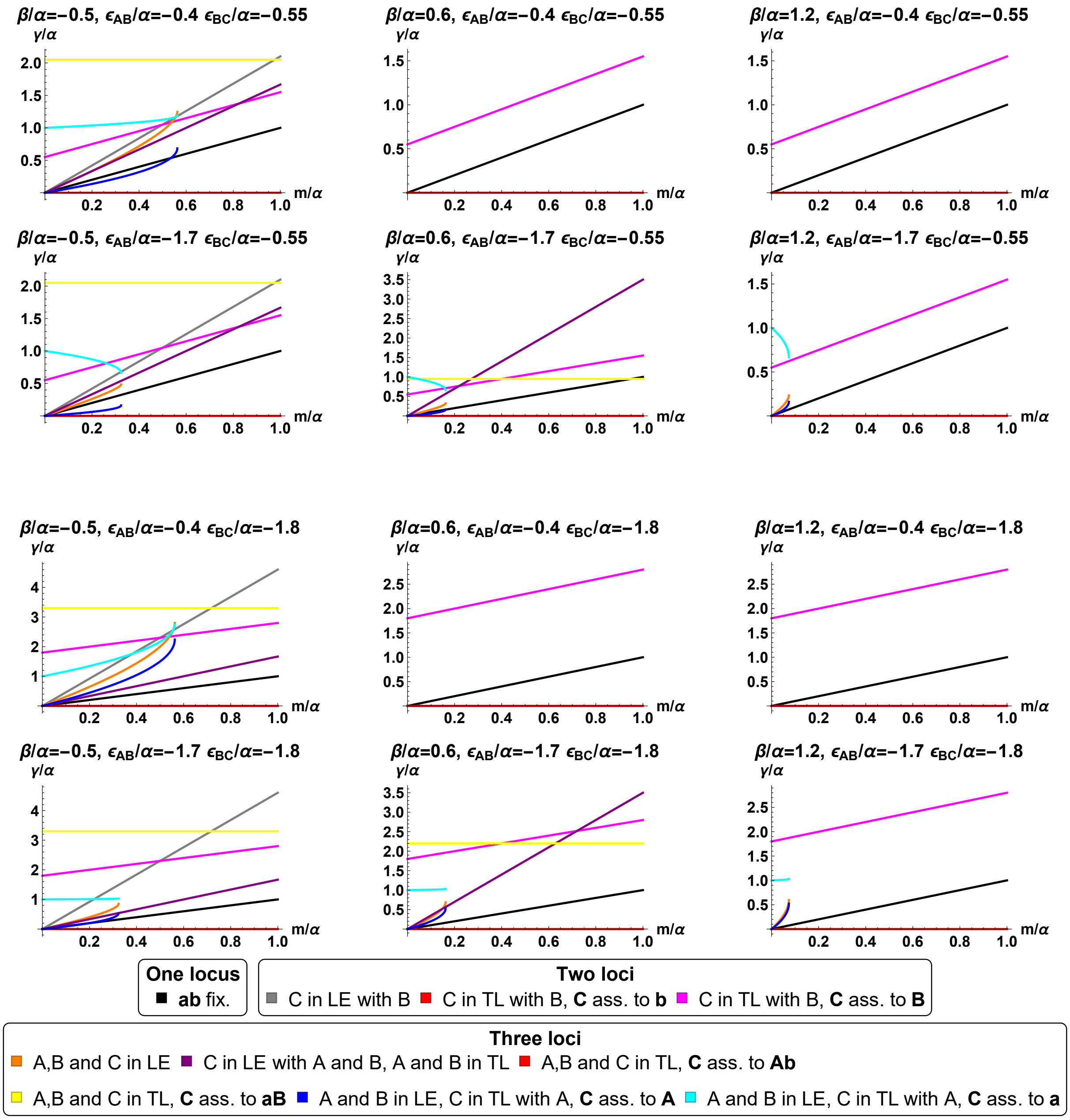

Figure C8: **Minimum selective advantage γ for a new mutation C to invade, if C is incompatible with B**

We represent the minimal selective advantage γ of a new mutation **C** on the island to invade as a function of the migration rate. We consider different linkage architectures, with TL standing for tight linkage and LE for loose linkage. Each color corresponds to a different case presented in (C5) or (C6). The case C in tight linkage with B and in loose linkage with A is in loose linkage is equivalent to the two-locus case. Values for the different parameters are given above each panel.

C 2 Extension of the genetic barrier: formula and details for each linkage architecture

In this section, we detail when each architecture strengthens the genetic barrier, $m^{Ab}_{max,0}$. In the main text, we always asked whether **C** can strengthen the old barrier, without imposing

any constraint on **C**. When giving the expression of m_{max}^{Ab}, for all the different linkage architectures, we only provide the expression of the barrier but not when it holds, as the conditions are extremely long. Instead, we describe what happens if the barrier is given by this specific expression. To describe the different equilibria, we will reuse the notation above, for example S_{AB} stands for an equilibrium with loci A and B polymorphic, C is monomorphic.

All the equations (except for all 3 loci tight linkage) come in 2 parts: m_{max}^{AbC} or m_{max}^{Abc} when the genetic barrier is obtained with C polymorphic and m_{max}^{Ab} if **C** is either lost or fixed. If one is interested in the genetic barrier formed by having all loci polymorphic, then only m_{max}^{AbC} or m_{max}^{Abc} matters. If, as we did in the main text, we are interested in how the initial barrier is affected, i.e. both A and B stay polymorphic regardless of the fate of allele **C**, then we need to consider both parts of the equation to obtain the right expression for the genetic barrier (each part of the equation has one element that is valid, and the barrier is given by the maximum between these 2 valid expressions).

C 2.1 The ABC linkage architecture strengthens the genetic barrier.

When all loci are in tight linkage, the equations for the genetic barrier, m_{max}^{Ab}, are given in equation (C7) if **C** appears on the island and (C8) if **C** appears on the continent. When comparing these new barriers to the two-locus barrier, we observe the following pattern: as long as the best haplotype is **AbX** then the genetic barrier is at least unchanged (with **X** standing for **c** or **C** depending of the circumstances). This happens under the following conditions:

- **C** appears on the island
  - If the two-locus barrier exists ($\alpha > \beta$ and $-\epsilon_{AB} > \beta$), the genetic barrier is strengthened if **C** is advantageous ($\gamma > 0$) on the island and the epistasis between **B** and **C** is either negative or weak if positive ($\epsilon_{BC} < \alpha - \beta$) so **aBC** cannot replace **AbC**. (Fig. C9, panels 1-8, 10-12)
  - If the two-locus barrier does not exist, the three-locus barrier can be created if **C** makes haplotype **AbC** the fittest haplotype on the island. In particular, it implies that **AbC** is fitter than the continental haplotype ($\gamma > -(\alpha - \beta)$). In addition, **C** must prevent the fixation of **B** ($\gamma > \beta + \epsilon_{AB}$) through haplotype **ABc**. The epistasis between **B** and **C** needs to be negative enough ($\epsilon_{BC} < \alpha - \beta$ and $\epsilon_{BC} < -\beta - \epsilon_{AB}$) so **aBC** or **ABC** cannot replace **AbC** on the island (Fig. C9, panels 9, 13-16).

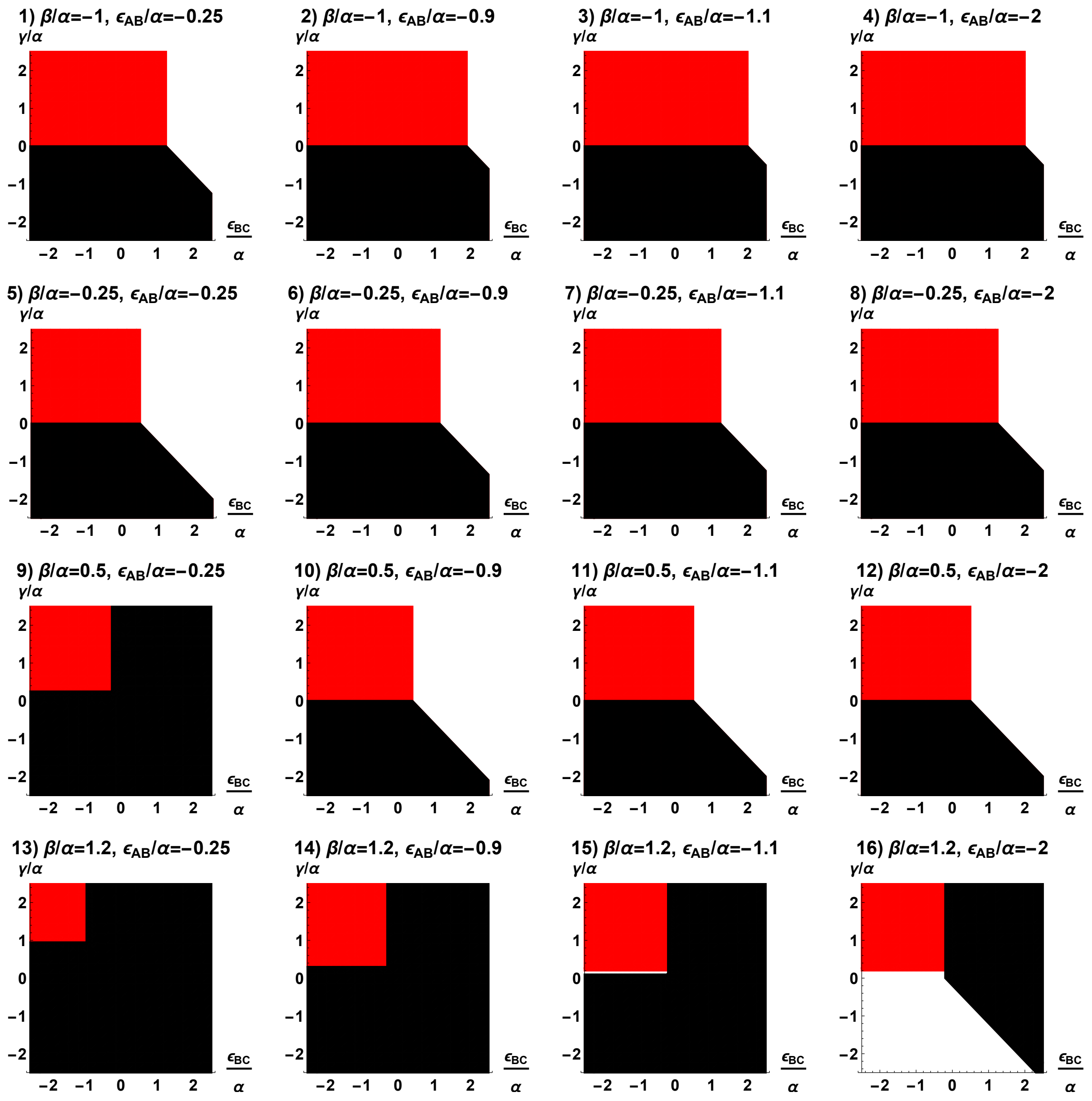

Figure C9: **Comparison between the genetic barrier for ABC and the old one AB, C appearing on the island**

The x-axis corresponds to the incompatibility between **B** and **C**. The y-axis corresponds to selective advantage of **C** on the island. Red indicates that the genetic barrier is strengthened, black that the genetic barrier is unchanged and white that it is weakened or worse. If ($\alpha < \beta$ or $-\epsilon_{AB} < \beta$), the black area indicates that the two-locus barrier is absent and the third locus does not change this fact.

- **C** appears on the continent
  - If the two-locus barrier exists ($\alpha > \beta$ and $-\epsilon_{AB} > \beta$), the genetic barrier is strengthened if **C** is deleterious on the island ($\gamma < 0$) or the epistasis between **A** and **C** is positive ($\epsilon_{AC} < -\gamma$) (Fig. C10, panels 1-8, 10-12)
  - If the two-locus barrier does not exist in the first place, fixation of **C** ($-\gamma < \epsilon_{AC}$) can allow the formation of the two-locus barrier between A and B, if the epistasis between

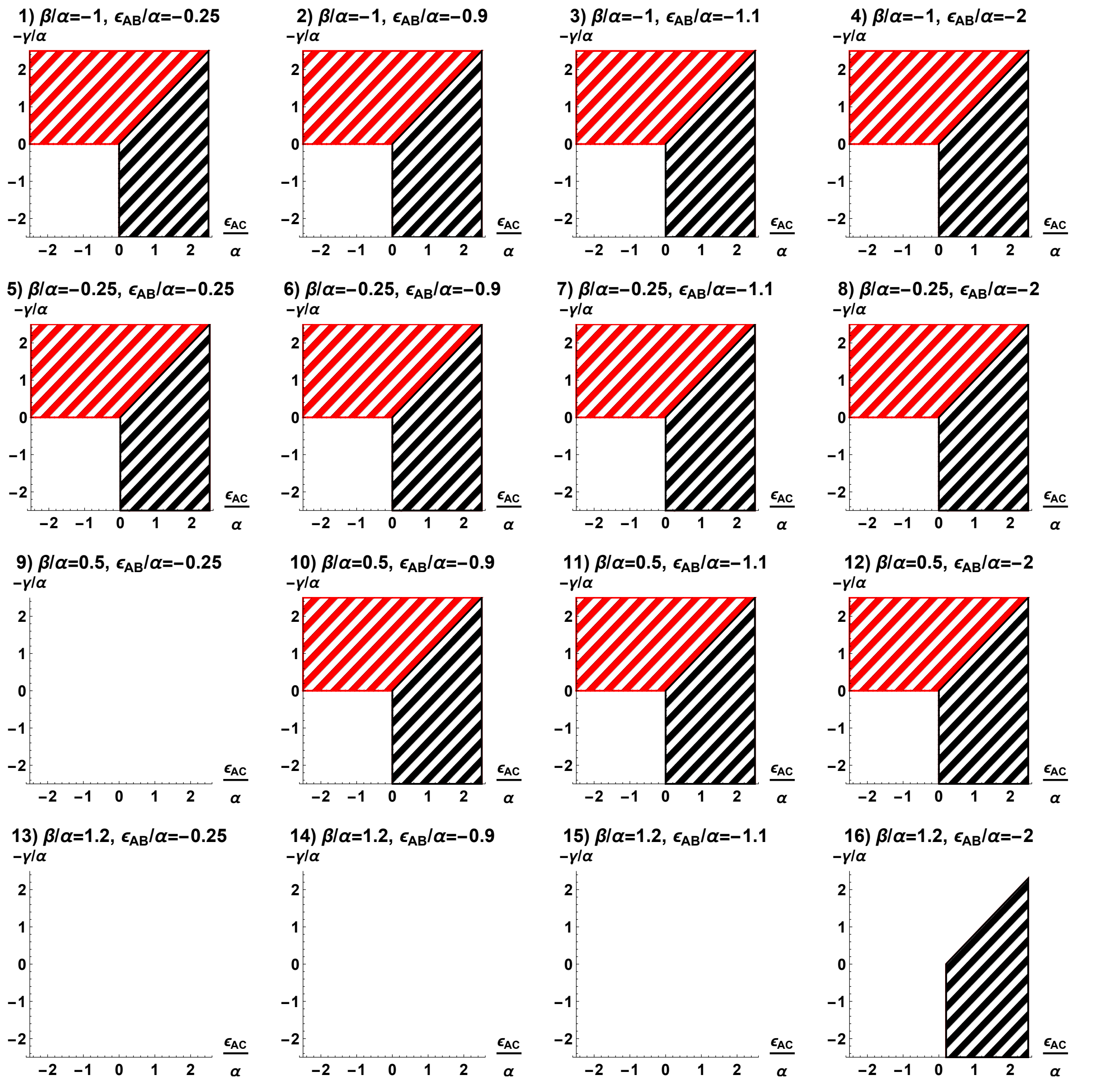

Figure C10: **Comparison between the genetic barrier for ABC and the old one AB, C appearing on the continent**

The x-axis corresponds to the incompatibility between **A** and **C**. The y-axis corresponds to selective advantage of **C** on the island. Stripped red indicates that the genetic barrier is strengthened, stripped black that the genetic barrier is strengthened through fixation of **C** on the island and white that it is weakened or worse.

A and **C** is strong enough ($\epsilon_{AC} > \beta - \alpha$) and negative epistasis between A and B is strong enough ($-\epsilon_{AB} > \beta$). This corresponds to increasing the selective advantage of **A** on the island ($\alpha' = \alpha + \epsilon_{AC}$). (Fig. C10, panels 9, 13-16)

$$\begin{cases} m^{Ab}_{max} = \alpha - \beta + \gamma & \text{if } \alpha > 0 \text{ and } \gamma > 0 \text{ and } \gamma - \epsilon_{AB} > \beta \text{ and } -\epsilon_{AB} - \epsilon_{BC} > \beta \text{ and } \alpha - \epsilon_{BC} > \beta \\ m^{Ab}_{max} = \alpha - \beta & \text{if } \gamma < 0 \text{ and } \alpha > 0 \text{ and } \beta < -\epsilon_{AB} \text{ and } \alpha - \epsilon_{BC} > \beta + \gamma \text{ and } \beta + \gamma < -\epsilon_{AB} - \epsilon_{BC} \end{cases} \tag{C7}$$

$$\begin{cases} m^{Ab}_{max} = \alpha - \beta - \gamma & \text{if } \alpha > \beta \text{ and } \beta + \epsilon_{AB} < 0 \text{ and } \beta + \gamma + \epsilon_{AB} + \epsilon_{AC} < 0 \text{ and } ((\epsilon_{AC} < 0 \text{ and } \gamma \leq 0) \\ & \text{or } (\gamma + \epsilon_{AC} < 0 \text{ and } (\epsilon_{AC} \geq 0 \text{ or } \alpha > \gamma))) \\ m^{Ab}_{max} = \alpha - \beta + \epsilon_{AC} & \text{if } \beta + \epsilon_{AB} < 0 \text{ and } (\epsilon_{AC} \geq 0 \text{ or } \beta + \gamma + \epsilon_{AB} + \epsilon_{AC} \geq 0 \text{ or } \alpha \leq \beta \text{ and } \gamma > 0) \\ & \text{and } \alpha + \epsilon_{AC} > 0 \text{ and } \gamma + \epsilon_{AC} > 0 \text{ and } \alpha + \gamma + \epsilon_{AC} > 0 \text{ and } \alpha + \gamma + \epsilon_{AC} > \beta \end{cases} \tag{C8}$$

C 2.2 The AB-C linkage architecture strengthens the genetic barrier.

When A and B are in tight linkage and C in loose linkage , the expression for the strength of the genetic barrier is given by equation (C9) if **C** appears on the island and by (C10) if **C** appears on the continent. When comparing the new barrier to the two-locus barrier, we observed a strengthening of the genetic barrier under the following conditions:

- **C** appears on the island
  - First, we assume that the two-locus barrier already exists. Only a mutation of sufficient direct effect strengthens the genetic barrier ($\gamma > \alpha - \beta$ otherwise it will always be the first to disappear). If its effect γ is only slightly larger than the strength of the genetic barrier, then only weak epistasis may lead to a strengthening of the barrier, otherwise the hybrid cost is too high and allele **C** is lost (Fig. C17, panels 1-8). If the effect of **C**, γ, is much larger than the strength of the genetic barrier, then strong negative epistasis can help strengthen the genetic barrier by further repressing the migrant haplotype (**aBc**) through selection against hybrids. (Fig. C17, panels 10-12)
  - If the two-locus barrier does not exist, the presence of a third locus allows the formation of the genetic barrier as long as **C** is advantageous on the island and the negative epistasis between **B** and **C** is strong enough to prevent the fixation of allele **B** (Fig. C17, panels 9, 13-16).
- **C** appears on the continent

– If the two-locus barrier exists, the genetic barrier is strengthened as long as there is positive epistasis between **A** and **C**, that increases the marginal fitness of **A** on the island (Fig. C18, panels 1-8, 10-12).

– If the two-locus barrier does not exist, then the genetic barrier is created under the same condition then the ABC architecture, i.e. through fixation of C ($-\gamma < \epsilon_{AC}$) and if the epistasis between **A** and **C** is strong enough ($\epsilon_{AC} > \beta - \alpha$) and the negative epistasis between **A** and **B** is strong enough ($-\epsilon_{AB} > \beta$), (Fig. C18, 9, 13-16).

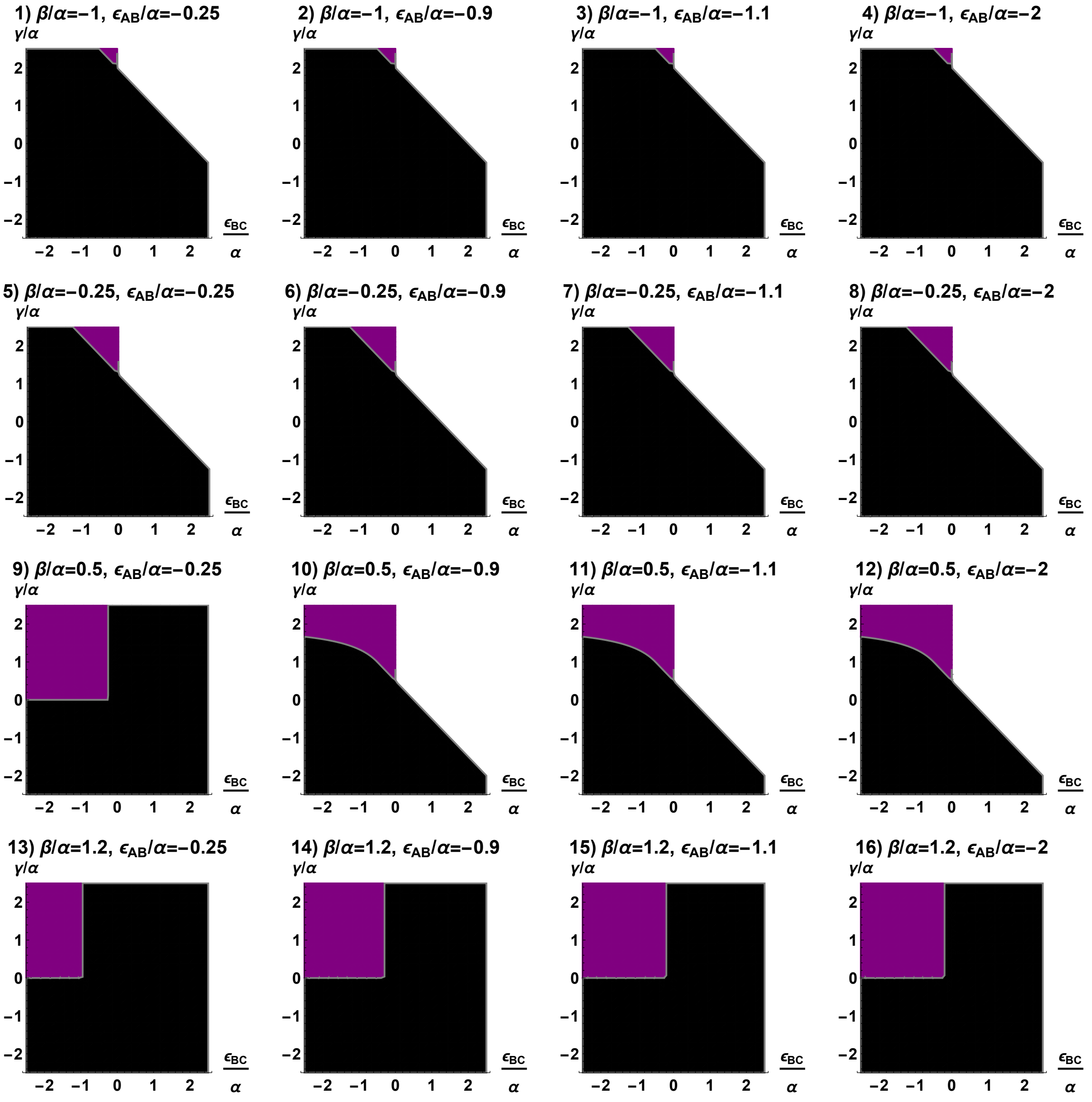

Figure C11: **Comparison between the genetic barrier for AB-C and the old one AB, C appearing on the island**

The x-axis corresponds to the incompatibility between **B** and **C**. The y-axis corresponds to selective advantage of **C** on the island. Purple indicates that the genetic barrier is strengthened, black that the genetic barrier is unchanged and white that it is weakened or destroyed.

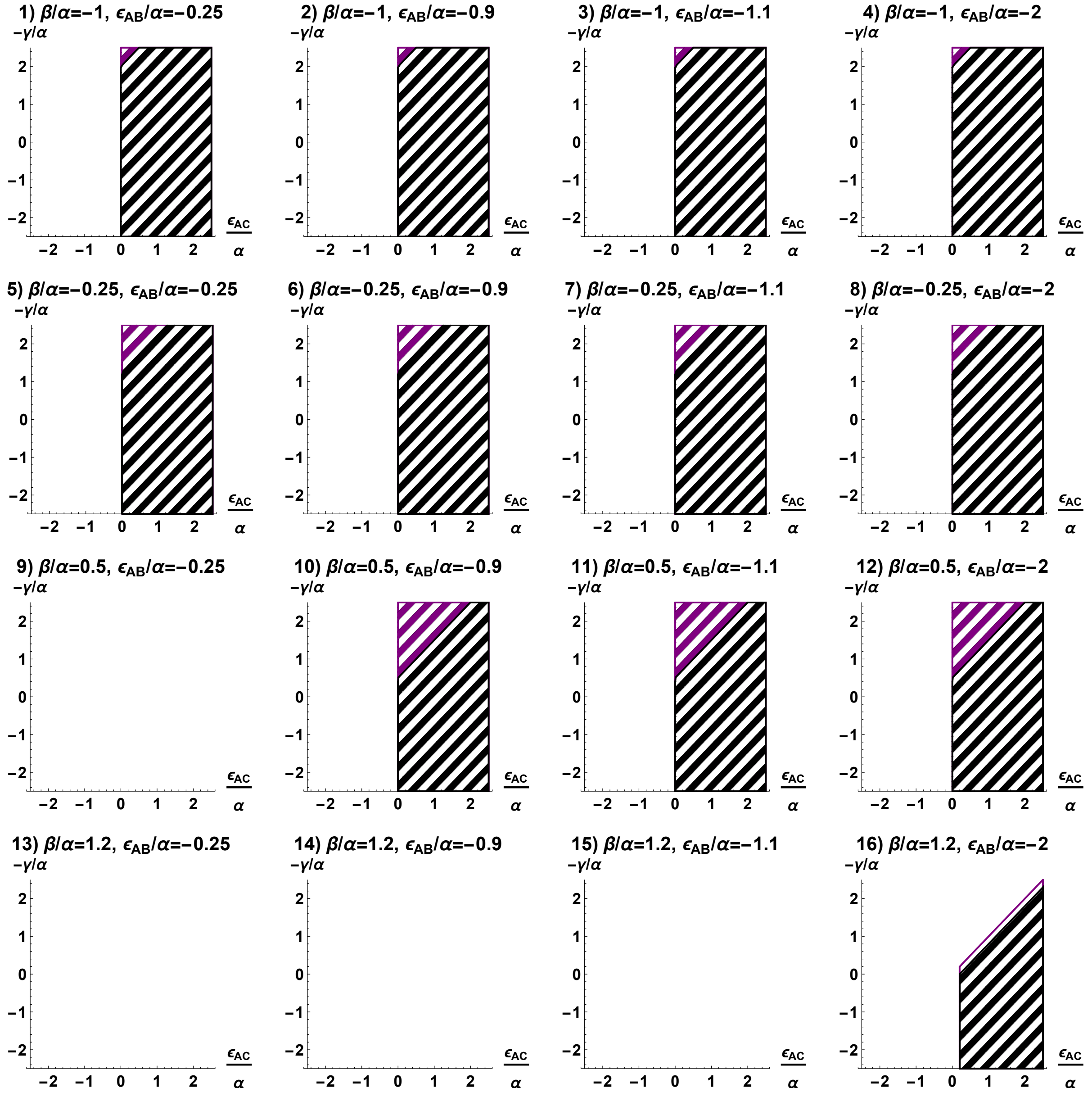

Figure C12: **Comparison between the genetic barrier for AB-C and the old one AB, C appearing on the continent**

The x-axis corresponds to the incompatibility between **A** and **C**. The y-axis corresponds to selective advantage of **C** on the island. Stripped purple indicates that the genetic barrier is strengthened, stripped black that the genetic barrier is strengthened through fixation of **C** on the island and white that it is weakened or destroyed.

$$
\begin{cases}
 & \text{C stays polymorphic} \\
m_{max}^{AbC} = \frac{\gamma(-\alpha+\beta+\epsilon_{BC})}{4\epsilon_{BC}} & \text{equilibrium collides with an internal unstable one} \\
m_{max}^{AbC} = \frac{(\gamma+\epsilon_{BC})(\alpha-\beta-\epsilon_{BC})}{\gamma} & \textbf{A} \text{ is lost, } \textbf{B} \text{ fixes} \\
m_{max}^{AbC} = \frac{\gamma(\alpha-\beta)}{\alpha-\beta-\epsilon_{BC}} & \textbf{C} \text{ is lost and } S_{AB} \text{ is unstable} \\
m_{max}^{AbC} = \frac{\gamma(\alpha+\epsilon_{AB})(\beta+\epsilon_{AB}+\epsilon_{BC})}{\epsilon_{BC}(\alpha-\beta-\epsilon_{BC})} & \textbf{Ab} \text{ invades the equilibrium} \\
 & \textbf{C} \text{ is lost} \\
m_{max}^{Ab} = \frac{\gamma(\alpha-\beta)}{\alpha-\beta-\epsilon_{BC}} & \textbf{C} \text{ invades and } S_{ABC} \text{ is unstable} \\
m_{max}^{Ab} = \alpha-\beta & \textbf{Ab} \text{ is replaced by another allele (1-locus dynamics)}
\end{cases}
\tag{C9}
$$

$$
\begin{cases}
 & \textbf{C} \text{ stays polymorphic} \\
m_{max}^{Abc} = \frac{(\alpha-\beta)(\gamma+\epsilon_{AC})}{4\epsilon_{AC}} & \text{equilibrium collides with an internal unstable one} \\
m_{max}^{Abc} = -\frac{(\gamma+\epsilon_{AC})(\alpha-\beta+\epsilon_{AC})}{\alpha-\beta} & \textbf{C} \text{ fixes and } S_{AB} \text{ is unstable} \\
m_{max}^{Abc} = \frac{\gamma(\alpha-\beta)}{\gamma+\epsilon_{AC}} & \textbf{A} \text{ is lost and } \textbf{B} \text{ fixes, ie } \textbf{Ab} \text{ is replaced by } \textbf{aB} \\
m_{max}^{Abc} = -\frac{\alpha\beta(\gamma+\epsilon_{AC})}{\epsilon_{AC}(\alpha-\beta)} & \textbf{ab} \text{ invades the equilibrium)} \\
 & \textbf{C} \text{ fixes} \\
m_{max}^{Ab} = -\frac{(\gamma+\epsilon_{AC})(\alpha-\beta+\epsilon_{AC})}{\alpha-\beta} & \textbf{c} \text{ invades and } S_{ABC} \text{ is unstable} \\
m_{max}^{Ab} = \alpha-\beta+\epsilon_{AC} & \textbf{Ab} \text{ is replaced by another allele (1-locus dynamics)}
\end{cases}
\tag{C10}
$$

C 2.3 The A-BC linkage architecture strengthens the genetic barrier.

When A and B are in loose linkage and C in tight linkage with B, the expression for the strength of the genetic barrier is given in equation (C11) if **C** appears on the island and (C12) if **C** appears on the continent. When comparing this new barrier to the two-locus barrier, we observed a strengthening of the genetic barrier under the following conditions:

- **C** appears on the island
  - If the two-locus barrier already exists ($-\epsilon_{AB} > \beta$), only advantageous mutations on the island can strengthen the genetic barrier, through stronger selection against migrants. This holds as long as the (positive) epistasis between **B** and **C** is not too strong, to avoid the fixation of “allele” **BC** (Fig. C13, panels 1-8, 10-12,16).
  - If the two-locus barrier does not exist, then the presence of a polymorphism at locus C allows the formation of a genetic barrier if **C** is advantageous enough ($\gamma > \beta + \epsilon_{AB}$) and epistasis between **B** and **C** is negative enough ($-\epsilon_{AC} < \beta + \epsilon_{AB}$) to prevent fixation of allele **B** on the island (Fig. C13, panels 9, 13-15). The genetic barrier is strengthened through stronger selection against hybrids. While selection against migrants does not play a direct role on the new barrier, it is absolutely essential for allele **C** to remain on the island and therefore to generate the required selection against hybrids.
- **C** appears on the continent

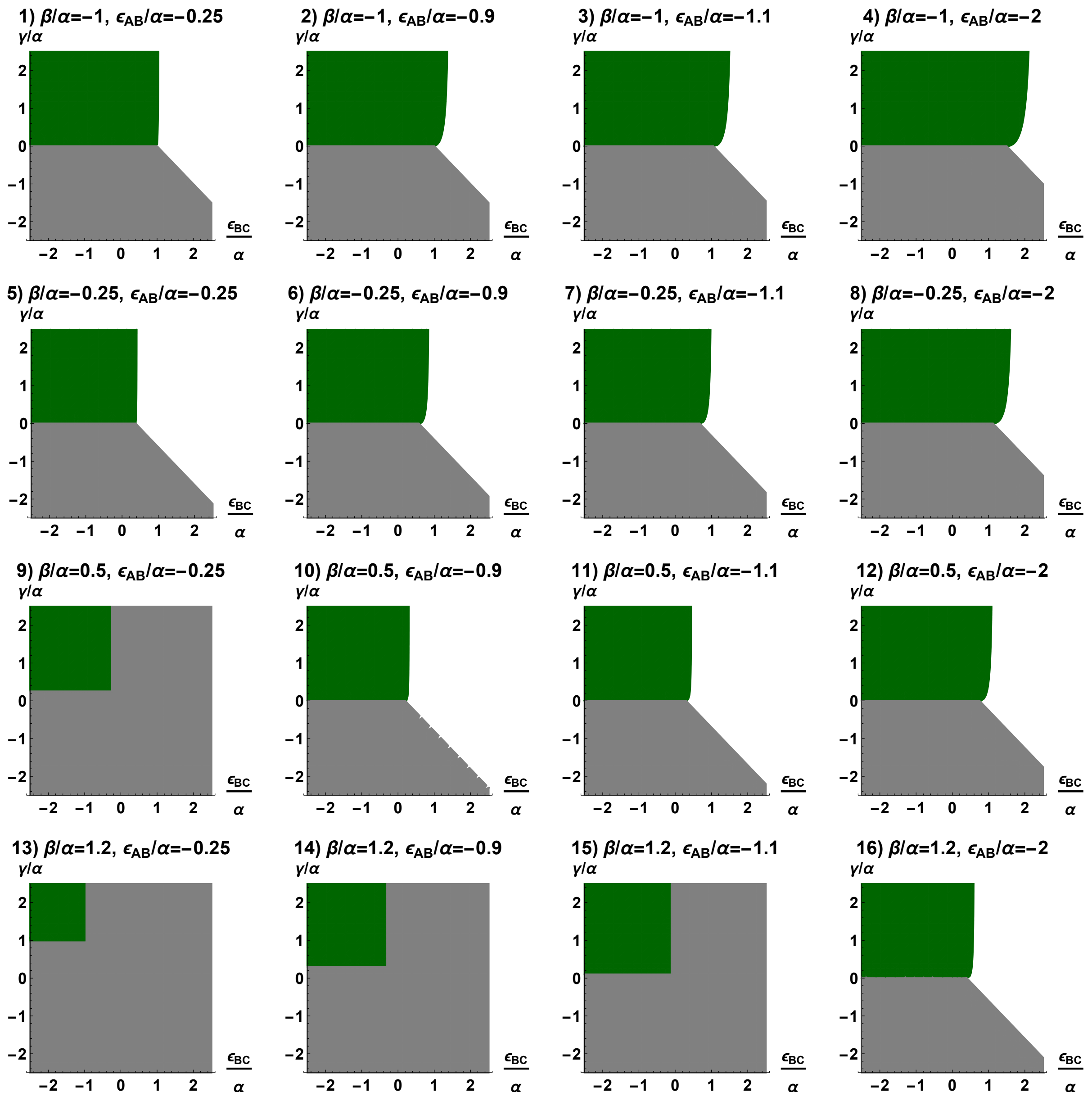

Figure C13: **Comparison between the genetic barrier for A-BC and the old one A-B, C appearing on the island**

The x-axis corresponds to the incompatibility between B and C. The y-axis corresponds to selective advantage of **C** on the island. Green indicates that the genetic barrier is strengthened, gray that the genetic barrier is unchanged and white that it is weakened or destroyed.

– If the two-locus barrier exists ($-\epsilon_{AB} > \beta$), a new mutation with positive epistasis with **A** can strengthen the genetic barrier regardless of its own selective advantage on the island. This corresponds again to an improvement of the marginal fitness of **A** on the island. In addition, mutation with negative epistasis can strengthen the genetic barrier as well if they are deleterious enough ($\gamma < 0$). In this last case, selection against migrants is strong enough to make the hybrids cost negligible. This corresponds to a strengthening of selection against the migrant haplotype.

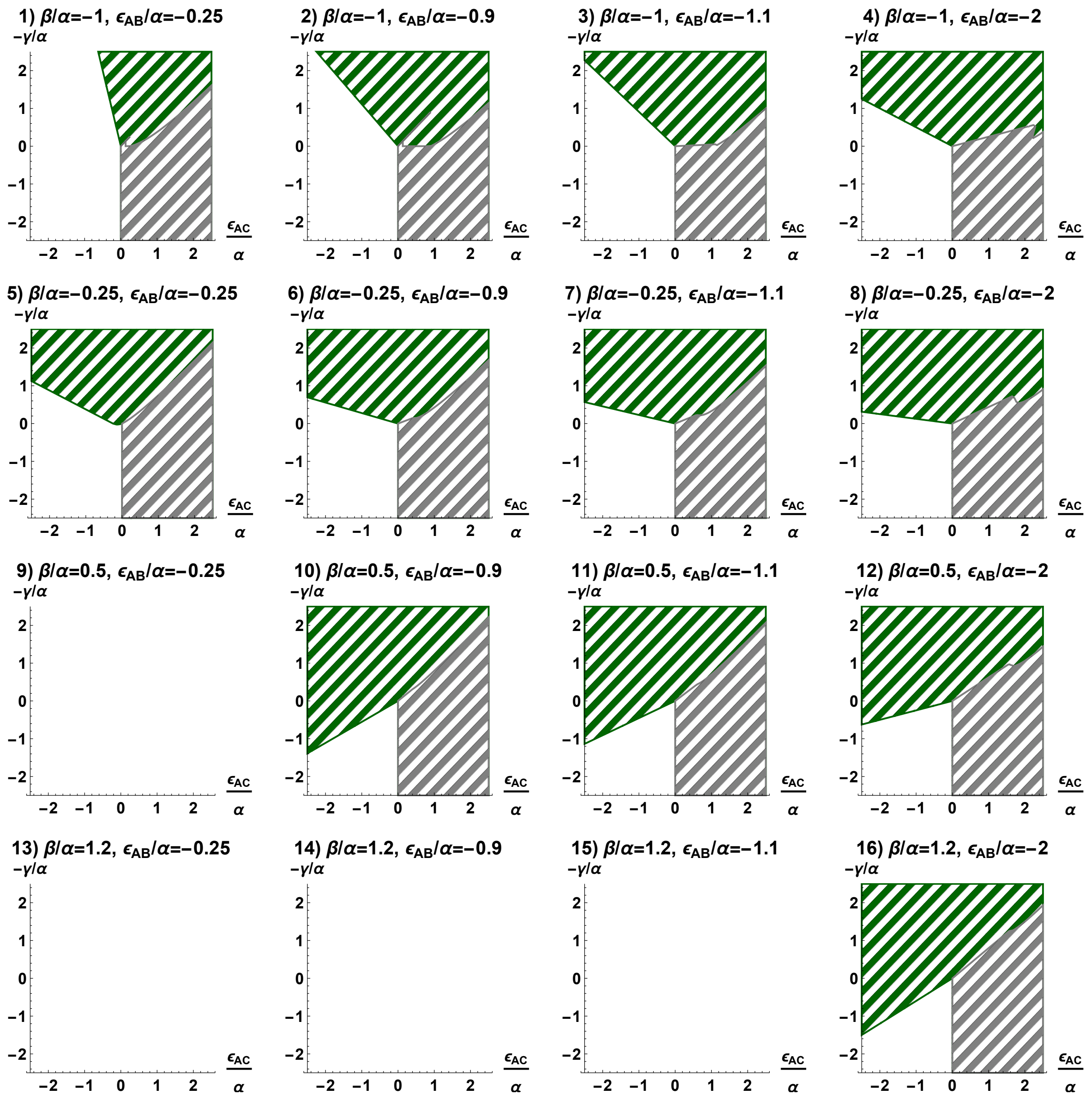

Figure C14: **Comparison between the genetic barrier for A-BC and the old one A-B, C appearing on the continent**

The x-axis corresponds to the incompatibility between **A** and **C**. The y-axis corresponds to selective advantage of **C** on the island. Stripped green indicates a strengthening of the genetic barrier, stripped gray that the genetic barrier is strengthened through the fixation of **C** on the island and white that it is weakened or worse.

– If the two-locus barrier does not exist, then it is impossible to form a barrier with a third locus.

$$
\begin{cases}
 & \textbf{C}\text{ stays polymorphic} \\
m^{AbC}_{max} = \frac{\alpha(\beta-\gamma+\epsilon_{AB})}{4\epsilon_{AB}} & \text{equilibrium collides with internal unstable} \\
m^{AbC}_{max} = -\frac{(\alpha+\epsilon_{AB})(\beta-\gamma+\epsilon_{AB})}{\alpha} & \textbf{C}\text{ is lost and }\textbf{B}\text{ fixes, ie }\textbf{bC}\text{ is lost} \\
m^{AbC}_{max} = \frac{\alpha(\beta-\gamma)}{\beta-\gamma+\epsilon_{AB}} & \textbf{A}\text{ is lost} \\
m^{AbC}_{max} = -\frac{\alpha(\gamma+\epsilon_{BC})(\beta+\epsilon_{AB}+\epsilon_{BC})}{\epsilon_{AB}(\beta-\gamma+\epsilon_{AB})} & \textbf{BC}\text{ invades} \\
 & \textbf{C}\text{ is lost} \\
m^{Ab}_{max} = \frac{\alpha(\beta+\epsilon_{AB})}{4\epsilon_{AB}} & \text{equilibrium collides with internal unstable} \\
m^{Ab}_{max} = -\frac{(\alpha+\epsilon_{AB})(\beta+\epsilon_{AB})}{\alpha} & \textbf{B}\text{ fixes} \\
m^{Ab}_{max} = \frac{\alpha\beta}{\beta+\epsilon_{AB}} & \textbf{A}\text{ is lost} \\
m^{Ab}_{max} = -\frac{\alpha(\gamma+\epsilon_{BC})(\beta+\gamma+\epsilon_{AB}+\epsilon_{BC})}{\epsilon_{AB}(\beta+\epsilon_{AB})} & \textbf{BC}\text{ invades}
\end{cases}
\tag{C11}
$$

$$
\begin{cases}
 & \textbf{C}\text{ stays polymorphic} \\
m^{Abc}_{max} = \frac{\alpha(\beta+\gamma+\epsilon_{AB}+\epsilon_{AC})}{4(\epsilon_{AB}+\epsilon_{AC})} & \text{equilibrium collides with internal unstable} \\
m^{Abc}_{max} = -\frac{(\alpha+\epsilon_{AB}+\epsilon_{AC})(\beta+\gamma+\epsilon_{AB}+\epsilon_{AC})}{\alpha} & \textbf{BC}\text{ fixes} \\
m^{Abc}_{max} = \frac{\alpha(\beta+\gamma)}{\beta+\gamma+\epsilon_{AB}+\epsilon_{AC}} & \textbf{A}\text{ is lost} \\
m^{Abc}_{max} = \frac{\alpha(\beta+\epsilon_{AB})(\gamma\epsilon_{AB}-\beta\epsilon_{AC})}{\epsilon^2_{AB}(\beta+\gamma+\epsilon_{AB}+\epsilon_{AC})} & \textbf{Bc}\text{ invades} \\
m^{Abc}_{max} = \frac{\alpha(\gamma+\epsilon_{AC})(\beta\epsilon_{AC}-\gamma\epsilon_{AB})}{\epsilon^2_{AC}(\beta+\gamma+\epsilon_{AB}+\epsilon_{AC})} & \textbf{C}\text{ fixes} \\
 & \textbf{C}\text{ fixes} \\
m^{Ab}_{max} = \frac{(\alpha+\epsilon_{AC})(\beta+\epsilon_{AB})}{4\epsilon_{AB}} & \text{equilibrium collides with internal unstable} \\
m^{Ab}_{max} = -\frac{(\beta+\epsilon_{AB})(\alpha+\epsilon_{AB}+\epsilon_{AC})}{\alpha+\epsilon_{AC}} & \textbf{B}\text{ fixes} \\
m^{Ab}_{max} = \frac{\beta(\alpha+\epsilon_{AC})}{\beta+\epsilon_{AB}} & \textbf{A}\text{ lost} \\
m^{Ab}_{max} = \frac{(\alpha+\epsilon_{AC})(\gamma+\epsilon_{AC})(\beta\epsilon_{AC}-\gamma\epsilon_{AB})}{\epsilon^2_{AC}(\beta+\epsilon_{AB})} & \textbf{bc}\text{ invades} \\
m^{Ab}_{max} = \frac{(\alpha+\epsilon_{AC})(\beta-\gamma+\epsilon_{AB}-\epsilon_{AC})(\gamma\epsilon_{AB}-\beta\epsilon_{AC})}{(\beta+\epsilon_{AB})(\epsilon_{AB}-\epsilon_{AC})^2} & \textbf{Bc}\text{ invades}
\end{cases}
\tag{C12}
$$

C 2.4 The AC-B linkage architecture strengthens the genetic barrier.

When A and B are in loose linkage and C in tight linkage with A, the equations for the genetic barriers are given in equation (C13) if **C** appears on the island and (C14) if **C** appears

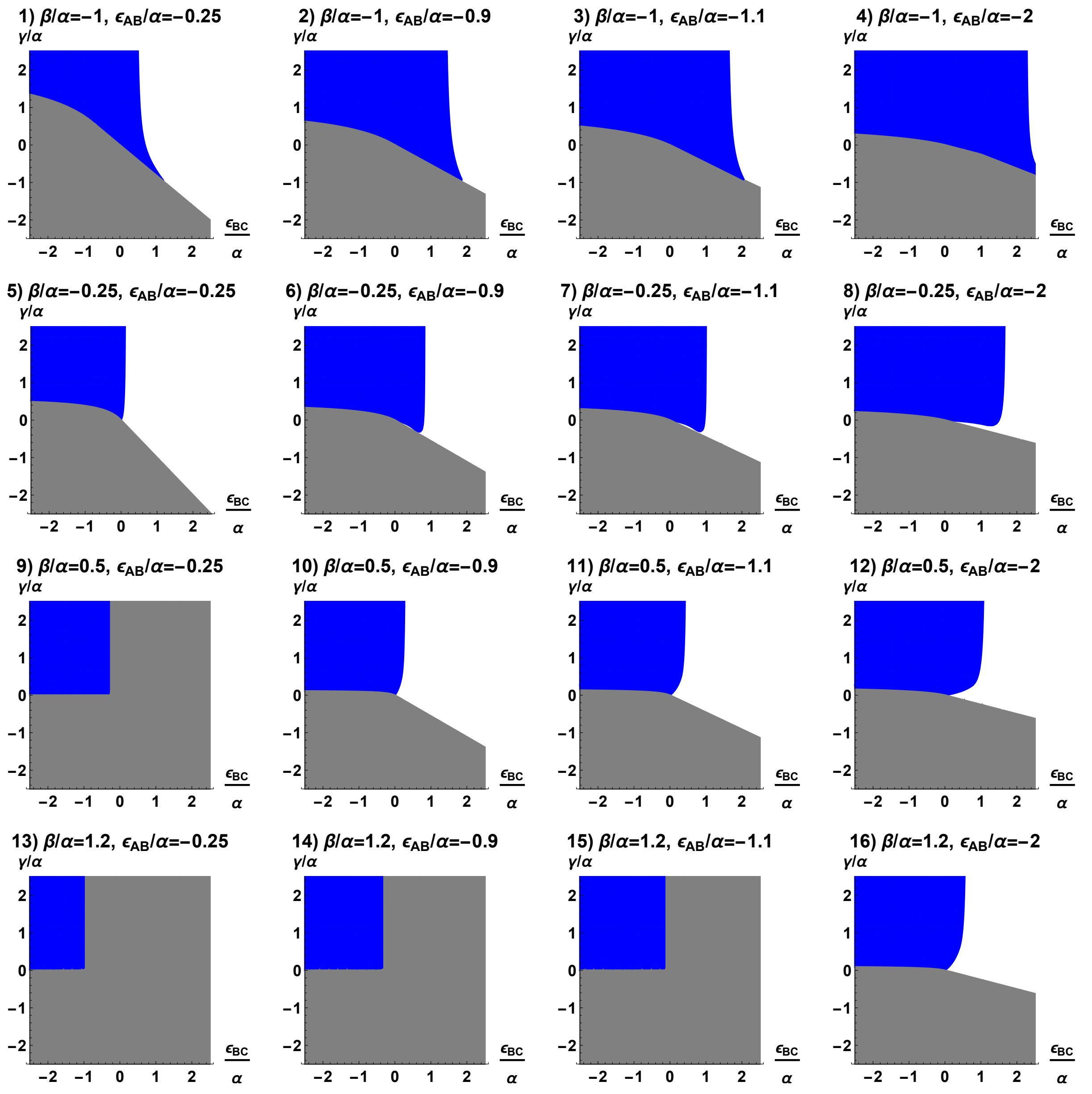

Figure C15: **Comparison between the genetic barrier for AC-B and the old one A-B, C appearing on the island**

The x-axis corresponds to the incompatibility between B and C. The y-axis corresponds to selective advantage of **C** on the island. Blue indicates that the genetic barrier is strengthened, gray that the genetic barrier is unchanged and white that it is weakened or destroyed.

on the continent. When comparing the new barrier to the two-locus barrier, we observed a strengthening of the genetic barrier under the following conditions:

- **C** appears on the island
  - If the two-locus barrier already exists ($-\epsilon_{AB} > \beta$), then either **C** is advantageous on the island and it strengthens selection against migrants by forming the "**AC**" allele or **C** is deleterious and generates positive epistasis with **B**. This last case happens mainly when **B** is also deleterious on the island and corresponds to sacrificing a small

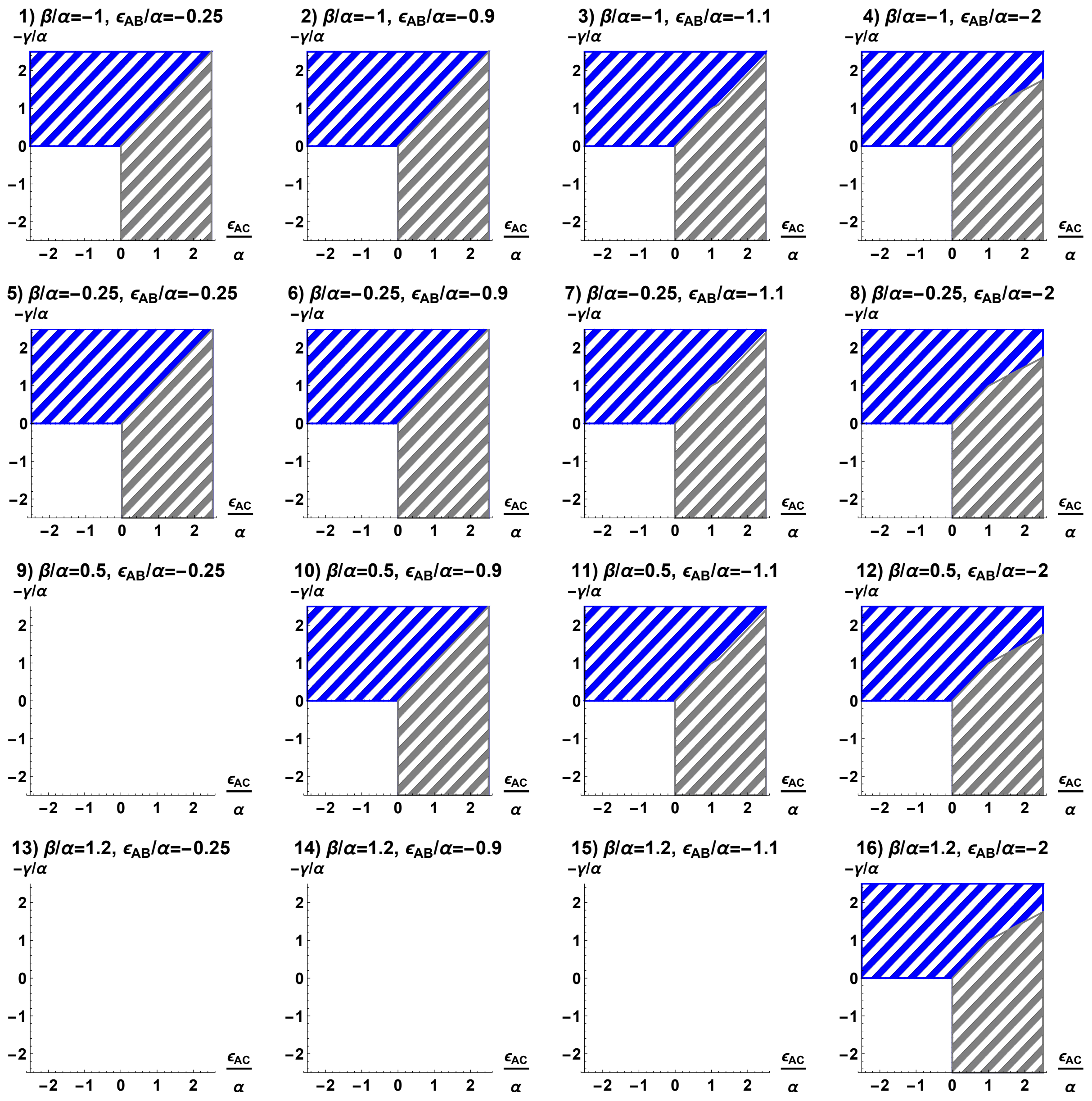

Figure C16: **Comparison between the genetic barrier for AC-B and the old one A-B, C appearing on the continent**

The x-axis corresponds to the incompatibility between A and C. The y-axis corresponds to selective advantage of **C** on the island. Stripped blue indicates a strengthening of the genetic barrier, stripped gray that the genetic barrier is strengthened through fixation of **C** on the island and white that it is weakened or destroyed.

part of the selective advantage of **A** to get rid off of most the incompatibility. Since **B** is deleterious, the epistasis hinders more than helps the genetic barrier and getting rid of it can strengthen the genetic barrier (Fig. C15, panels 1-8, 10-12,16). This is the same mechanism that happens for the two-locus 3-alleles model.

– If the two-locus barrier does not exist, then the barrier can be strengthened if **C** is advantageous and has negative epistasis with **B**. Such mutation strengthens both selection against migrants and selection against hybrids, the last one being necessary

since **B** is advantageous on the island (Fig. C15, panels 9, 13-15).

- **C** appears on the continent
  - If the two-locus barrier exists ($-\epsilon_{AB} > \beta$), mutations that are either deleterious or have positive epistasis with **A** can strengthen the genetic barrier. This corresponds again to an increase of the marginal fitness of **A** on the island either through epistasis or by making the continental allele less fit on the island (Fig. C16, panels 1-8, 10-12, 16).
  - If the two-locus barrier does not exist, then it is impossible to form a new barrier with a third locus.

$$\begin{cases} & \textbf{C} \text{ stays polymorphic} \\ m_{max}^{AbC} = -\frac{(\beta+\epsilon_{AB}+\epsilon_{BC})(\alpha+\gamma+\epsilon_{AB}+\epsilon_{BC})}{\alpha+\gamma} & \textbf{B} \text{ fixes} \\ m_{max}^{AbC} = \frac{\beta(\alpha+\gamma)}{\beta+\epsilon_{AB}+\epsilon_{BC}} & \textbf{A} \text{ and } \textbf{C} \text{ are lost} \\ m_{max}^{AbC} = \frac{(\alpha+\gamma)(\beta+\epsilon_{AB}+\epsilon_{BC})}{4(\epsilon_{AB}+\epsilon_{BC})} & \text{equilibrium collides with internal unstable} \\ m_{max}^{AbC} = \frac{\gamma(\beta+\epsilon_{AB}+\epsilon_{BC})(\alpha\epsilon_{BC}-\gamma\epsilon_{AB})}{\epsilon_{BC}^2(\alpha+\gamma)} & \textbf{C} \text{ is lost and } S_{AB} \text{ is unstable} \\ m_{max}^{AbC} = -\frac{\alpha(\beta+\epsilon_{AB}+\epsilon_{BC})(\alpha\epsilon_{BC}-\gamma\epsilon_{AB})}{\epsilon_{AB}^2(\alpha+\gamma)} & \textbf{A} \text{ is lost} \\ & \textbf{C} \text{ is lost} \\ m_{max}^{Ab} = -\frac{(\alpha+\epsilon_{AB})(\beta+\epsilon_{AB})}{\alpha} & \textbf{B} \text{ fixes} \\ m_{max}^{Ab} = \frac{\alpha\beta}{\beta+\epsilon_{AB}} & \textbf{A} \text{ is lost} \\ m_{max}^{Ab} = \frac{(\alpha-\gamma)(\beta+\epsilon_{AB})(\gamma\epsilon_{AB}-\alpha\epsilon_{BC})}{\alpha(\epsilon_{AB}-\epsilon_{BC})^2} & \textbf{C} \text{ replaces } \textbf{A} \\ m_{max}^{Ab} = \frac{\gamma(\beta+\epsilon_{AB})(\alpha\epsilon_{BC}-\gamma\epsilon_{AB})}{\alpha\epsilon_{BC}^2} & \textbf{C} \text{ invades and } S_{ABC} \text{ is unstable} \\ m_{max}^{Ab} = \frac{\alpha(\epsilon_{AB}+\beta)}{4\epsilon_{AB}} & \text{equilibrium collides with internal unstable} \end{cases} \tag{C13}$$

$$
\begin{cases}
 & \textbf{C} \text{ stays polymorphic} \\
m_{max}^{Abc} = -\frac{(\beta+\epsilon_{AB})(\alpha-\gamma+\epsilon_{AB})}{\alpha-\gamma} & \textbf{B} \text{ fixes} \\
m_{max}^{Abc} = \frac{\beta(\alpha-\gamma)}{\beta+\epsilon_{AB}} & \textbf{A} \text{ is lost and } \textbf{C} \text{ fixes, ie } \textbf{Ac} \text{ is replaced by } \textbf{aC} \\
m_{max}^{Abc} = -\frac{\alpha\gamma(\beta+\epsilon_{AB})}{\epsilon_{AB}(\alpha-\gamma)} & \textbf{ac} \text{ invades the equilibrium} \\
m_{max}^{Abc} = \frac{(\alpha-\gamma)(\beta+\epsilon_{AB})}{4\epsilon_{AB}} & \text{equilibrium collides with internal unstable} \\
 & \textbf{C} \text{ fixes} \\
m_{max}^{Ab} = -\frac{(\beta+\epsilon_{AB})(\alpha+\epsilon_{AB}+\epsilon_{AC})}{\alpha+\epsilon_{AC}} & \textbf{B} \text{ fixes} \\
m_{max}^{Ab} = \frac{\beta(\alpha+\epsilon_{AC})}{\beta+\epsilon_{AB}} & \textbf{A} \text{ is lost} \\
m_{max}^{Ab} = -\frac{\gamma(\beta+\epsilon_{AB})(\alpha+\gamma+\epsilon_{AC})}{\epsilon_{AB}(\alpha+\epsilon_{AC})} & \textbf{ac} \text{ invades and } S_{ABC} \text{ is unstable} \\
m_{max}^{Ab} = \frac{(\alpha+\epsilon_{AC})(\beta+\epsilon_{AB})}{4\epsilon_{AB}} & \text{equilibrium collides with internal unstable}
\end{cases}
\tag{C14}
$$

C 2.5 The A-B-C linkage architecture strengthens the genetic barrier.

C 2.5.1 General numerical results

For this linkage architecture, there is no analytical expression for m_{max}^{Ab}, more precisely one component of m_{max}^{Ab} can not be analytically obtained. This component corresponds to the two internal equilibria (A, B and C polymorphic) colliding. Therefore, we calculate numerically m_{max}^{Ab} for a certain number of points and extrapolate the corresponding regions.

If **C** appears on the island and interacts with allele **B**, the barrier is strengthened if **C** fulfills 2 conditions. First, its selective advantage has to be strong enough so the polymorphism at locus C is not the first one lost (the barrier is unchanged otherwise) and second, allele **C** must repress allele **B**, i.e. epistasis between **B** and **C** is negative. This case has been discussed in the main text.

If **C** appears on the continent, the barrier is strengthened if **C** generates positive epistasis with allele **A**. Since C is in loose linkage with B, there is no hitch-hiking effect for allele **B**. The C locus remains polymorphic on the island if it is strongly deleterious on the island and the interaction with allele **A** cannot compensate the direct effect of **C**. Through positive epistasis, allele **C** strengthens the marginal fitness of allele **A** and therefore the barrier is strengthened if the barrier is given either by loosing allele **A** first or the internal equilibrium is leaving through a saddle-node bifurcation (i.e. **A** lost and **B** potentially fixed).

The values calculated here for m_{max}^{Ab} (both for **C** on the island or on the continent) have been checked against the maximal migration generated by the "best" linkage architecture.

C 2.5.2 Analytical conditions

Using different Lyapunov functions of the system given in equation (1) (main text) and assuming $\epsilon_{AC} = 0$, we can determine under which conditions a three-locus equilibrium does not

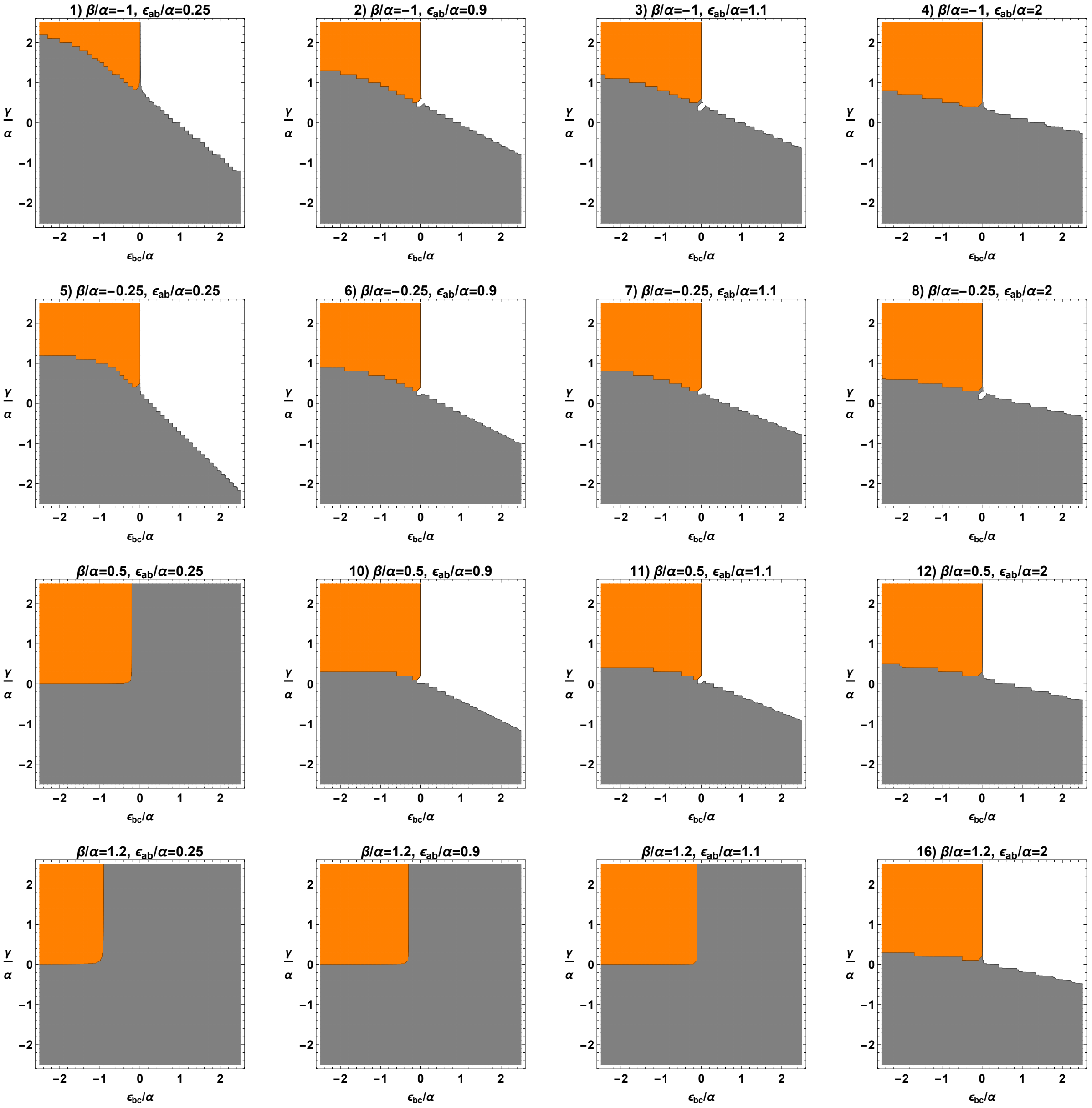

Figure C17: **Comparison between the genetic barrier for AB-C and the old one AB, C appearing on the island**

The x-axis corresponds to the incompatibility between $\mathcal{B}$ and $\mathcal{C}$. The y-axis corresponds to the selective advantage of **C** on the island. Orange indicates a strengthening of the genetic barrier, gray that the genetic barrier is unchanged and white that it is weakened or worse. m_{max}^{Ab} for the three-locus model, with all loci in loose linkage, can only be calculated numerically. For each of the panel above, m_{max}^{Ab} is calculated with an increment of 0.1 both for γ and ϵ_{BC}.

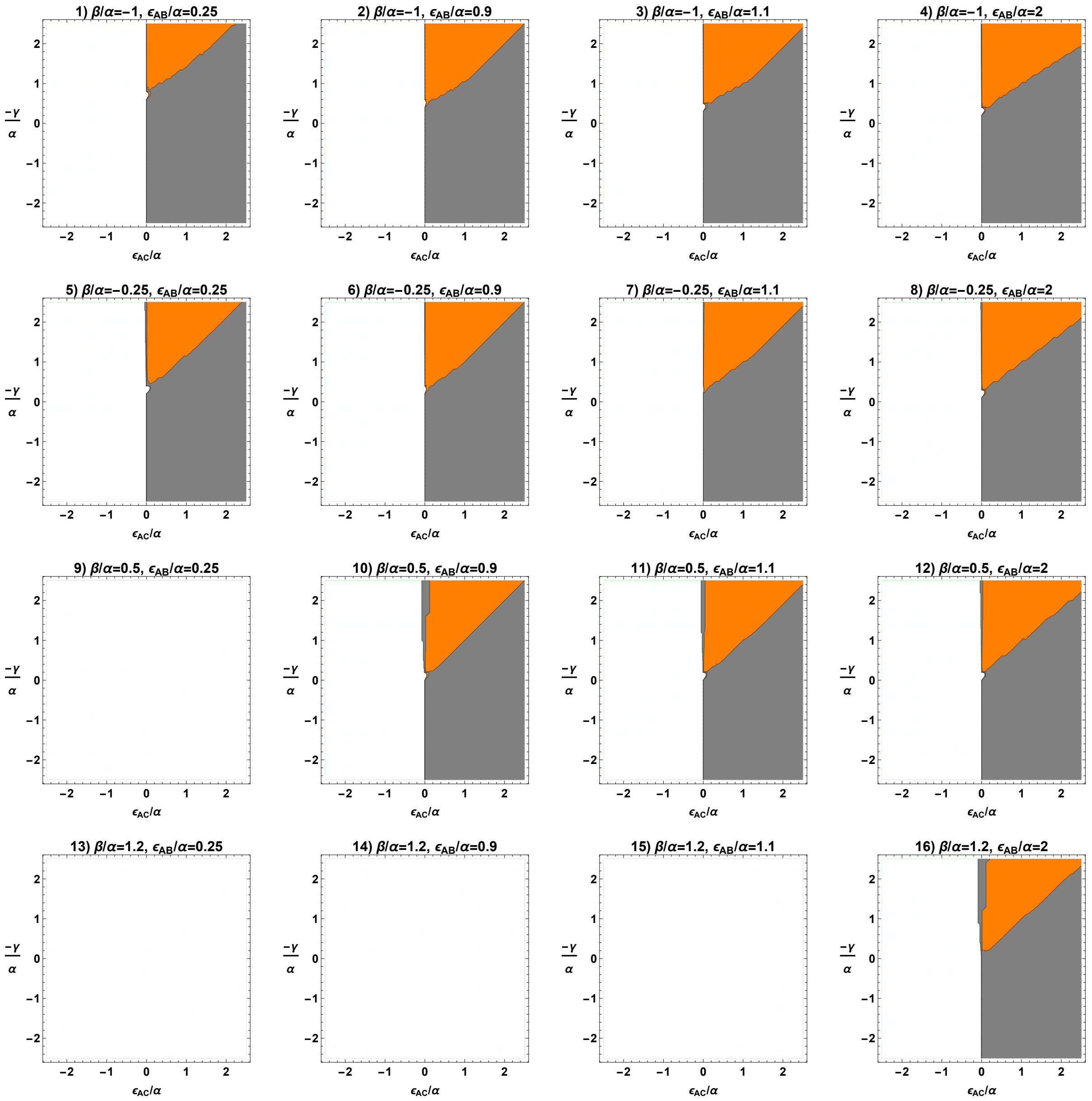

Figure C18: **Comparison between the genetic barrier for AB-C and the old one AB, C appearing on the continent**

The x-axis corresponds to the incompatibility between A and C. The y-axis corresponds to the selective advantage of **C** on the island. Orange indicates a strengthening of the genetic barrier, gray that the genetic barrier is strengthened through fixation of **C** on the island and white that the barrier is weakened or destroyed. m_{max}^{Ab} for the three-locus model can only be calculated numerically. For each of the panel above, m_{max}^{Ab} is calculated with an increment of 0.1 both for γ and ϵ_{BC}.

exist.

First, let assume $V(p) = p_A$, then

$$\dot{V} = -p_A(m + (-1 + p_A)(\alpha + p_B\epsilon_{AB})) \tag{C15}$$

Therefore, $\dot{V} \leq 0$ if $m > \alpha$ and $\epsilon_{AB} < 0$. Under this condition, $p_A \rightarrow 0$. Per symmetry, $p_C \rightarrow 0$ if $m > \gamma$ and $\epsilon_{BC} < 0$.

Therefore, we have m_{max}^{AbC}< Min$[\alpha, \gamma]$ and m_{max}^{Ab}< α. In particular, this means that as long as $\epsilon_{AB} < 0$, $\epsilon_{BC} < 0$ and $\beta < \gamma$, having all loci in loose linkage will always generate a weaker barrier, m_{max}^{Ab}, than a barrier generated by the same loci in tight linkage.

Next, let consider $V(p) = 1 - p_B$, then :

$$\dot{V} = m - p_B(m + (-1 + pB)(\beta + p_A\epsilon_{AB} + pC\epsilon_{BC})) \tag{C16}$$

$\dot{V} \leq 0$ if $\epsilon_{AB} < 0$ and :

$$\left| \begin{array}{lll} & \beta < 0 & \beta > 0 \\ \epsilon_{BC} < 0 & m > -(\beta + \epsilon_{AB} + \epsilon_{BC}) & m > -(\epsilon_{AB} + \epsilon_{BC}) \\ \epsilon_{BC} > 0 & m > -(\beta + \epsilon_{AB}) & m > -\epsilon_{AB} \end{array} \right| \tag{C17}$$

If the previous equation is true, then $p_B \rightarrow 1$.

We now consider $V(p) = \frac{1-p_B}{p_A}$.

$$\dot{V} = \frac{-1 + p_B}{p_A}(\alpha(1 - p_A) + p_B(\beta + \epsilon_{AB} + p_C\epsilon_{BC})) \tag{C18}$$

Then $\dot{V} \leq 0$ if $\alpha > 0$ and ($\epsilon_{BC} < 0$ and $\beta + \epsilon_{AB} + \epsilon_{BC} > 0$) or ($\epsilon_{BC} > 0$ and $\beta + \epsilon_{AB} > 0$), and therefore $p_B \rightarrow 1$.

Per symmetry, we also obtained if $\gamma > 0$ and $\epsilon_{AB} < 0$ and $\beta + \epsilon_{AB} + \epsilon_{BC} > 0$, then $p_B \rightarrow 1$.

Lastly, we consider $V(p) = \frac{p_C}{p_A}$

$$\dot{V} = \frac{p_C}{p_A}((-1 + p_A)(\alpha + p_B\epsilon_{AB}) - (-1 + p_C)(\gamma + p_B\epsilon_{BC})) \tag{C19}$$

Then $\dot{V} \leq 0$ if $\alpha > -\epsilon_{AB}$ and $\gamma < 0$, leading to $p_C \rightarrow 0$.

Per symmetry, we can deduce that if $\gamma > -\epsilon_{BC}$ and $\alpha < 0$, then $p_A \rightarrow 0$.

C 2.5.3 Duplication of A, all loci in loose linkage

In addition, we focus on a special case that is of biological interest: the duplication of the A locus. We assume that the copy of the A locus has been transposed somewhere else (in loose linkage). The new copy is called locus C.

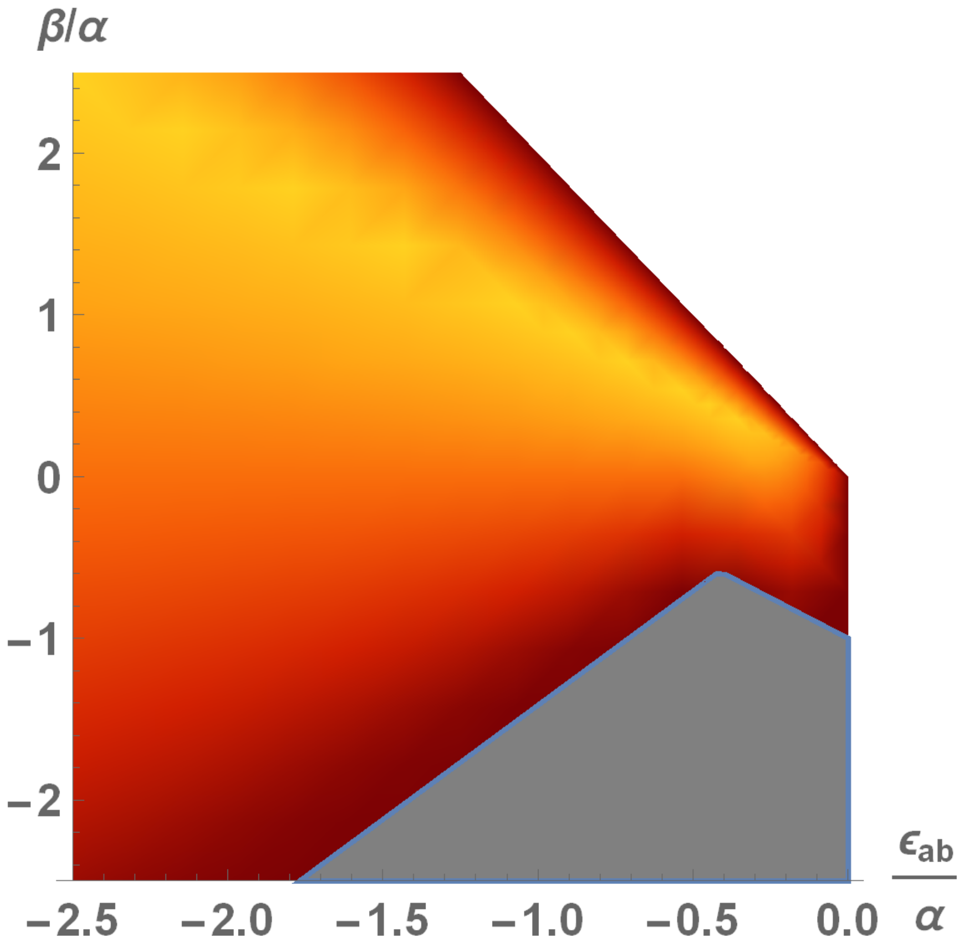

Figure C19: **Strengthening of m^{Ab}_{max} following a duplication of A in loose linkage**

The x-axis corresponds to the epistatic effect between the island adaptations and the continental one, ϵ_{AB}. The y-axis corresponds to the direct selective advantage of the continental adaptation, β. The gradient of color indicates the intensity of the strengthening of the genetic: dark red = almost no strengthening ($m^{Ab}_{max} \approx m^{Ab}_{max,0}$) to yellow = strong strengthening ($m^{Ab}_{max} \gg m^{Ab}_{max,0}$). Gray indicates that the new barrier is identical to the old one, m^{Ab}_{max}=$m^{Ab}_{max,0}$. White indicates that no barrier exists.

Similar to the two-locus case, the strongest barriers are established for a deleterious continental substitution and weak incompatibilities.

Because of the reduction in the number of parameters ($\gamma = \alpha, \epsilon_{BC} = \epsilon_{AB}$), we have been able to completely study this case analytically; the strength of the genetic barrier is given in equation (C20). Interestingly, a duplication of the island adaptation can never weaken the genetic barrier. It will either strengthen the barrier (colored area on Fig. C19) or leave it unchanged (gray area on Fig. C19).

The strength of the genetic barrier remains unchanged when selection against migrants is the main component of the genetic barrier ($\beta < 0$ and ϵ_{AB} not too strong). This situation corresponds to an already strong genetic barrier. Indeed, the genetic barrier is then mainly

due to selection against the incoming alleles (**a**, **B**, **c**) and acts (almost) independently at the different loci. Due to the selection pressure, the frequency of **B** is kept relatively low. Close to m_{max}^{Ab}, the frequencies of both alleles **A** and **C** are low. As a consequence, there is almost no epistasis expressed and **A** and **C** have no effect on each other, since they can no longer really affect the frequency of the **B** allele. For this scenario, m_{max}^{Ab} corresponds to the simultaneous loss of alleles **A** and **C**.

The other possible outcome is a strengthening of the genetic barrier. This strengthening is the most efficient when **B** is also advantageous on the island and the incompatibility is just strong enough to allow the DMI to persist in the first place ($\epsilon_{AB} \approx -\beta$, Fig. C19, yellow ridge). In this situation, the frequency of allele **B** increases quickly with m, and the new duplicated allele, **C**, helps to repress allele **B**. The cost of hybrids is then shared across two loci instead of one, leading to the observed strengthening of the genetic barrier. Because of the symmetry between both island adaptations, allele **C** is always lost exactly at the same time than allele **A**, therefore m_{max}^{AbC} is completely equivalent to m_{max}^{Ab}, its expression is given in equation C20. In this equation, the first term corresponds to loosing alleles **A** and **C**, the second one to loosing allele **b**, i.e **B** fixes on the island despite the incompatibilities and the last term corresponds to a saddle-node bifurcation.

$$
\begin{cases}
m_{max}^{AbC} = & \frac{\alpha\beta}{\epsilon_{AB}+\beta} \\
& \text{If } \beta^2 \geq 2\epsilon_{AB}^2 \text{ and } \left((\alpha+\beta \leq -\epsilon_{AB} \text{ and } \alpha^2 > -\epsilon_{AB}(2\alpha-\epsilon_{AB})\right) \text{ or } \alpha^2 \leq -\epsilon_{AB}(2\alpha-\epsilon_{AB})) \\
m_{max}^{AbC} = & \frac{-(\beta+2\epsilon_{AB})(\alpha+\epsilon_{AB})}{\alpha-\epsilon_{AB}} \\
& \text{If } \alpha^2 > -\epsilon_{AB}(2\alpha-\epsilon_{AB}) \text{ and} \\
& \left(\left(\beta^2 \geq 2\epsilon_{AB}^2 \text{ and } \left(\left(\beta-\sqrt{2}\epsilon_{AB} \leq 0 \text{ and } \alpha+\beta \geq -\epsilon_{AB}\right) \text{ or } \beta \geq -\sqrt{2}\epsilon_{AB}\right)\right) \text{ or } \left(\beta^2 < 2\epsilon_{AB}^2\right)\right) \\
m_{max}^{AbC} = & \frac{\left(-3+2\sqrt{2}\right)\alpha(\beta+2\epsilon_{AB})}{-\epsilon_{AB}} \\
& \text{If } \beta < -2\epsilon_{AB} \text{ and } \beta^2 < 2\epsilon_{AB}^2 \text{ and } \alpha(\alpha+2\epsilon_{AB}) \leq \epsilon_{AB}^2 \\
m_{max}^{AbC} = & 0 \text{ Else}
\end{cases}
\tag{C20}
$$

C 2.5.4 Duplication of the B locus

Here, we assume that the new locus, C, results from a duplication of the B locus in loose linkage. Equation (C21) gives the expression of the genetic barrier. Assuming that a genetic barrier exists between the A and B loci, the duplication of the B locus always results in a weakening of the genetic barrier. The expression for the strength of the genetic barrier is given

in equation (C21). Indeed, the new allele generates an extra cost for allele **A** leading to the observed weakening.

$$m_{max}^{Abc} = \begin{cases} \frac{\left(3-2\sqrt{2}\right)\alpha(\beta+\epsilon_{AB})}{\epsilon_{AB}} & \text{if } \beta+\epsilon_{AB}<0 \text{ and } \beta^2<2\epsilon_{AB}^2 \text{ and } \left(\alpha^2+4\alpha\epsilon_{AB}+2\epsilon_{AB}^2<0 \text{ or } \alpha+\epsilon_{AB}\le 0\right) \\ -\frac{(\alpha+2\epsilon_{AB})(\beta+\epsilon_{AB})}{\alpha+\epsilon_{AB}} & \text{if } \left(\beta\ge 0 \text{ or } \beta^2<2\epsilon_{AB}^2 \text{ or } \alpha+\beta+2\epsilon_{AB}>0\right) \text{ and } \beta+\epsilon_{AB}<0 \\ & \text{and } \alpha^2+4\alpha\epsilon_{AB}+2\epsilon_{AB}^2\ge 0 \text{ and } \alpha+\epsilon_{AB}>0 \\ \frac{\alpha\beta}{\beta+2\epsilon_{AB}} & \text{if } \beta^2\ge 2\epsilon_{AB}^2 \text{ and } \beta+\epsilon_{AB}<0 \text{ and } \left(\alpha+\beta+2\epsilon_{AB}<0\right. \\ & \left.\text{or } \alpha^2+4\alpha\epsilon_{AB}+2\epsilon_{AB}^2<0 \text{ or } \alpha+\epsilon_{AB}\le 0\right) \end{cases} \tag{C21}$$

C 3 Extension of the genetic barrier: strongest barrier

C 3.1 Which linkage architecture provides the strongest genetic barrier? two-locus model

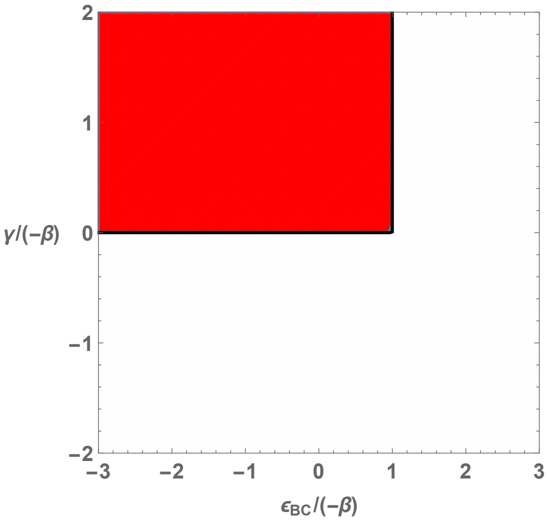

(a) **B** deleterious and **BC** epistasis

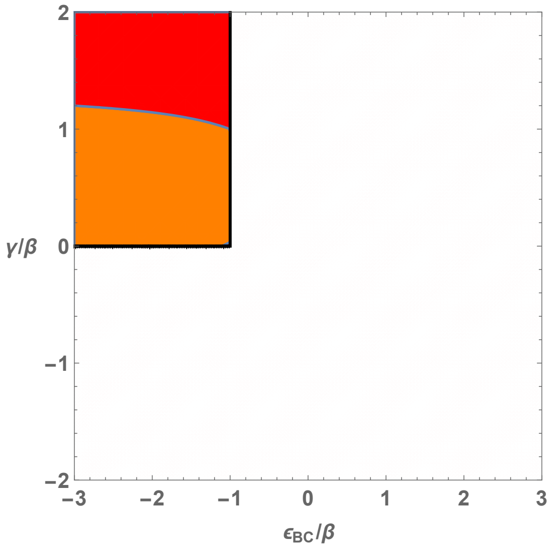

(b) **B** advantageous and BC epistasis

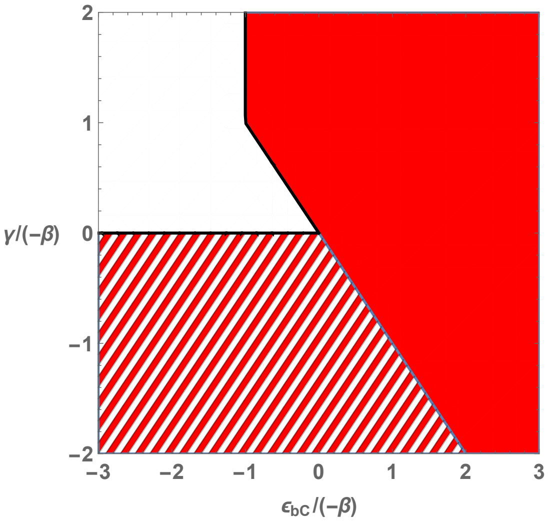

(c) **B** deleterious and **bC** epistasis

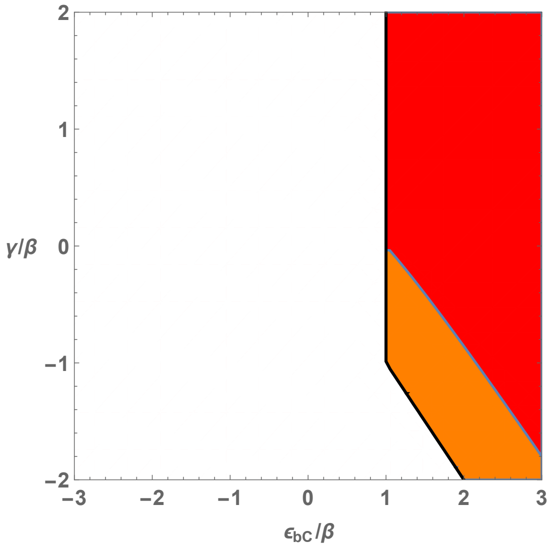

(d) **B** advantageous and **bC** epistasis

Figure C20: **Linkage architecture forming the strongest genetic barrier, following the invasion of C**

For each panel, the x-axis corresponds to the epistasis between **C** and its interacting allele, **B** for the first row and **b** for the second row. The y-axis corresponds to selective advantage of allele **C** on the island. The different colors indicate the linkage architecture and location a **C** mutation should appear to maximize m^b_{max}. White area means that **C** never strengthens the barrier, red that **C** should appear in tight linkage and orange in loose linkage. Fully filled areas indicate that **C** should appear on the island and stripped areas on the continent.

C 3.2 Which linkage architecture provides the strongest genetic barrier? three-locus model

C 3.2.1 Analytical results, C appears on the island

Assuming that **B** is deleterious on the island ($\beta < 0$), then having all loci in tight linkage is always optimal: The strength of the genetic barrier is given by $m_{max}^{Ab} = \alpha - \beta + \gamma$. Indeed, if all loci are in loose linkage, we know that $m_{max}^{Ab} < \alpha$ and therefore as long as $\beta < \gamma$, having all loci in tight linkage always generates the strongest barrier. Otherwise, for all other linkage architectures; we check that $m_{max}^{Abc} \leq \alpha - \beta + \gamma$ if $\epsilon_{BC} < 0$ (since we have analytical expression). In addition, if **C** is lost then, $m_{max}^{Abc} \leq m_{max,0}^{Ab} \leq \alpha - \beta$. Therefore, we have analytical evidence that if **C** appears on the island and interacts negatively with **B**, then having all loci in tight linkage always forms the strongest barrier as long as allele **B** is deleterious on the island.

Another way to see this result for the 2 loci in tight linkage and 1 in loose linkage cases is by the following reasoning: each of these cases corresponds to having 2 loci in loose linkage, with one locus having 4 alleles. We have shown previously than we only maintain at most 2 alleles,

- A and C are in tight linkage. Therefore m_{max}^{Ab} in these conditions is (at best) equal to the barrier generated by the two-locus 2-alleles model with alleles **ac** and **AC** (m_{max}^{AbC}), or alleles **ac** and **Ac** (m_{max}^{Abc}) at locus A, i.e. $m_{max}^{Ab} \leq \text{Max}[m_{max}^{AbC}, m_{max}^{Abc}]$. Using the limits of the two-locus model, we obtain $m_{max}^{Ab} < \alpha + \gamma$.

- B and C are in tight linkage. Therefore m_{max}^{Ab} in these conditions is (at best), equal to the barrier generated by the two-locus 2-alleles model with alleles **Bc** and **bC** (m_{max}^{AbC}), or alleles **Bc** and **bc** (m_{max}^{Abc}) at locus B, i.e. $m_{max}^{Ab} \leq \text{Max}[m_{max}^{AbC}, m_{max}^{Abc}]$. Using the limits of the two-locus model, we obtain $m_{max}^{Ab} < \alpha$.

- A and B are in tight linkage. Therefore m_{max}^{Ab} in these conditions is (at best) equal to the barrier generated by the two-locus 2-alleles model with alleles **Ab** and **aB** (m_{max}^{AbC}) at locus B, i.e. $m_{max}^{Ab} \leq \text{Max}[m_{max}^{AbC}, m_{max}^{Abc}]$. Using the limits of the two-locus model, we obtain $m_{max}^{Ab} < \text{Max}[\alpha - \beta, \gamma]$.

The genetic barrier, described in the previous cases, is therefore always smaller or equal to $\alpha - \beta + \gamma$ and therefore, as long as **B** is deleterious, the optimal architecture is given by having all loci in tight linkage.

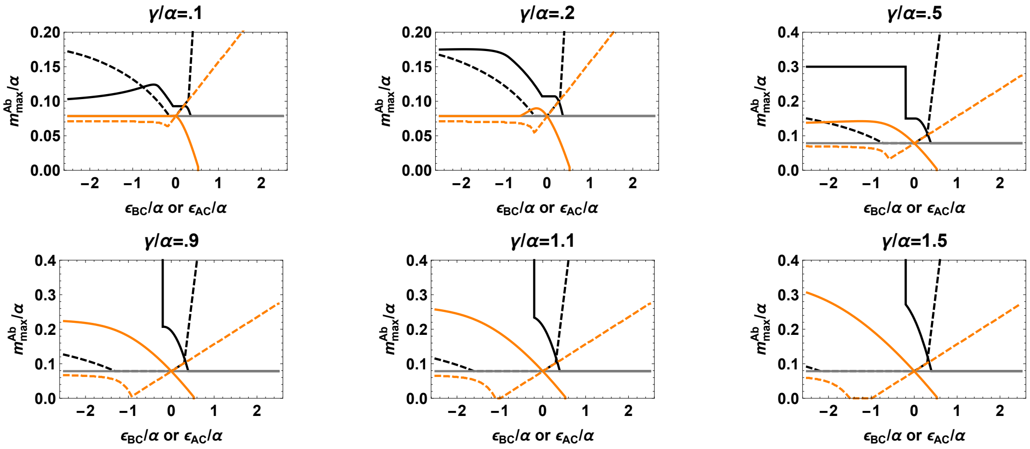

Figure C21: **Comparison between m^{Ab}_{max} for the best linkage architecture and all loci in loose linkage**

We represent the strength of the genetic barrier established by the best linkage architecture (black) and all loci in loose linkage (orange), for **C** appearing on the island (solid lines) or on the continent (dashed lines) as a function of epistasis. We use $\frac{\beta}{\alpha} = -.5$ and $\frac{\epsilon_{AB}}{\alpha} = -1.75$.

In addition, if having the two loci, A and B in tight linkage provides the best barrier in the two-locus model, then we can prove that if **C** appears on the island, the best barrier will be formed if C is in tight linkage with the other two loci.

Using Mathematica, we were able to show that m^{AbC}_{max}, for all 2 tight linkage + 1 loose linkage architectures, is smaller than $\alpha-\beta+\gamma$. m^{AbC}_{max} for any 2 tight linkage +1 loose linkage architecture can be decomposed into 2 parts: the dynamics between the loci in loose linkage, that are given in Bank et al. [2012], plus some internal allelic effect at the locus that has 4 alleles. For all three architectures, it was sufficient and easier to show that the "two-locus" dynamics (with choosing the best allele as the second one) was generating a weaker barrier than all 3 loci in tight linkage. Any additional "intra loci effect" only weakens the barrier. We also only calculate it for m^{AbC}_{max}, as it is obvious that if $m^{Ab}_{max,0} \leq \alpha - \beta$, then m^{Abc}_{max} is also smaller.

If all loci are in loose linkage, this is also obviously true as long as $\gamma > \beta$ (as m^{AbC}_{max} for the 3 loci in loose linkage is smaller than α). Then, if $\gamma < \beta$, C in loose linkage means first that **C** has to pay some migration cost before strengthening the barrier through selection against hybrids. Because of these two factors, the barrier will only be strengthened from a fraction of the amount of the selective advantage of **A**, whereas if all loci are in tight linkage, the barrier is strengthened by γ. Therefore, having all loci in loose linkage under these conditions will always generate a weaker barrier then having all of them in tight linkage.

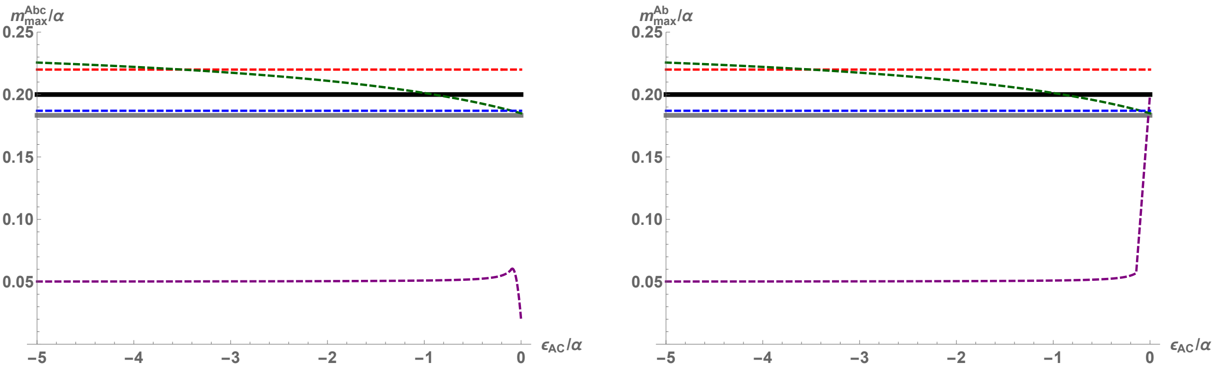

Figure C22: **Genetic barrier if C appears on the continent**

We represent the strength of the genetic barrier m_{max}^{Abc} (left) and m_{max}^{Ab} (right) if **C** appears on the continent, as a function of the epistasis between **A** and **C**. Each color corresponds to a different architecture: red to all loci in tight linkage, purple to **AB** in tight linkage and **C** in loose linkage, blue to **AC** in tight linkage and **B** in loose linkage and green to **BC** in tight linkage and **A** in loose linkage. The black and gray lines correspond to $m_{max,0}^{Ab}$ respectively if **A** and **B** are in tight linkage or in loose linkage. Here, we use $\frac{\beta}{\alpha} = .8$, $\frac{\gamma}{\alpha} = -.02$ and $\frac{\epsilon_{AB}}{\alpha} = -3$

C 3.2.2 Analytical results, C appears on the continent

In this section, we assume that **C** appears on the continent and generates negative epistasis with allele **A**. In addition, we assume here that **C** is maladaptive on the island ($\gamma < 0$).

If $\beta < 0$, then we were able to show, using Mathematica, that $m_{max}^{Abc} \leq \alpha - \beta - \gamma$, for all architectures, except having all loci in tight linkage. If all loci are in loose linkage, then m_{max}^{Abc} $\leq \alpha$ (this can be shown using $V(p) = p_A$ as a Lyapunov function). Therefore, as long as all continental adaptations are deleterious on the island and in the absence of positive epistasis with the island adaptation, having all loci in tight linkage always generates the strongest barrier.

Using Mathematica, we were able to prove that if having **A** and **B** in tight linkage provides a stronger genetic barrier than having both loci in loose linkage. Therefore, having all loci in tight linkage provides a stronger barrier than having **A** and **B** in tight linkage and **C** in loose linkage, $m_{max}^{Abc} < \alpha - \beta - \gamma$. This is also true if **A** and **C** are in tight linkage and **B** is in loose linkage.

However, if **B** and **C** are in tight linkage, and **A** is in loose linkage, this is no longer true, as illustrated in Fig. C22 (green dashed line above the red one).

Therefore, having **C** appearing on the island or on the continent is not symmetric in this regard.

	**abc**	**Abc**	**aBc**	**abC**	**ABc**	**AbC**	**aBC**	**ABC**
Fitness:	0	α	β	γ	$\alpha+\beta+\epsilon_{AB}$	$\alpha+\gamma$	$\beta+\gamma+\epsilon_{BC}$	$\alpha+\beta+\gamma+\epsilon_{AB}$

Table C2: **Special fitness table where the epistasis between B and C is only expressed in the absence of A**

C 3.3 Alternative model where having all loci in loose linkage is optimal.

Fig. C23 illustrates when having all loci in loose linkage can provide the strongest genetic barrier (the orange line is the top one). To obtain such results, the fitness scheme used is presented in table C2. The difference relative to the fitness scheme used in the main part concerns the effect of allele **C**, and more precisely its epistasis. **C** interacts with allele **B** but only in the absence of allele **A**. Therefore, **C** needs to be in loose linkage with both loci to be able to express its epistasis. This corresponds to the existence of a three-locus interactions term that cancels the effect of the interactions of **B** and **C** in presence of allele **A** ($\epsilon_{ABC} = -\epsilon_{BC}$).

Still despite designing a fitness scheme that should favor having all loci in loose linkage, this linkage architecture forms the strongest barrier only over a small range of parameters. Indeed, to observe such behavior, first we have to avoid having **AbC** as the optimal haplotype on the island and therefore γ (Fig. C23(c)) has to be not too large and β quite large (Fig. C23(a)). Then epistasis between **A** and **B** needs to be not too strong (Fig. C23(b)), otherwise allele **A** represses efficiently allele **B**, and therefore increasing the marginal fitness of allele **A** by attaching allele **C** to it, is the best option. Lastly, the epistasis between **B** and **C** has to be strong enough (Fig. C23(d)), such that it is more efficient to have **C** in loose linkage than in tight linkage with allele **b**, where its reduces the selective advantage of allele **B** on the island. However, too much epistasis is not optimal either, as m^{Ab}_{max} converges to $\frac{\gamma}{4} =$ (here 0.1375) as the incompatibility becomes lethal.

C 3.4 m_{max} as a function of recombination

Fig. C24 and C25 represent the maximal migration rate for stability of the DMI between **A** and **B**, m^{Ab}_{max}, for arbitrary recombination rate. Indeed, in the main text, we only consider limited cases of recombination (tight linkage and loose linkage), because they are the only analytically solvable cases and also because already in the two-locus model, understanding the limiting cases provides a good description of the full system.

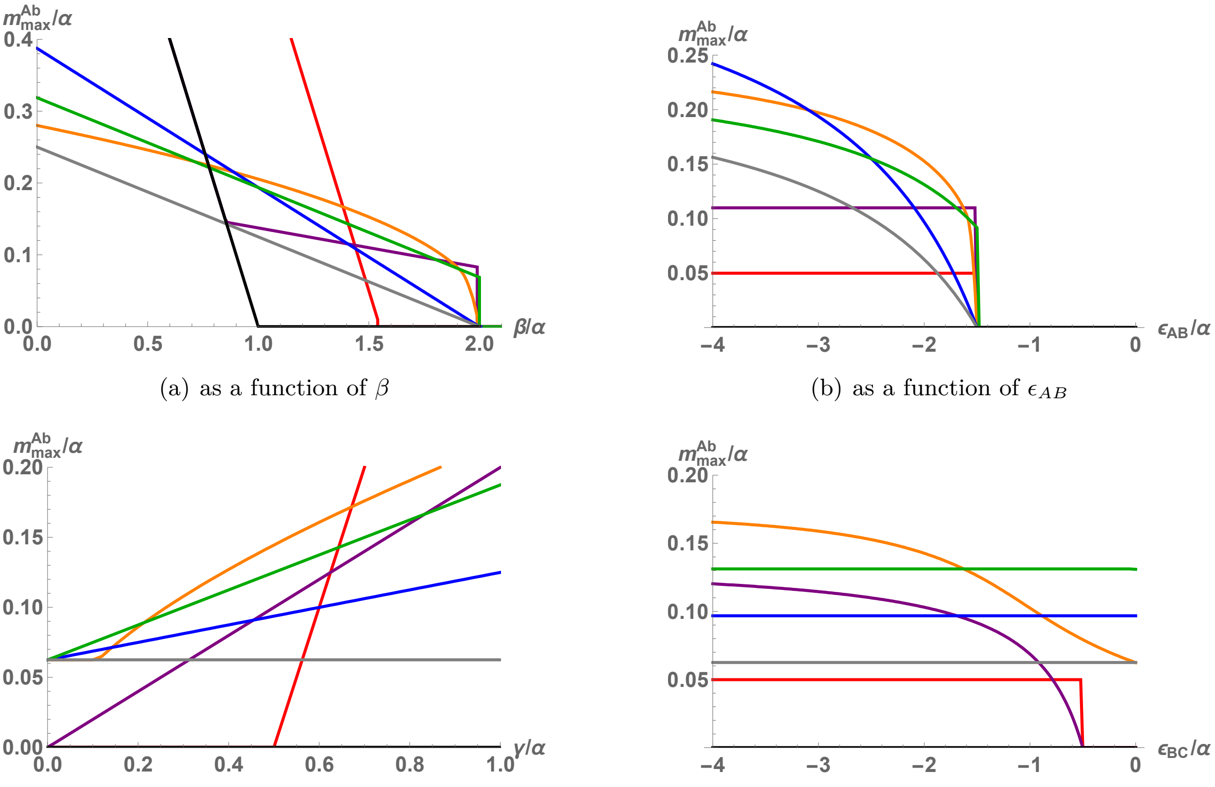

(a) as a function of β

(b) as a function of ϵ_{AB}

(c) as a function of γ

(d) as a function of ϵ_{BC}

Figure C23: m^{Ab}_{max} **for all five linkage architectures, if the new allele C only interacts epistatically with aB**

We represent m^{Ab}_{max} as a function of all parameters of the system, varying only one each time and using for the following values for non-varying ones: $\frac{\beta}{\alpha} = 1.5, \frac{\gamma}{\alpha} = 0.8, \frac{\epsilon_{AB}}{\alpha} = -2, \frac{\epsilon_{BC}}{\alpha} = -2.5$. Each color corresponds to a different linkage architecture: black to **AB**, gray to **A-B**, red to **ABC**, purple to **AB-C**, blue to **AC-B**, green to **A-BC** and orange to **A-B-C**.

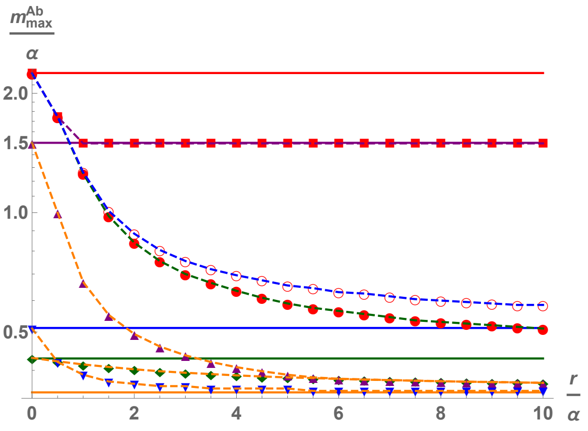

(a) $\frac{\beta}{\alpha} = -0.5$, $\frac{\gamma}{\alpha} = 0.75$, $\frac{\epsilon_{AB}}{\alpha} = -1.75$ and $\frac{\epsilon_{BC}}{\alpha} = -1.2$

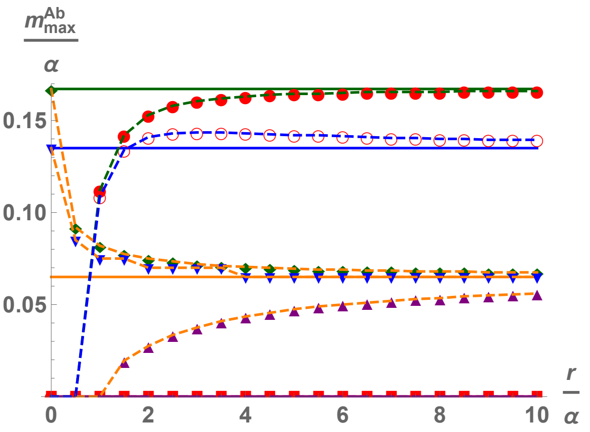

(b) $\frac{\beta}{\alpha} = 0.75$, $\frac{\gamma}{\alpha} = 0.5$, $\frac{\epsilon_{AB}}{\alpha} = -2$ and $\frac{\epsilon_{BC}}{\alpha} = 0.8$

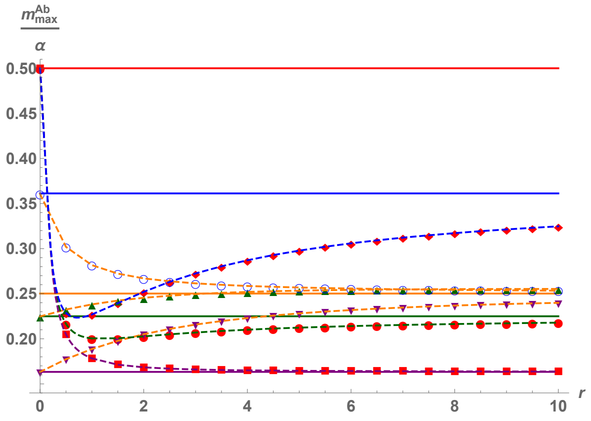

(c) $\frac{\beta}{\alpha} = 1.2$, $\frac{\gamma}{\alpha} = 0.7$, $\frac{\epsilon_{AB}}{\alpha} = -5$ and $\frac{\epsilon_{BC}}{\alpha} = -3$

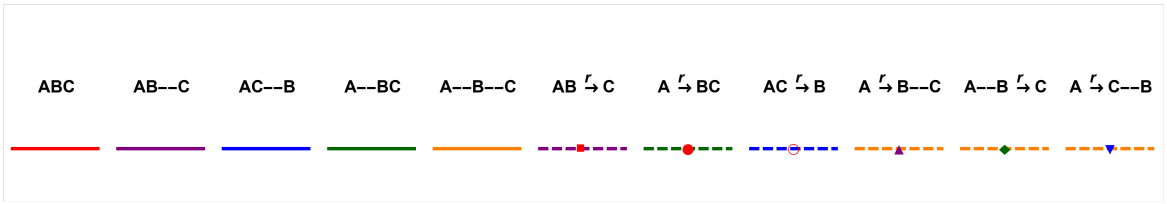

Figure C24: m^{Ab}_{max} **as a function of recombination, C appears on the island**

We represent m^{Ab}_{max} as a function of recombination. Each line follows a precise scheme: the color of the symbols corresponds to the linkage architecture for $r = 0$, the dashed line, a guide for the eye, is colored to correspond to the linkage architecture at $r \to \infty$. For example, a orange dashed line with green symbols, indicates that we start in the following configuration A-BC and ends with all loci in loose linkage, A-B-C). Horizontal solid lines correspond to limited cases studied previously, with the color scheme indicated in the legend. For the numerical evaluations, we use $(r_{ac}, r_{bc}, r_{ab}) = 500\alpha$ to represent loose linkage.

Here, we observe that for all the different architectures, we always observe convergence to the limiting cases, both for $r \to 0$ and $r \to \infty$. In most cases, similar to Bank et al. [2012], m^{Ab}_{max} is an monotonous function of recombination.

In addition, departure from $r = 0$ is always relatively fast (as long as the genetic barrier exists in tight linkage), making this configuration rare in genomes. The tight linkage behavior is

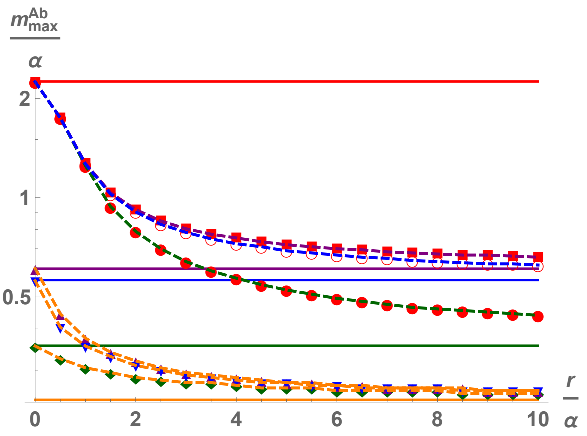

(a) **C** appears on the continent; $\frac{\beta}{\alpha} = -0.5$, $\frac{\gamma}{\alpha} = -0.75$, $\frac{\epsilon_{AB}}{\alpha} = -1.75$ and $\frac{\epsilon_{BC}}{\alpha} = -1.2$

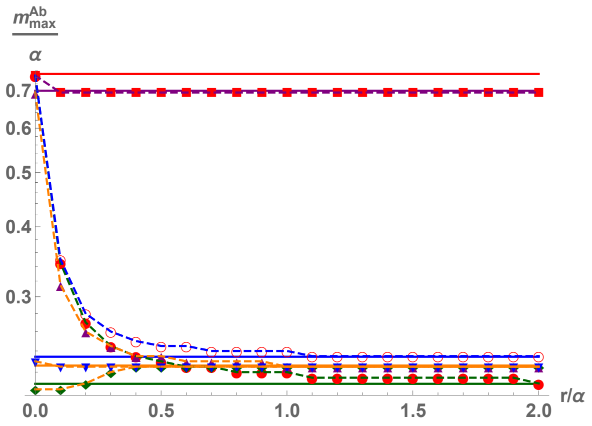

(b) **C** appears on the continent; $\frac{\beta}{\alpha} = 0.75$, $\frac{\gamma}{\alpha} = -0.5$, $\frac{\epsilon_{AB}}{\alpha} = -2$ and $\frac{\epsilon_{BC}}{\alpha} = 0.45$

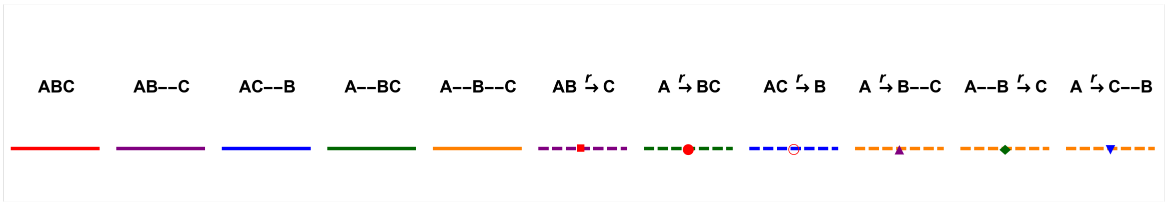

Figure C25: m_{max}^{Ab} **as a function of recombination, C appears on the continent**

We represent m_{max}^{Ab} as a function of recombination. Each line follows a precise scheme: the color of the symbols corresponds to the linkage architecture for $r = 0$, the dashed line, a guide for the eye, is colored to correspond to the linkage architecture at $r \to \infty$. For example, a orange dashed line with green symbols, indicates that we start in the following configuration A-BC and ends with all loci in loose linkage, A-B-C. Horizontal solid lines correspond to limited cases studied previously, with the color scheme indicated in the legend. For the numerical evaluations, we use $(r_{ac}, r_{bc}, r_{ab}) = 500\alpha$ to represent loose linkage.

therefore mainly valid for inversions or loci that are really next to each other in the genome. In the opposite limit, we always observe an asymptotic convergence to loose linkage. This indicates that as long as linkage disequilibrium is weak, the behavior of these loci can be explained by ignoring the linkage disequilibrium and assuming they are in loose linkage. This makes the loose linkage cases especially important since a large portion of the genome can be explained under this regime.

We notice one example where a local maximum is generated for an intermediate recombination rate (Fig. C24(b), dashed blue line with red circle, i.e. going from ABC to AC-B). If we consider the two-locus 2 alleles model (with **ac** and **AC** as the two alleles), then m_{max}^{AbC} is simply a monotonic decreasing function of recombination as predicted in Bank et al. [2012]. However, here, we have 4 possible alleles at locus A. When $r = 0$, the optimal haplotype is **aBC** and not **AbC**, therefore $m_{max}^{AbC} = 0$. As recombination increases, the advantage of allele **aC** over allele **AC** decreases and vanishes. Therefore, m_{max}^{AbC} is given by two components: an increasing

function corresponding to allele **AC** replacing allele **aC** and a decreasing one corresponding to the dynamics between the two loci A (technically AC) and B, generating this maximal optimum when we switch from one regime to the other.

References

C. Bank, R. Bürger, and J. Hermisson. The Limits to Parapatric Speciation: Dobzhansky–Muller Incompatibilities in a Continent–Island Model. *Genetics*, 191(3):845–863, 2012.
